# Suppressing chondrocyte cuproptosis by syringaresinol-4-*O*-*β*-d-glucoside alleviates gouty arthritis

**DOI:** 10.3389/fphar.2025.1565422

**Published:** 2025-05-09

**Authors:** Shaotian Fu, Han Du, Xiao Ling, Hanyi Wang, Jianan Chen, Hang Zhang, Wugui Chen, Chengzhao Liu, Hailong Ma, Chengshou Lin, Peixiang Ma, An Qin

**Affiliations:** ^1^ Shanghai Key Laboratory of Orthopedic Implants, Department of Orthopedics, Ninth People’s Hospital, Shanghai Jiaotong University School of Medicine, Shanghai, China; ^2^ Department of Orthopaedics, Mindong Hospital Affiliated Fujian Medical University, Ningde, Fujian, China

**Keywords:** gouty arthritis, cuproptosis, syringaresinol-4-O-β-D-glucoside, monosodium urate crystals, inflammatory cytokines, chondrocyte

## Abstract

**Background:**

Gouty arthritis is a rheumatic disease characterized by synovial inflammation and cartilage damage. Current therapeutic options for gouty arthritis, such as colchicine, primarily relieve the symptoms, which makes treatment challenging.

**Methods:**

We employed an in vitro co-culture system of chondrocytes and macrophages to simulate gouty arthritis and screen compounds that can inhibit monosodium urate (MSU) associated macrophage inflammation and chondrocytes degeneration. We further elucidated the cuproptosis mechanism in chondrocytes by qPCR and Western blotting analyses. Both acute and chronic gouty arthritis mouse models were established to evaluate the therapeutic efficacy of candidate drugs against gouty arthritis.

**Results:**

MSU upregulates the expression of inflammatory cytokines in macrophages and simultaneously induces cuproptosis in chondrocytes. By screening 24 compounds, we identified syringaresinol-4-O-β-d-glucoside (SSG), a furanoid lignan, as a potent inhibitor of macrophage-mediated inflammation and chondrocyte cuproptosis. Mechanistically, SSG inhibited MSU-induced activation of the NF-κB and NLRP3 pathways in macrophages. Furthermore, SSG regulated the expression of sulfur-linked mitochondrial enzymes (e.g., DLAT) in the cuproptosis pathway, thereby inhibiting the upstream regulator FDX1 in chondrocytes. SSG not only alleviated inflammatory pain but also protected against cartilage damage and improved motor dysfunction in the mice models of acute and chronic gouty arthritis.

**Conclusion:**

SSG can serve as a promising therapeutic option for gouty arthritis in clinical settings by suppressing inflammation and preserving cartilage integrity.

## Graphical Abstract

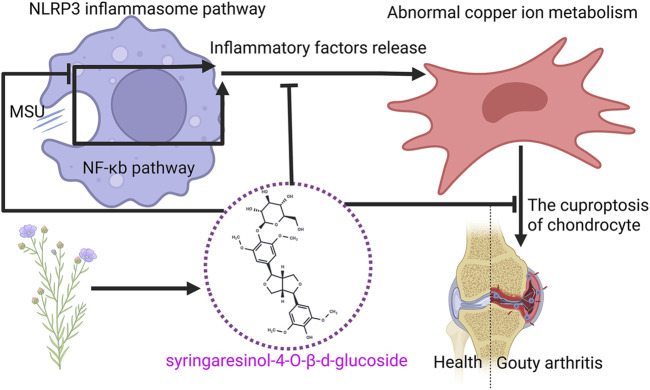

## 1 Introduction

Gout is an inflammatory disease caused by the deposition of monosodium urate crystals (MSU) in joint or non-joint areas (Neogi, 2016). Gout mainly presents with severe pain, redness, swelling, and movement disorders in the lower limb joints. Gout is often self-limiting, and its symptoms typically resolve within 7–14 days, entering an asymptomatic phase. However, underlying health issues and uncontrolled hyperuricemia often induce the recurrence of gout, leading to chronic gouty arthritis (Dalbeth et al., 2021). Thus, gout imposes a significant burden on both society and families (Anderson et al., 2021).

MSU acts as a damage-associated molecular pattern (DAMP) that stimulates innate immune responses. After reaching its solubility saturation point (428 μM), uric acid crystals can precipitate due to changes in temperature, injury, or underlying diseases (Sivera et al., 2017). These crystals can regulate the mechanical environment, which amplifies the inflammatory response and even damages the joints (Zheng et al., 2024; Feng et al., 2024; Xu et al., 2023). These crystals then stimulate surrounding macrophages via Toll-like receptors (TLRs) and subsequently activate the NF-κB pathway, which leads to the release of IL-1β. Activation of interleukin-1 receptors triggers a cascade of downstream signals, including pro-inflammatory cytokines and chemokines, which recruit neutrophils and other immune cells (So and Martinon, 2017). In chronic gout, the formation of tophus can damage the joint structure. Furthermore, persistent inflammation induces the release of inflammatory cytokines, reactive oxygen species (ROS), and nitric oxide (NO). These factors enhance the expression of degenerative enzymes, such as matrix metalloproteinase-3 (MMP-3) in chondrocytes, accelerating cartilage degeneration. Furthermore, they promote chondrocyte autophagy and senescence, leading to chondrocyte apoptosis (Keller and Mandell, 2021).

In addition to inflammatory cytokines, new mechanisms have been implicated in cartilage degeneration (Zhou et al., 2024). Copper ions regulate cellular oxidative stress. The cellular copper metabolism relies on proteins involved in copper ion intake, such as CTR1, and proteins involved in copper ion efflux, such as ATP7A and ATP7B. Copper ions excessively accumulated in the intracellular space can bind to thiolase-containing mitochondrial enzymes (e.g., DLAT) in the tricarboxylic acid (TCA) cycle, which activates the upstream regulatory factor FDX1 and enhances toxic stress and cell death. This process is known as cuproptosis (Cobine and Brady, 2022). The initial step of cuproptosis occurs in mitochondria, where dysregulation of the TCA cycle impairs cellular energy metabolism and damages the mitochondrial membrane. Furthermore, copper ion accumulation exacerbates the loss of Fe-S clusters and induces cell death. Glutathione, Hsp70, and Hsp90 can mitigate oxidative stress following copper ion accumulation in the intracellular space (Chen et al., 2023). Previous studies have reported that inflammatory cell infiltration and the release of inflammatory cytokines can lead to the depletion of glutathione and heat shock proteins (Jhang et al., 2015). Moreover, stimulating chondrocytes with interleukin-1 was shown to downregulate ATP7B and FDX1 levels and induce copper ion overload in the intracellular space (Li B. et al., 2024). These findings indicate that inflammatory cytokines can undermine cellular resistance to cuproptosis.

Two-thirds of copper in the human body is stored in bones, contributing to bone metabolism and development (Han et al., 2024). Previous studies have shown that abnormal distribution of copper ions can be observed in various orthopedic diseases. Copper overload decreases repair capacity and induces cellular death, thereby increasing the risk of osteoarthritis (Li B. et al., 2024). In addition to skeletal disorders, copper ion homeostasis imbalance has been implicated in the pathogenesis of neurodegenerative diseases, cardiovascular diseases, and malignancies (Tong et al., 2022). In patients with Parkinson’s disease, reduced glutathione levels in dopaminergic neurons can lead to excessive intracellular copper accumulation, which disrupts Fe-S cluster proteins and downregulates Fe-S cluster proteins. These alterations mediate cytotoxic stress and cell death, finally leading to neuronal degeneration and even neuronal death (Huang et al., 2024). Vascular injury and inflammation trigger localized copper release, which promotes neointimal hyperplasia and arterial stiffness (Lu et al., 2025). Circulating copper levels are positively correlated with the risk of colorectal cancer, while the presence of pro-tumorigenic cuproptosis biomarkers in breast carcinoma is associated with cancer stemness, contributing to tumor proliferation and metastasis (Chen et al., 2022). Moreover, crystal deposition is linked to cuproptosis. Previous studies have shown that inhalation of silica can induce pulmonary infiltration of immune cells, regulate cuproptosis, and affect the progression of lung fibrosis (Ma R. et al., 2024). How the activation of immune cells in crystal foci of gouty arthritis affects the normal metabolism and repair capacity of chondrocytes remains unclear.

Current therapeutic options for gouty arthritis, such as colchicine, primarily relieve the symptoms and have limited efficacy. We established a screening model by simulating the inflammatory environment in gouty arthritis by co-culturing macrophages and chondrocytes to identify the potential agents that can treat gouty arthritis. We identified cuproptosis as a new mechanism of cartilage injury, specifically inflammation-induced chondrocyte death. We screened 24 natural products. Syringaresinol-4-O-β-d-glucoside (SSG) has shown the best efficacy in reducing macrophage inflammation and suppressing chondrocyte cuproptosis. Further studies showed that SSG inhibited MSU-induced activation of NF-κB and NLRP3 pathways in macrophages. Furthermore, SSG was shown to regulate the expression of sulfur-linked mitochondrial enzymes in the cuproptosis pathway, thereby deactivating the upstream regulator FDX1 in chondrocytes. SSG also alleviated inflammatory pain, protected against cartilage damage, and improved motor dysfunction in mice with acute or chronic gouty arthritis.

## 2 Materials and methods

### 2.1 Reagents and media

Syringaresinol-4-O-β-d-glucoside (C28H36O13; MW 580.58 g/mol; purity ≥98% [TargetMol, Shanghai, China]) was dissolved in dimethyl sulfoxide. Monosodium urate, colchicine and TNF-α were purchased from Merck (Darmstadt, Germany), Dulbecco’s modified Eagle medium (DMEM) and Fetal Bovine Serum (FBS) were provided by Gibco-BRL (Sydney, Australia). Cell co-culture chambers and cell migration chambers were provided by Corning (New York, United States); the cell viability assay kit (CCK-8) was purchased from Dojindo Molecular Technology (Kumamoto, Japan). Phagocytosis Assay Kit (IgG FITC) was provided by Cayman Chemical Company (Michigan, United States). Calcein AM Cell Viability Assay Kit, Ki67 Cell Proliferation Assay Kit, Mitochondrial membrane potential assay kit with JC-1, Senescence β-Galactosidase Staining Kit, Alcian Blue Staining Kit, Crystal Violet Staining Solution were purchased from Beyotime (Shanghai, China). Macrophage M1 polarization marker antibody CD86 was purchased from BD Biosciences (NJ, United States). Primary antibodies targeting NLRP3, GSDMD, Caspase-1, NF-κB, TNF-α, IL-1β, p21, COL2A1, ADAMTS5 and GAPDH were obtained from abcam (Cambridge, United Kingdom). Antibodies against FDX1 and DLAT were provided by Proteintech (Chicago, United States). All other drugs used in this study were purchased from TargetMol. Unless otherwise specified, the drugs were dissolved in DMSO with a working concentration of 100 μM.

### 2.2 Cell cultures

The mouse chondrocyte cell line ATDC5 was purchased from the cell bank of the Chinese Academy of Sciences (Shanghai, China), and the mouse macrophage cell line RAW264.7 was purchased from the Shanghai Fuheng Biological Company (Catalog No. FH0328). Both cells were cultured in DMEM containing 4.5 g/L glucose, 5% FBS and 1% penicillin-streptomycin. We used a co-culture chamber with a pore size of 0.4 μm (Corning, 3450). The upper chamber was seeded with RAW264.7 at a density of 1.5 × 10^5^ cells/well, and the lower chamber was seeded with ATDC5 at a density of 1.5 × 10^5^ cells/well. The second day after the seed plate, the RAW264.7 of the upper chamber was treated with different stimuli. After 1 day of stimulation, the cells were collected. Both cells were cultured in an incubator at 37°C and 5% CO_2_.

### 2.3 Cell viability assay

8.0 × 10^3^ ATDC5 or RAW264.7 cells were seeded in 96-well plates and incubated with different concentrations of SSG (0, 6.25, 12.5, 50, 100, 200, 400, 800 and 1600 μM) in complete DMEM for 24, 48 or 72 h. Then, 100 μL fresh medium containing 10 μL CCK-8 solution was added to each well. The plate was then incubated at 37°C for another 2 h, and the optical density at 450 nm was measured using a microplate reader (Thermo, United States). Cell viability was calculated as the percentage of vector-treated control cells (100%) (Shi X. et al., 2024).

### 2.4 Western blotting

Cells were seeded into six-well plates (1 × 10^5^ cells/well). After undergoing treatment, the cells were washed with PBS and then lysed using RIPA buffer (Sigma-Aldrich) containing protease and phosphatase inhibitors. Proteins were separated by SDS-PAGE, transferred onto PVDF membranes, and probed with specific antibodies. Detection was performed using a chemiluminescent method, and quantification was carried out using ImageJ (Xue et al., 2024).

### 2.5 RNA extraction and real-time quantitative PCR (RT-qPCR) analyses

Total RNA was extracted from the cells using Trizol reagent (Invitrogen) according to the manufacturer’s instructions. One microgram of total RNA was reverse transcribed using the Prime Script RT Kit (TaKaRa Biotechnology). The resulting complementary DNA (cDNA) was then used for RT-qPCR detection with the TB Green Premix Ex Taq Kit (TaKaRa Biotechnology). All reactions were normalized to the expression of the housekeeping gene Gapdh and performed in triplicate. The primers used were listed in Table 1 (Shi X. F. et al., 2024).

**TABLE 1:** Primer sequences.

Gene	Forward primer (5′→3′)	Reverse primer (5′→3′)
*Il-1β*	TGC​CAC​CTT​TTG​ACA​GTG​ATG	TGA​TGT​GCT​GCT​GCG​AGA​TT
*Il-6*	AGA​CAA​AGC​CAG​AGT​CCT​TCA​G	TGC​CGA​GTA​GAT​CTC​AAA​GTG​A
*Tnf-α*	TCA​GCC​GAT​TTG​CTA​TCT​CAT​A	AGT​ACT​TGG​GCA​GAT​TGA​CCT​C
*Ifn-α*	TGA​GCT​ACT​GGT​CAA​CCT​GC	TGT​CCT​TCA​GGC​AGG​AGA​GA
*Ifn-β*	AGA​TCA​ACC​TCA​CCT​ACA​GG	TCA​GAA​ACA​CTG​TCT​GCT​GG
*Ifn-γ*	AGC​GCC​AAG​CAT​TCA​ATG​AG	ATC​TCT​TCC​CCA​CCC​CGA​AT
*Cxcl10*	CCT​GCC​CAC​GTG​TTG​AGA​T	TGA​TGG​TCT​TAG​ATT​CCG​GAT​TC
*Mmp9*	CGA​CGA​CGA​CGA​GTT​GTG​GT	GTT​GCC​GTG​CTC​CGT​GTA​GA
*Mmp13*	GGA​GCC​CTG​ATG​TTT​CCC​AT	GTC​TTC​ATC​GCC​TGG​ACC​ATA
*Col2a1*	GCT​ACA​CTC​AAG​TCA​CTG​AAC​AAC​CA	TCA​ATC​CAG​TAG​TCT​CCG​CTC​TTC​C
*Sox9*	CGT​GGA​CAT​CGG​TGA​ACT​GAG	GGT​GCT​GCT​GAT​GCC​GTA​AC
*Gapdh*	GGC​AAG​TTC​AAC​GGC​ACA​G	CGC​CAG​TAG​ACT​CCA​CGA​CAT

(All primers are of murine origin.).

### 2.6 Immunofluorescence

For immunofluorescence, cells were rinsed with PBS, fixed with 4% paraformaldehyde for 15 min, and then treated with 0.1% (v/v) Triton X-100 for 30 min. After blocking with 3% (v/v) BSA for 1 h, the cells were diluted with DLAT primary antibody (diluted 1: 200; Proteintech, 13426-1-AP) or FDX1 (diluted 1:200; Proteintech, 12592-1-AP) at 4°C overnight. Then the cells were coupled with Alexa Fluor 555 goat anti-rabbit secondary antibody (diluted 1: 300; abcam, Cambridge, United Kingdom) 1 h. Next, the cytoskeleton was stained with a phalloidin-Alexa Fluor 488 solution for 60 min, and the nucleus was stained with DAPI (Beyotime) for 15 min in the dark. Use LSCM to capture images (Li X. et al., 2024).

### 2.7 Transmission electron microscope

The treated chondrocytes were digested, resuspended, and fixed with electron microscope fixative at 4°C for 2–4 h. After dehydration, infiltration, embedding and slicing, the images were observed under a projection microscope (JEOL,1400).

### 2.8 Detection of macrophage phagocytosis ability

RAW 264.7 cells were stimulated with 100 μg/mL MSU and different concentrations of SSG (0, 50, 100 μM). After 24 h, the cells were gently washed with PBS, and the Latex Beads-Rabbit IgG-FITC Complex was diluted with 1:500 dilution in DMEM containing 4.5 g glucose/L, 5% FBS and 1% penicillin-streptomycin. The washed cells were incubated with the above medium for 2 h. Cells were fixed with 4% paraformaldehyde, and images were captured using a microscope (Leica Microsystems, Wetzlar, Germany).

### 2.9 Flow cytometry analysis

RAW264.7 was inoculated in 6-well plates at a density of 3 × 10^5^ cells/well. After MSU stimulation and SSG (0, 50, 100 μM) treatment for 24 h. Then, the cell suspension was prepared, blocked with 0.3% bovine serum albumin (BSA) solution, and reacted with primary antibody (PE CD86; BD Biosciences, United States) for 30 min, washed with fresh 0.3% BSA, and applied to flow cytometry analysis on LSRFortessa flow cytometer (BD Biosciences, United States) to count at least 10,000 events. Finally, the results were analyzed using Flow Jo software (version 10.4, BD Biosciences, United States).

### 2.10 JC-1 assay

RAW264.7 and ATDC5 cells were washed with PBS and then stained with Mitochondrial membrane potential assay kit with JC-1 (Beyotime, C2006). Finally, LSRFortessa flow cytometer (BD Biosciences, United States) was used to analyze at least 10,000 events. Flow Jo software (version 10.4, BD Biosciences, United States) was used for result analysis.

### 2.11 Cell fluorescence counting

The treated ATDC5 was washed with PBS, stained with Calcein AM Cell Viability Assay Kit (C2013S), and imaged with a microscope (Leica Microsystems, Wetzlar, Germany), or digested with trypsin. The cells were analyzed by LSRFortessa flow cytometer (BD Biosciences, United States).

### 2.12 High-density culture

High-Density Culture was used to evaluate the collagen secretion ability of chondrocytes. 1.5 × 10^7^/mL ATDC5 cell suspension was prepared and 10 μL suspension was added to the center of each well in a 24-well culture plate. After 2 h in the cell incubator, 1 mL of DMEM containing 5% FBS and 10 ng/mL insulin transferrin selenium (ITS) (Gibco, Thermo Fisher Scientific, Waltham, MA, United States) was added to each well. The medium was changed every 2 days for 7 days. After fixed in 4% PFA for 10 min, Alcian blue and toluidine blue dyes were added to stain the extracellular matrix in the micromass overnight.

### 2.13 Ki67 cell proliferation assay

After different treatments of ATDC5, the medium was removed and washed with PBS. The expression of Ki67 in cells was detected by Ki67 Cell Proliferation Assay Kit (IF, Red, Rabbit mAb) (Beyotime, C2301S), and the images were collected by microscope (Leica Microsystems, Wetzlar, Germany).

### 2.14 Senescence-associated β-galactosidase (SA-β-gal) staining

MSU-stimulated macrophages and chondrocytes were co-cultured, and chondrocytes were treated with different concentrations of SSG (0, 50, 100 μM) for 24 h. After washing with PBS once, the chondrocytes were fixed in 4% paraformaldehyde for 15 min and stained with SA-β-gal staining kit (Beyotime, China). Images were taken using a Zeiss microscope.

### 2.15 Transwell migration assay

MSU-stimulated RAW264.7 cells were cultured in serum-free DMEM medium containing SSG (0, 50, 100 μM), seeded in a co-culture upper chamber (Corning, 3422) with a pore size of 8 μm, and the co-culture lower chamber medium was DMEM with 5% serum. After 24 h, the dishes were washed with PBS, the upper cells were carefully removed, 4% paraformaldehyde was fixed, and stained with crystal violet for half an hour. After PBS washing, fluorescence microscopy (Olympus IX83, Japan) was used to observe and collect images (Xu et al., 2025).

### 2.16 Mouse model of acute gout and gouty arthritis

All animal procedures were approved by the Experimental Animal Ethics Committee of the Ninth People’s Hospital Affiliated to Shanghai Jiao Tong University School of Medicine (Ethics No. SH9H-2024-A1376-1). The acute gout model was established by randomly dividing twenty-four 8-week-old male C57BL/6J mice into four treatment groups: sham, MSU, MSU + Colchicine, MSU + SSG. For mice that received MSU injection in the knee joint, we anesthetized them with isoflurane, and injected MSU crystals (0.5 mg) suspended in 20 μL endotoxin-free PBS into the knee joint cavity with an insulin needle. At the same time, colchicine (1 mg/kg), SSG (1 mg/kg) or Vehicle were injected intraperitoneally. After 24 h of behavioral testing, the mice were sacrificed, and bilateral knee joints were collected and fixed in 4% paraformaldehyde. Twenty-four 8-week-old male C57BL/6J mice were randomly divided into four treatment groups: sham, MSU, MSU + Colchicine, MSU + SSG. The crystals were injected into the knee joint in the same way every 3 days and administered intraperitoneally at the same time. The behavioral data was recorded at the same time point. 10 weeks later, the mice were sacrificed, and bilateral knee joints were collected and fixed in 4% paraformaldehyde.

### 2.17 Behavioral assessment

In this study, the behavior of mice was analyzed by Von Frey test, hot plate test and gait analysis. To measure the mechanical allodynia in mice, we performed a Von Frey test on each animal: using a flexible nylon Von Frey filament and the “up-down method”, the 50% mechanical paw withdrawal threshold (g) of each mouse was recorded. Hot plate test was used to evaluate the behavioral response of mice to noxious heat. We placed the mice on a Hot plate with a surface of 55°C and began timing until a rapid withdrawal of the soles was observed, which was defined as latency (s). 20 s were set as the cutoff time to protect the paws of mice. For the accuracy of the results, the measurement was performed 3 times every 10 min, and the results were average. All gait analysis is based on the CatWalk runway; the week before the experiment, the animals were put on the CatWalk track for training every day. Effective data were recorded at least once for each animal, with an average of six animals in each group. If the animal movement stops or turns back during the experiment, the record is discarded. The total standing time, average pace, Duty cycle and mean intensity of each paw were collected and analyzed by blind method.

### 2.18 Histological staining and histomorphological analysis

The knee joint fixed in 4% paraformaldehyde was decalcified in 10% (w/v) EDTA (pH 7.4) for about 4 weeks and then embedded in paraffin. Subsequently, Hematoxylin and Eosin Staining (H&E), Safranin-O-Fast Green Staining and immunohistochemical techniques were used for staining, and images were captured using a microscope (Leica Microsystems, Wetzlar, Germany).

### 2.19 Statistical analysis

The data were collected from three independent experiments and presented as mean ± standard deviation (SD). GraphPad Prism version 8.0 software (GraphPad Software, San Diego, CA, United States) was used to performed one-way analysis of variance (ANOVA) or the Student’s t-test. Statistical significance was set at p < 0.05.

## 3 Results

### 3.1 Inflammatory macrophages induced cuproptosis in chondrocytes

**FIGURE 1:**
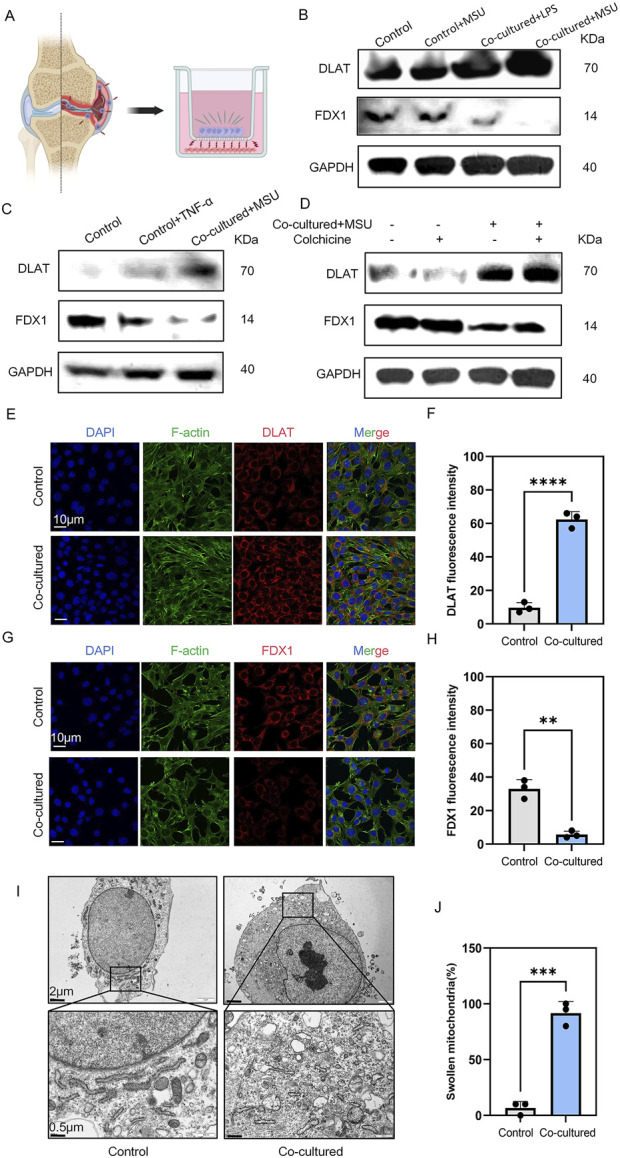
MSU-stimulated RAW264.7 co-cultured with ATDC5 resulted in ATDC5 cuproptosis. **(A)** co-culture strategy of chondrocytes and macrophages. **(B–D)** Western blot detection of DLAT and FDX1 protein expression. **(E)** Representative images of DLAT immunofluorescence. **(F)** Statistics of fluorescence intensity of DLAT, respectively (n = 3). **(G)** Representative images of FDX1 immunofluorescence. **(H)** Statistics of fluorescence intensity of FDX1, respectively (n = 3). **(I)** Representative scanning electron microscope images of chondrocytes. **(J)** Statistics of the proportion of swollen mitochondria in chondrocytes, respectively (n = 3). The bar graph represents the mean ± SD. *p < 0.05, **p < 0.01, ***p < 0.001 compared to the control group.

MSU, macrophages, and chondrocytes are key players in the pathogenesis of gouty arthritis. We employed a co-culture system to mimic gouty arthritis *in vitro* and unravel the relationship between inflammatory macrophages and chondrocytes (Figure 1A). After co-culturing with RAW264.7 macrophages stimulated with LPS and MSU, ATDC5 chondrocytes exhibited signs of cuproptosis, such DLAT upregulation and FDX1 downregulation, compared to ATDC5 chondrocytes stimulated solely with LPS and MSU (Figure 1B). Subsequent stimulation of ATDC5 chondrocytes with the inflammatory cytokine TNF-α similarly enhanced cuproptosis, indicating that the inflammatory cytokines released by macrophages, such as TNF-α, can promote chondrocyte cuproptosis (Figure 1C). Notably, treatment with colchicine could not prevent MSU-induced chondrocyte cuproptosis (Figure 1D). Immunofluorescence analysis confirmed enhanced DLAT expression and decreased FDX1 expression (Figures 1E–H). Furthermore, electron microscopy revealed mitochondrial damage in chondrocytes (Figures 1I,J). Collectively, macrophage-mediated inflammation and chondrocytic cuproptosis are involved in the development of cartilage damage in gouty arthritis.

### 3.2 Identification of SSG as an effective compound that can attenuate both macrophage-mediated inflammation and chondrocyte cuproptosis

**FIGURE 2:**
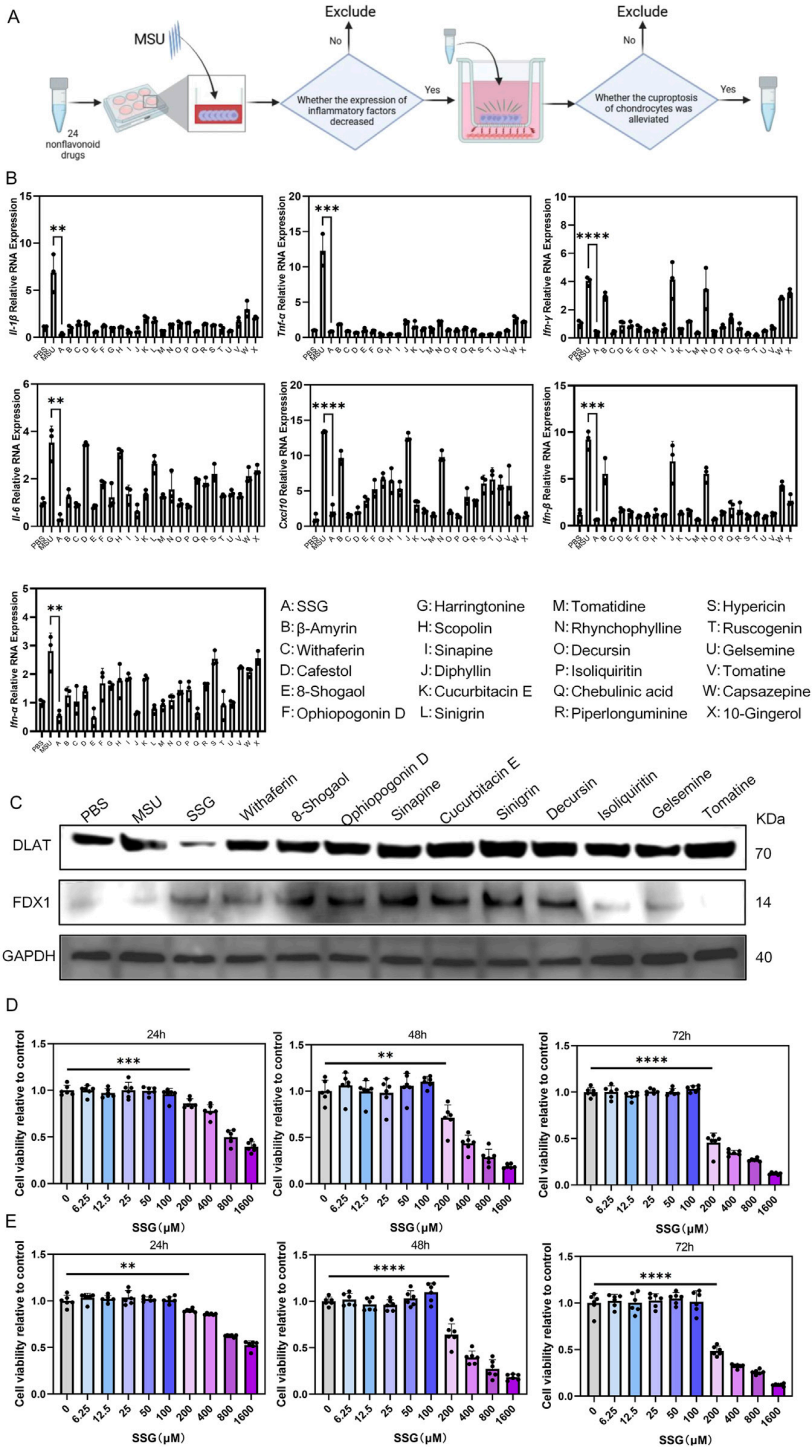
Drug screening *in vitro* confirmed that SSG could inhibit inflammation and resist cuproptosis of chondrocytes. **(A)** Flowchart of Drug Screening. **(B)** The gene expression levels of *Il-1β*, *Il-6*, *Tnf-α*, *Ifn-α*, *Ifn-β*, *Ifn-γ* and *Cxcl10* were evaluated using RT-qPCR (n = 3). **(C)** Western blot detection of DLAT and FDX1 protein expression. **(D)** The CCK8 assay was used to assess the viability of OSC on RAW264.7 at specified time points (n = 6). **(E)** The CCK8 assay was used to assess the viability of OSC on ATDC5 at specified time points (n = 6). The bar graph represents the mean ± SD. *p < 0.05, **p < 0.01, ***p < 0.001 compared to the control group.

We screened 24 non-flavonoid compounds with anti-inflammatory and antioxidant properties (Figure 2A). Initially, all compounds were screened by evaluating their effects on reducing the expression of inflammatory cytokines in macrophages after stimulation with MSU. We measured the expression of seven cytokines, including *Il-1β*, *Il-6*, *Tnf-α*, *Ifn-α*, *Ifn-β*, *Ifn-γ*, and *Cxcl10* (Figure 2B). We selected 11 compounds with better anti-inflammatory effects for further experiments. These 11 candidate compounds were subsequently introduced into a co-culture system with macrophages and chondrocytes (Figure 2C). Among them, SSG exhibited the strongest protective effect against chondrocyte cuproptosis. CCK-8 assays were conducted at 24, 48, and 72 h in both chondrocytes and macrophages, which showed that SSG concentrations up to 100 μM exhibited no cytotoxic effects (Figures 2D,E).

### 3.3 SSG inhibited MSU-induced macrophage-mediated inflammation

**FIGURE 3:**
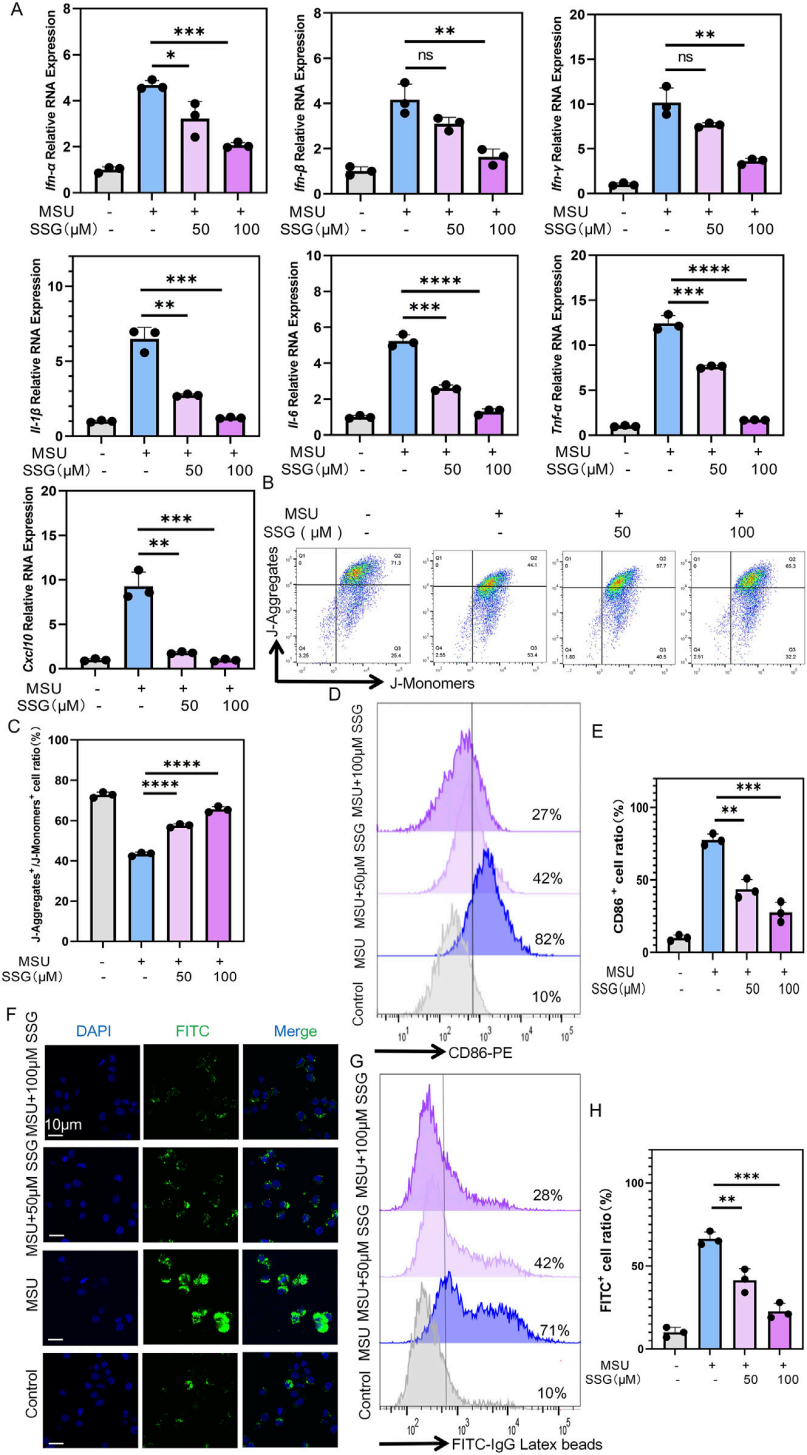
SSG inhibited the inflammatory phenotype of macrophages stimulated by MSU in a dose-dependent manner. **(A)** The gene expression levels of *Il-1β*, *Il-6*, *Tnf-α*, *Ifn-α*, *Ifn-β*, *Ifn-γ* and *Cxcl10* were evaluated using RT-qPCR (n = 3). **(B)** JC-1 assay is used to detect changes in mitochondrial membrane potential in macrophages. **(C)** The ratio of J-Aggregate^+^/J-Monomer^+^ cells in panel B (n = 3). **(D)** Flow cytometry of CD86 marker in macrophages. **(E)** Quantifying CD86 labeled positive cells shown in panel D (n = 3). **(F)** Representative images of macrophages phagocytosing FITC-labeled particles. **(G)** Flow cytometry of FITC-labeled particles phagocytosed by macrophages. **(H)** Quantifying FITC labeled positive cells shown in panel G (n = 3). The bar graph represents the mean ± SD. *p < 0.05, **p < 0.01, ***p < 0.001 compared to the control group.

MSU stimulates the release of inflammatory cytokines and chemokines by macrophages (Rock et al., 2013). We quantitatively measured the anti-inflammatory effects of SSG using the aforementioned seven inflammatory indicators and qPCR revealed a dose-dependent decrease in all indicators (Figure 3A). For example, *Il-1β* levels increased seven folds in the positive control group. However, SSG supplementation at 50 μM led to a 3-fold increase in *Il-1β* levels. SSG supplementation at 100 μM completely inhibited the expression of *Il-1β*. Additionally, SSG dose-dependently inhibited the M1 polarization of macrophages, impaired their phagocytic ability, and protected against mitochondrial dysfunction (Figures 3B–H). We added SSG and commonly used gout medications, including allopurinol, febuxostat, probenecid, canakinumab, and colchicine, at appropriate concentrations to MSU-stimulated RAW264.7 cell culture to validate the anti-inflammatory effects of SSG. Next, we employed qPCR to measure the inhibitory effects of these drugs on the expression of inflammatory factors. Although the inhibitory effect of SSG on *Il-1β*, *Ifn-γ*, and *Cxcl10* was weaker than that of canakinumab, SSG more effectively promoted the release of anti-inflammatory factors compared to classic treatments of gout (Supplementary Figure S1A). These findings suggest that SSG can alleviate the inflammatory phenotype of macrophages after exposure to MSU crystals.

### 3.4 SSG inhibited the NLRP3 and NF-κB pathways in macrophages

**FIGURE 4:**
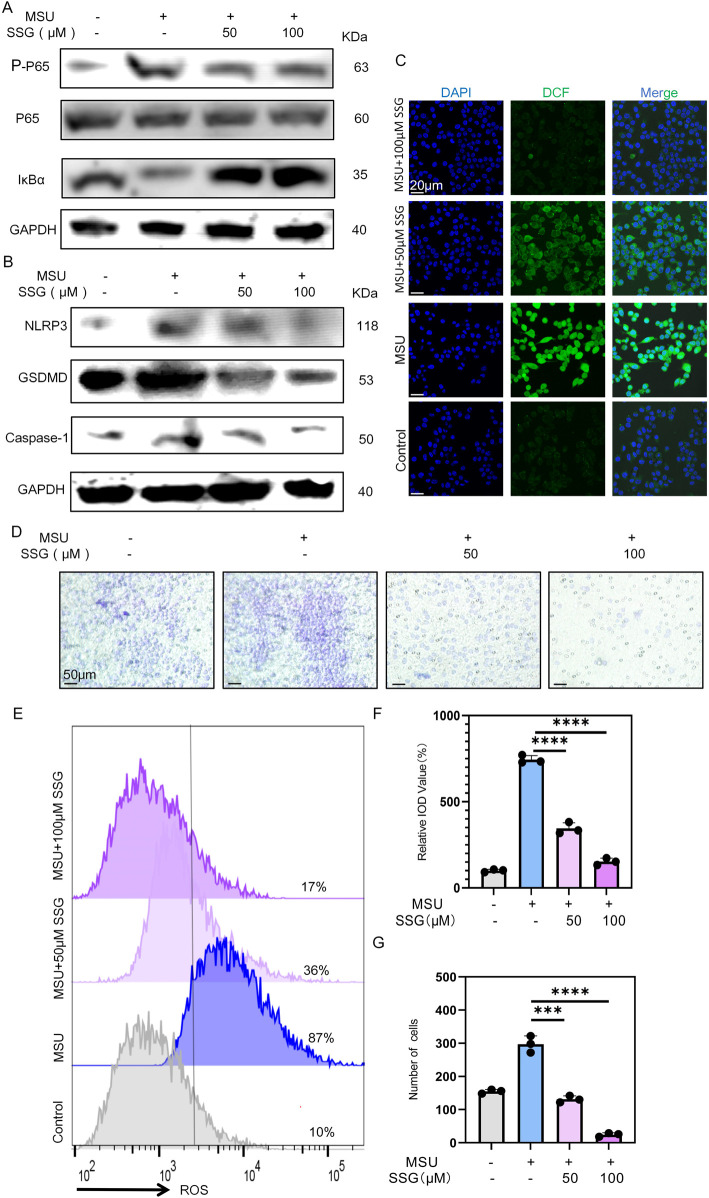
SSG inhibits the activation of NF-κB and NLRP3 pathways in MSU-stimulated macrophages and reduces the pro-inflammatory phenotype of macrophages. **(A)** The protein expression levels of IκBα, p65 and p-p65 were assessed by Western blot. **(B)** Western blot detection of GSDMD, Caspase1, NLRP3 protein expression. **(C)** Representative images of ROS fluorescence. **(D)** Use cell migration experiments to analyze macrophage chemotactic ability. **(E)** Flow cytometry of ROS marker in macrophages. **(F)** Quantifying relative IOD value (%) shown in panel C (n = 3). **(G)** Statistics of the number of migrating macrophages (n = 3). The bar graph represents the mean ± SD. *p < 0.05, **p < 0.01, ***p < 0.001 compared to the control group.

Macrophages recognize MSU crystals through TLR receptors on their cell membrane, which activates the downstream NF-κB and NLRP3 inflammasome (Li et al., 2023). After MSU stimulation, we observed increased phosphorylation of p65 and degradation of IκBα. Interestingly, SSG supplementation dose-dependently inhibited the NF-κB pathway (Figure 4A). Thereafter, we measured the levels of GSDMD, Caspase-1, and NLRP3. MSU stimulation increased the expression of these proteins, suggesting the activation of the NLRP3 pathway. SSG dose-dependently reversed the increase in their expression (Figure 4B). ROS level was upregulated in the positive control group, with a relative IOD value up to 750% that of the sham group. After SSG supplementation at 50 μM, the relative IOD value decreased by 400%. SSG supplementation at 100 μM decreased the relative IOD value to nearly that of the sham group, which was consistent with the results of JC-1 experiments (Figures 4C,E,F; Supplementary Figure S2A). Transwell migration assay revealed that SSG downregulated macrophage chemotaxis (Figures 4D,G). These findings indicated that SSG exerts anti-inflammatory effects by inhibiting the NF-κB and NLRP3 pathways in MSU-stimulated macrophages.

### 3.5 SSG alleviated chondrocyte senescence and protected against extracellular matrix degradation

**FIGURE 5:**
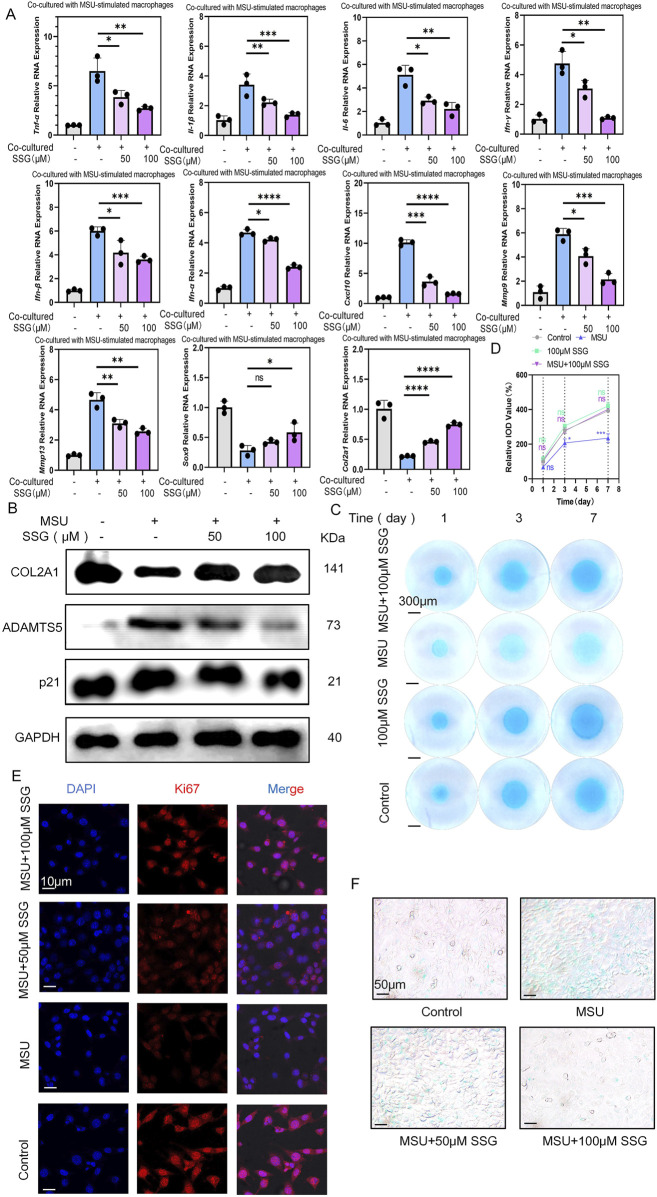
SSG alleviated the senescence and extracellular matrix degradation of ATDC5 co-cultured with MSU-stimulated RAW264.7 cells. **(A)** The gene expression levels of *Il-1β*, *Il-6*, *Tnf-α*, *Ifn-α*, *Ifn-β*, *Ifn-γ*, *Cxcl10*, *Mmp-3*, *Mmp13*, *Sox9* and *Col2a1* were evaluated using RT-qPCR (n = 3). **(B)** The protein expression of COL2A1, p21 and ADAMTS5 were assessed by Western blot. **(C)** Detection of extracellular matrix production capacity in high-density chondrocyte culture experiments at specified time points. **(D)** Quantifying relative IOD value (%) shown in panel C (n = 3). **(E)** Representative images of Ki67 expression in ATDC5. **(F)** β-gal staining experiment for detecting the degree of chondrocyte senescence. The bar graph represents the mean ± SD. *p < 0.05, **p < 0.01, ***p < 0.001 compared to the control group.

The inflammatory environment can induce chondrocyte senescence and extracellular matrix degradation (Tuckermann and Adams, 2021). In co-cultures of macrophages stimulated with MSU, we treated the cells with SSG and observed a dose-dependent decrease in the expression of seven inflammatory cytokines in chondrocytes. Additionally, the genes *matrix metallopeptidase 3* (*Mmp3*) and *matrix metallopeptidase 13* (*Mmp13*), which are associated with chondrolysis phenotypes, were downregulated, whereas the chondrocyte transcription factor *Sox9* and the extracellular matrix production gene *collagen type II alpha 1* (*Col2a1*) were upregulated (Figure 5A). Compared to classic gout medications, SSG was as effective as canakinumab in inhibiting inflammation in chondrocytes after co-culture. SSG was superior to colchicine in downregulating the expression levels of five inflammatory factors, including *Tnf-α*, *Ifn-γ*, *Ifn-α*, *Ifn-β*, and *Cxcl10*. This finding is consistent with the experimental results obtained from macrophages (Supplementary Figure S1B). Western blotting indicated a significant increase in the levels of chondrolysis-associated protein ADAMTS5 after co-culturing chondrocytes with MSU-stimulated macrophages, but the expression of these proteins was downregulated after adding SSG. SSG also decreased the expression of the chondrocyte senescence gene p21 and increased the expression of the cartilage matrix protein COL2A1 (Figure 5B). Subsequently, high-density culture experiments demonstrated the protective effect of the drug on chondrocyte extracellular matrix (Figures 5C,D). β-galactosidase staining experiment highlighted the ability of the drug to prevent chondrocyte senescence, and Ki67 staining indicated that SSG promoted chondrocyte proliferation in the inflammatory environment (Figures 5E,F; Supplementary Figures S2B,C). Our data suggest that SSG alleviated chondrocyte senescence after inflammatory stimulation and promoted chondrocyte proliferation and extracellular matrix synthesis.

### 3.6 SSG rescued chondrocyte cuproptosis

**FIGURE 6:**
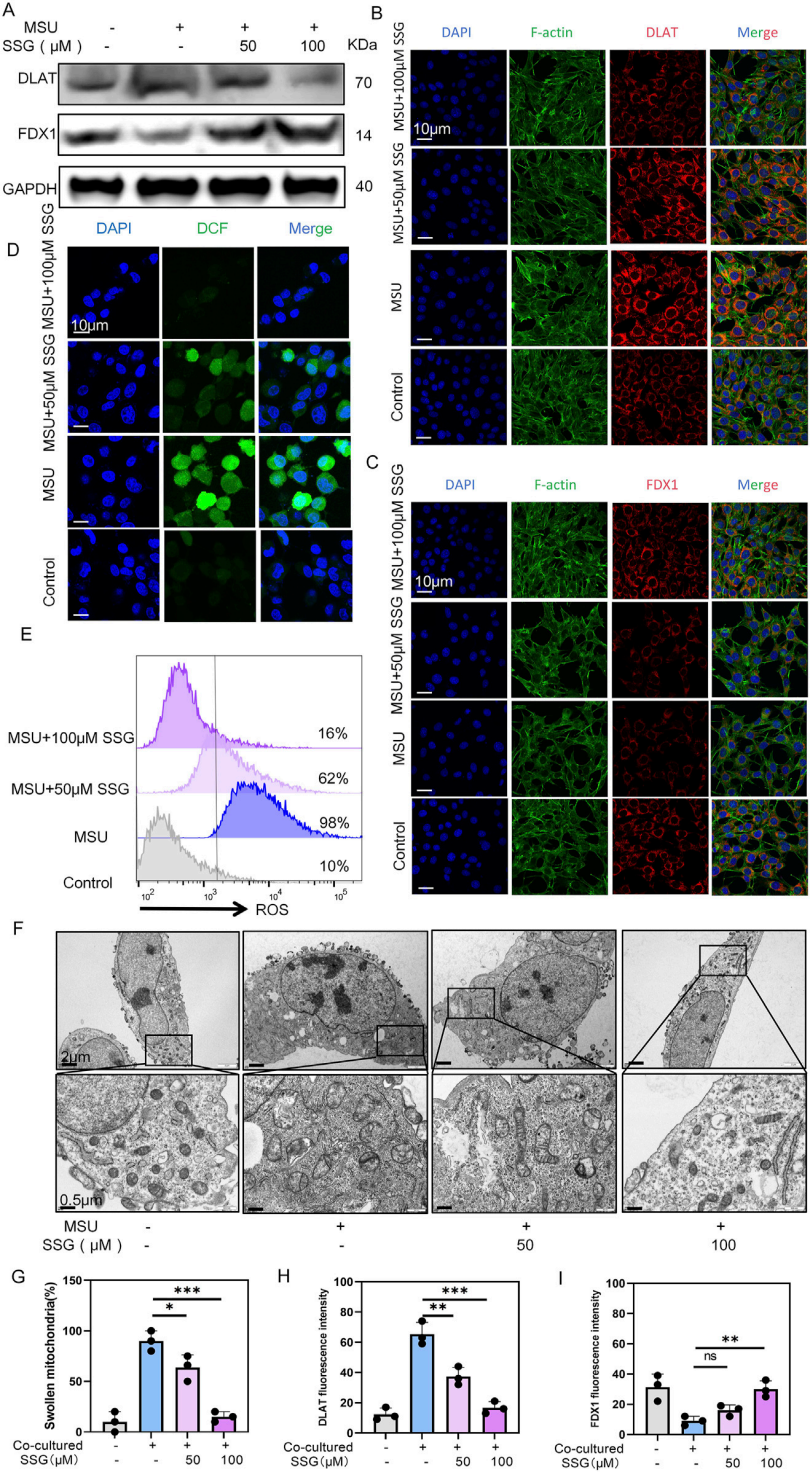
SSG reduces the cuproptosis of chondrocytes caused by MSU-induced release of inflammatory factors from macrophages. **(A)** Western blot detection of DLAT and FDX1 protein expression. **(B)** Representative images of DLAT immunofluorescence. **(C)** Representative images of FDX1 immunofluorescence. **(D)** Representative images of ROS fluorescence in chondrocytes. **(E)** Flow cytometry of ROS marker in chondrocytes. **(F)** Representative scanning electron microscope images of chondrocytes. **(G)** Statistics of the proportion of swollen mitochondria in chondrocytes, respectively (n = 3). **(H,I)** Statistics of fluorescence intensity of DLAT and FDX1, respectively (n = 3). The bar graph represents the mean ± SD. *p < 0.05, **p < 0.01, ***p < 0.001 compared to the control group.

Since cuproptosis is upregulated in gouty arthritis, we investigated whether SSG can mitigate the cuproptosis of chondrocytes. Therefore, we measured the effects of drugs on two key proteins DLAT and FDX1 in cuproptosis (Zhang X. et al., 2024). Both Western blot and immunofluorescence revealed a dose-dependent decrease in FDX1 expression and an increase in DLAT expression (Figures 6A–C,H,I). Chondrocyte viability staining indicated that approximately 60% of the cells in the co-culture group were dead. However, SSG supplementation at 50 μM decreased this percentage to 30%. SSG supplementation at 100 μM further decreased this percentage to 10% (Supplementary Figure S2F–H). Additionally, JC-1 flow cytometry and ROS detection demonstrated that SSG alleviated oxidative stress in chondrocytes and reduced chondrocyte death (Figures 6D,E; Supplementary Figure S2D,E,I,J). Scanning electron microscopy indicated that the mitochondrial membrane damage of chondrocytes decreased after treatment with SSG, and the length-width ratio of mitochondria recovered (Figures 6F,G). SSG not only maintained chondrocyte function but also inhibited chondrocyte cuproptosis in the inflammatory environment associated with gout.

### 3.7 SSG reduced synovial inflammation and cartilage damage in the mice model of acute gout

**FIGURE 7:**
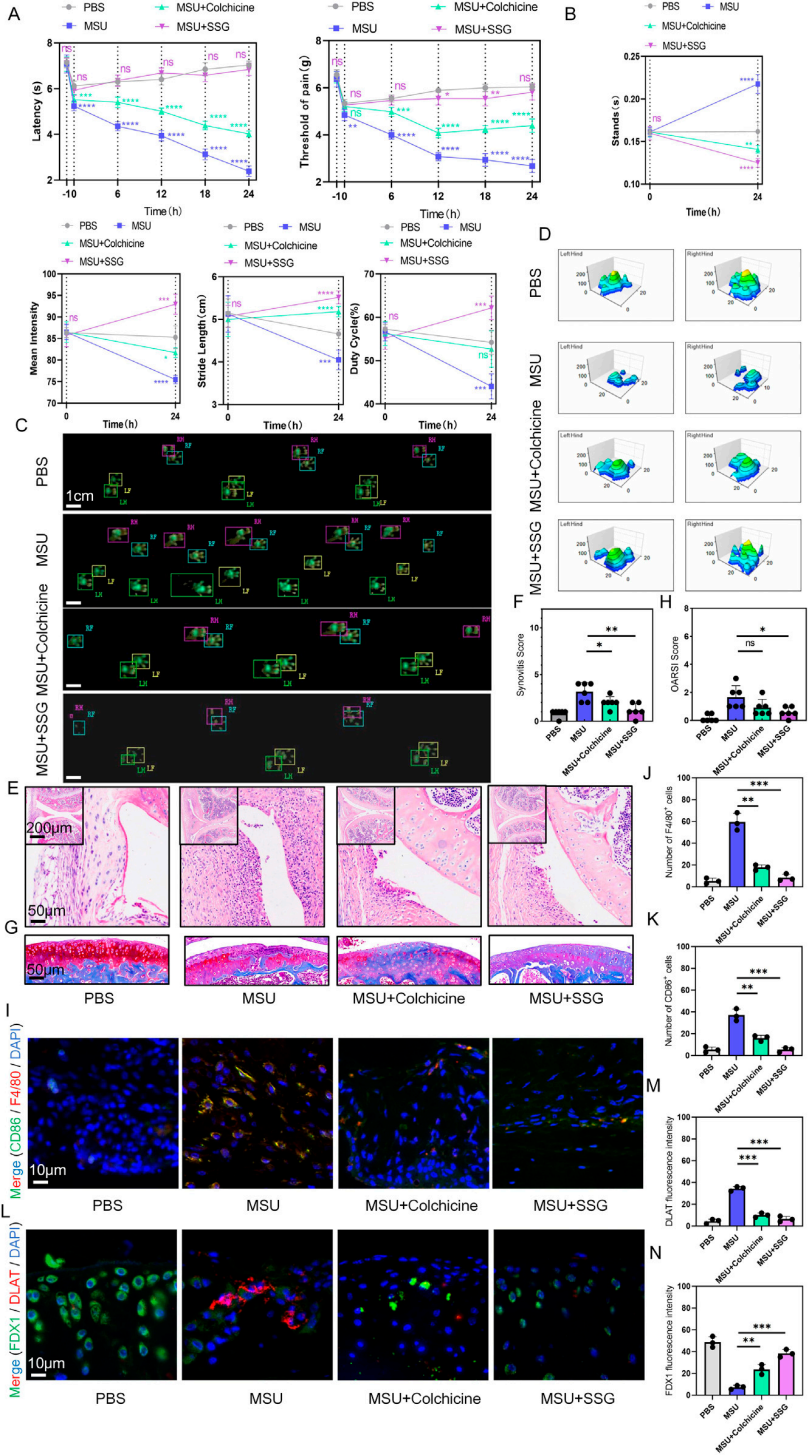
SSG reduced movement disorders, synovial inflammatory cell infiltration and cartilage damage in acute gout mice. **(A)** Reflexive mechanical and thermal pain-related responses within 24 h after injection of MSU crystals (0.8 mg/site) into the knee joints of mice subjected to different treatments (n = 6). **(B)** Changes in the four indices of stands, mean intensity, stride length, and duty cycle in gait analysis of mice before and after the experiment (n = 6). **(C)** Footprint impression diagram for mouse gait analysis. **(D)** Schematic diagram of the maximum pressure on the left and right hind limbs of mice. **(E,F)** Representative H&E stained images of mouse synovium and statistical analysis of synovial swelling index (n = 6). **(G,H)** Representative images of mouse joint sections stained with Safranin-O-Fast Green and statistical analysis of articular cartilage damage index (n = 6). **(I–K)** Representative images of double immunohistochemical staining for F4/80 and CD86 in mouse synovium and statistical analysis of the number of cells positive for both markers (n = 3). **(L–N)** Representative images of double immunohistochemical staining for DLAT and FDX1 in mouse articular cartilage and statistical analysis of the fluorescence intensity for both markers (n = 3). The bar graph represents the mean ± SD. *p < 0.05, **p < 0.01, ***p < 0.001 compared to the control group.

The typical symptoms of acute gout include severe pain and joint swelling. To mimic acute gouty arthritis, we injected MSU crystals into the knee joints of mice and conducted 24-h pain assessments and gait analysis before establishing the model and after treatment. Surprisingly, compared to colchicine, SSG demonstrated excellent analgesic effects in the short term and significantly improved the motor function of mice, allowing them to move with larger strides, faster speeds, and greater plantar pressure (Figures 7A–D). Acute gout led to synovial swelling and mild cartilage damage, and SSG effectively reduced synovial inflammation and preserved articular cartilage (Figures 7E–H). Immunohistochemical staining of synovial tissue for F4/80 and CD86 revealed that SSG reduced the infiltration and activation of local macrophages (Figures 7I–K; Supplementary Figure S3A). Additionally, immunohistochemical staining of cartilage for DLAT and FDX1 showed that SSG significantly inhibited cuproptosis in the cartilage in the short term (Figures 7L–N; Supplementary Figure S3B).

### 3.8 SSG alleviated inflammation and cartilage damage in the mice model of chronic gouty arthritis

**FIGURE 8:**
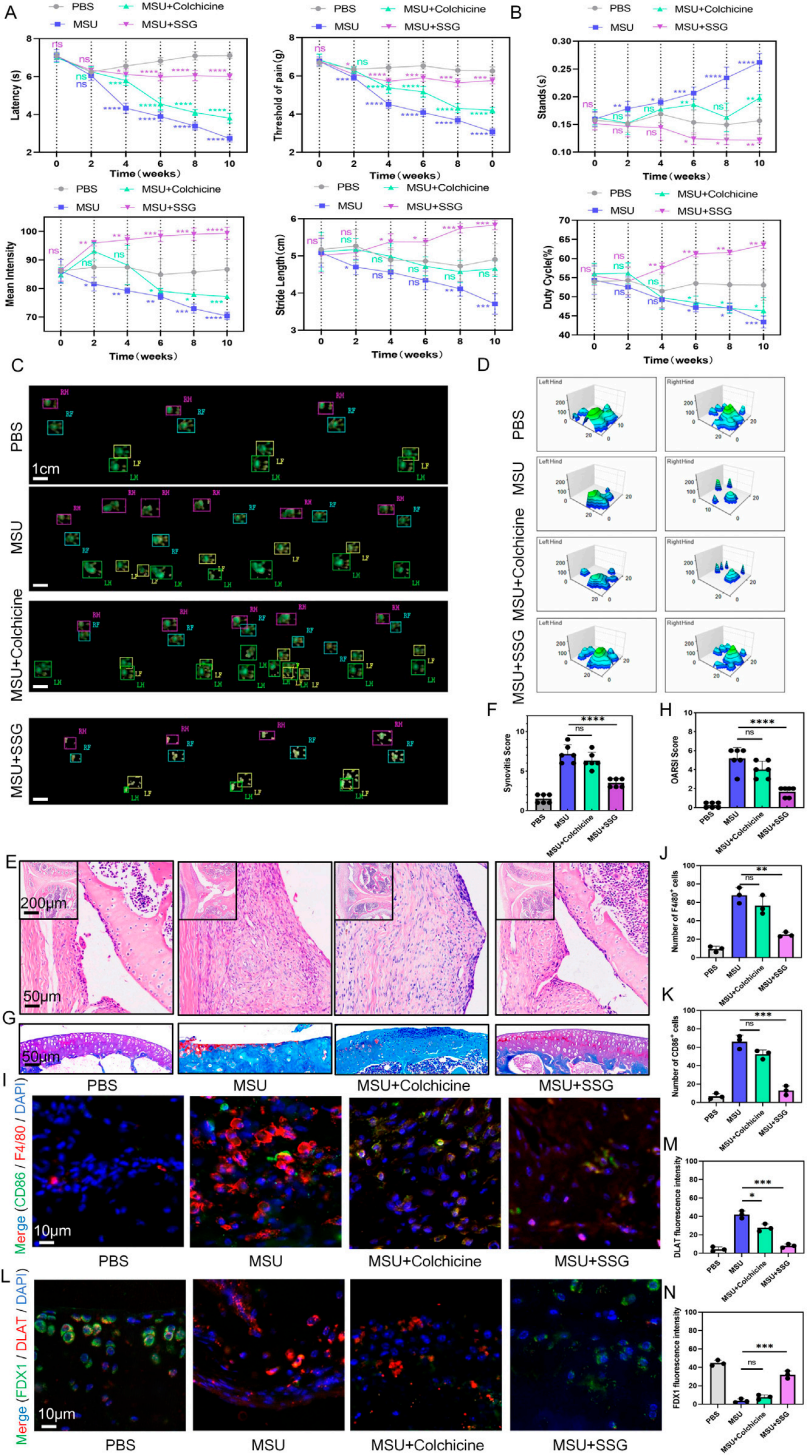
SSG reduced dyskinesia, synovial inflammatory cell infiltration, and cuproptosis of chondrocyte in gouty arthritis mice. **(A)** Administer repeated knee joint injections of MSU and drug treatment to mice over a period of 10 weeks, and record reflexive mechanical and thermal pain-related responses every 2 weeks (n = 6). **(B)** Perform gait analysis on mice every 2 weeks over a 10-week period, and record and analyze the changes in the four indices: stands, mean intensity, stride length, and duty cycle. **(C)** Footprint impression diagram from gait analysis of mice after 10 weeks. **(D)** Schematic diagram of the maximum pressure on the left and right hind limbs of mice after 10 weeks. **(E,F)** Representative H&E-stained images of mouse synovium, accompanied by statistical analysis of the synovial swelling index (n = 6). **(G,H)** Representative images of mouse joint sections stained with Safranin-O-Fast Green, along with statistical analysis of the articular cartilage damage index (n = 6). **(I–K)** Representative images of double immunohistochemical staining for F4/80 and CD86 in mouse synovium, including statistical analysis of the number of cells positive for both markers (n = 3). **(L–N)** Representative images of double immunohistochemical staining for DLAT and FDX1 in mouse articular cartilage, with statistical analysis of the fluorescence intensity for both markers provided (n = 3). The bar graph represents the mean ± SD. *p < 0.05, **p < 0.01, ***p < 0.001 compared to the control group.

Repeated episodes of gout that are resistant to treatment can lead to osteoarthritis and joint deformities. We administered MSU and treatments to mice for 10 weeks, and behavioral assessments were conducted every 2 weeks. We found that the conventional drug colchicine exhibited analgesic effects and partly preserved motor function in the first 4 weeks, whereas SSG exhibited sustained therapeutic efficacy throughout the 10-week study period (Figures 8A–D). H&E staining of the synovium and Safranin-O-Fast Green staining of the joints indicated that SSG effectively alleviated synovial swelling and joint damage caused by long-term gout, while colchicine did not achieve such effects (Figures 8E–H). Immunohistochemical staining of the synovium and cartilage showed that SSG suppressed synovial inflammation and macrophage infiltration, preserved cartilage morphology, and mitigated cuproptosis, consistent with the results of our previous cellular experiments (Figures 8I–N; Supplementary Figures S4A,B).

## 4 Discussion

The incidence of gout has increased globally. Recent studies have indicated that tophus, commonly found surrounding the lesion of gouty arthritis, is formed by MSU crystals and chronic granulomatous tissue, which is encapsulated by fibers and blood vessels (Peng et al., 2023). Chronic gouty arthritis recurs due to persistent inflammation induced by pro-inflammatory and anti-inflammatory cytokines in the surrounding fibrovascular tissue, leading to cartilage damage (Stamp and Dalbeth, 2019). Our study indicated that inflammatory factors released by macrophages can promote cuproptosis in chondrocytes, elucidating the mechanisms underlying cartilage damage in gouty arthritis.

Recent studies have indicated that cuproptosis is commonly observed in Menkes disease, Wilson disease, neurodegenerative diseases, and cardiovascular diseases. There are already therapeutic approaches promoting cuproptosis in cancer (Liu W. Q. et al., 2024; Zhang C. et al., 2024; Zhao et al., 2022; Lutsenko et al., 2025). We initially hypothesized that the pathogenesis of gout is accompanied by chondrocyte senescence and abnormal copper ion metabolism in the intracellular space, leading to cuproptosis and osteoarthritis. Our study indicated that in a co-culture model simulating the microenvironment of gouty joints, inflammatory factors released by macrophages can significantly induce cuproptosis in chondrocytes, characterized by decreased FDX1 expression and increased DLAT expression. This phenomenon is similar to that observed in chondrocytes induced by TNF-α. However, treatment with colchicine did not alleviate cuproptosis in chondrocytes.

We mechanistically inhibited inflammatory factors expression in macrophages and alleviated cuproptosis in chondrocytes. From 24 non-flavonoid traditional Chinese medicines, we identified SSG as the most effective therapeutic agent. SSG reduced macrophage immune responses by inhibiting the NF-κB and NLRP3 pathways. It also suppressed pro-inflammatory phenotypes, including M1 polarization, phagocytosis, mitochondrial damage, and ROS production. In chondrocytes, we demonstrated that SSG can inhibit inflammatory pathways in chondrocytes, prevent the degradation of the extracellular matrix, promote chondrocyte proliferation, delay aging, and significantly reduce cuproptosis. Previous studies have shown that SSG possessed vigorous analgesic effects (Lee et al., 2022). In mouse models of both acute and chronic gout, SSG exhibited both short-term and long-term analgesic effects, with excellent effects on the recovery of mouse motor function. It also effectively alleviated synovitis and protected the cartilage. Colchicine can only be used for short-term treatment, but its long-term efficacy remains unsatisfactory. In immunohistochemical staining of the tissues, SSG significantly reduced macrophage infiltration and M1 polarization, and alleviated cuproptosis in chondrocytes. In addition, SSG was shown to mitigate diabetes and anxiety (Zhai and Wang, 2018). In the mice models of ulcerative colitis, SSG protected intestinal epithelium by inhibiting the PI3K-Akt/MAPK/Wnt pathway (Liu Y. et al., 2024). Therefore, further studies using DEL technology are needed to screen cuproptosis key proteins in gouty arthritis, aiming to identify more specific and potent drug candidates (Ma P. et al., 2024).

Colchicine exerts its anti-inflammatory effects by binding to tubulin heterodimers, thereby interfering with microtubule formation and subsequently inhibiting neutrophil chemotaxis, inflammasome assembly, and interleukin expression (Guo et al., 2025). It is clinically used to prevent and treat acute episodes of gout and familial Mediterranean fever. Colchicine has also shown promising therapeutic effects in pericarditis, chronic coronary artery disease, atrial fibrillation, and thrombosis, and is applied in clinical practice (Imazio and Nidorf, 2021). However, its clinical application is limited by its interaction with antifungal drugs, gastrointestinal side effects, and contraindications in patients with hepatic or renal insufficiency, highlighting the need for safer and more effective anti-inflammatory drugs (Nidorf et al., 2024). Our study demonstrated that colchicine cannot inhibit cuproptosis in chondrocytes in gout, and both *in vitro* and *in vivo* experiments showed that SSG is superior to colchicine in suppressing synovial inflammation, providing analgesic effects, protecting the cartilage, and restoring motor function. Therefore, SSG possesses significant therapeutic potential for inflammatory diseases, such as gout.

Our study demonstrated that SSG possesses potent anti-inflammatory effects and maintains chondrocyte function *in vitro* and *in vivo*; however, the validation of its safety and efficacy in clinical settings necessitates future studies. Several critical issues must be addressed to promote the clinical application of this drug. First, it is needed to clarify the conservation of SSG’s protein targets between humans and animals. Second, validating the immunogenic potential of drug metabolites and the parent compound in human cells and in humans is needed before clinical application. Third, both acute and chronic toxicity experiments and safety profiles in humans are necessary before clinical translation. This integrated approach can facilitate the systematic translation of preclinical findings into clinical applications while addressing critical gaps in current knowledge.

Our study had several limitations. First, we did not identify the specific protein targets through which SSG inhibited the NLRP3 inflammasome and NF-κB signaling pathways to suppress the release of inflammatory factors from macrophages. Second, although we demonstrated that inflammatory factors induce cuproptosis in chondrocytes, we did not investigate how these factors alter copper ion influx and efflux in chondrocytes. In the future, we plan to investigate changes in the expression levels of ion channels, such as ATP7A, ATP7B, and CTR1. Finally, the current study was based on murine cells and mouse models, and we did not validate the efficacy and potential toxic side effects of SSG in human cells. Therefore, we will verify these issues using human cells, such as THP-1 and chondrocytes, to determine the optimal administration route and appropriate dosage.

In summary, our study indicated that MSU induced inflammatory factor release by macrophages and impaired chondrocyte copper homeostasis in gout. Our study revealed that SSG can suppress inflammatory cytokine release by macrophages while protecting chondrocytes and decelerating the progression of cuproptosis.

## 5 Conclusion

The key pathogenic factor of gout, MSU crystals, activates various complex innate immune responses in macrophages, leading to synovial swelling and cartilage damage (Cabău et al., 2020). Inhibiting macrophage-mediated inflammation and protecting cartilage are promising strategies for alleviating gouty arthritis (Cleophas et al., 2017). Our study indicated that inflammatory cytokines released by MSU-induced macrophages enhance cuproptosis in chondrocytes, while SSG can inhibit the NF-κB and NLRP3 inflammasome pathways to suppress macrophage-mediated immune responses and maintain normal copper homeostasis in chondrocytes. Furthermore, we demonstrated the potential therapeutic effects of SSG in mice models of both acute and chronic gout.

## Data Availability

The original contributions presented in the study are included in the article/Supplementary Material, further inquiries can be directed to the corresponding authors.
