# Supplementary figures and images for: Suppressing chondrocyte cuproptosis by syringaresinol-4-O-β-d-glucoside alleviates gouty arthritis
Source: Front Pharmacol. 2025 May 9;16:1565422. doi: 10.3389/fphar.2025.1565422 (PMC12099060; doi:10.3389/fphar.2025.1565422)

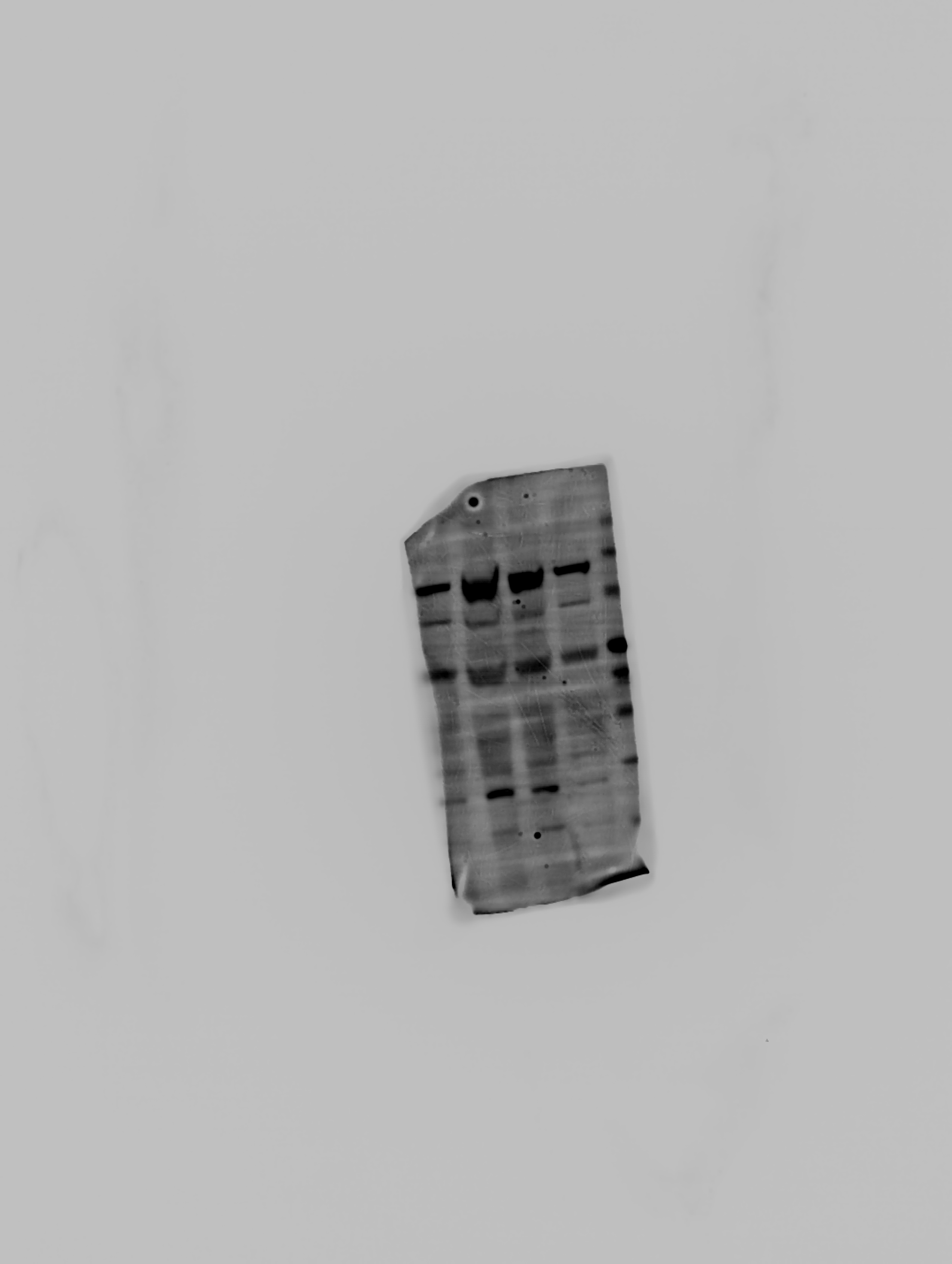

Supplement: Supplementary file 1 [file DataSheet3.zip › Figure4 wb/Figure 4 caspase1 2.tif]

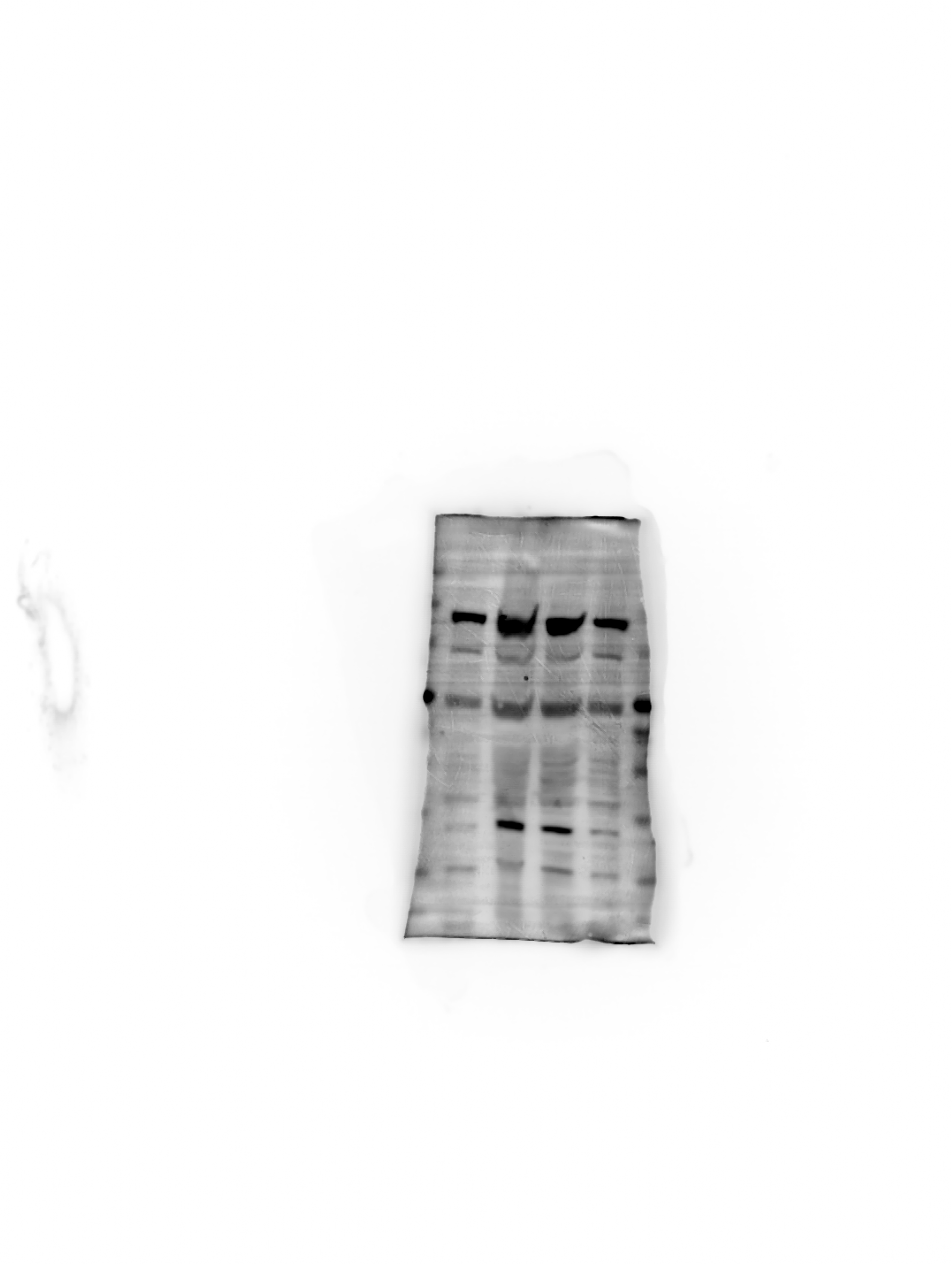

Supplement: Supplementary file 1 [file DataSheet3.zip › Figure4 wb/Figure 4 caspase1 3.tif]

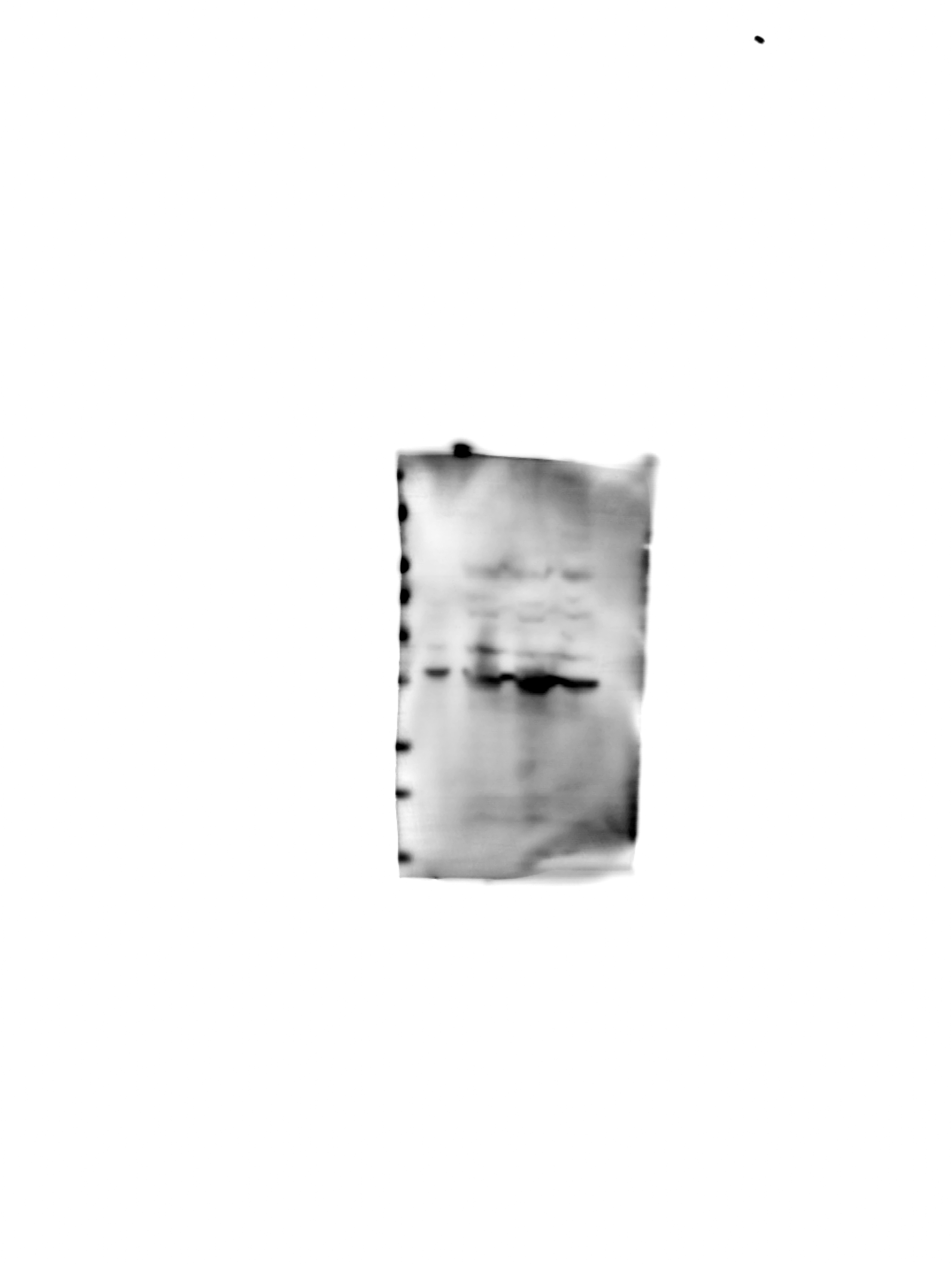

Supplement: Supplementary file 1 [file DataSheet3.zip › Figure4 wb/GSDMD XH 1.tif]

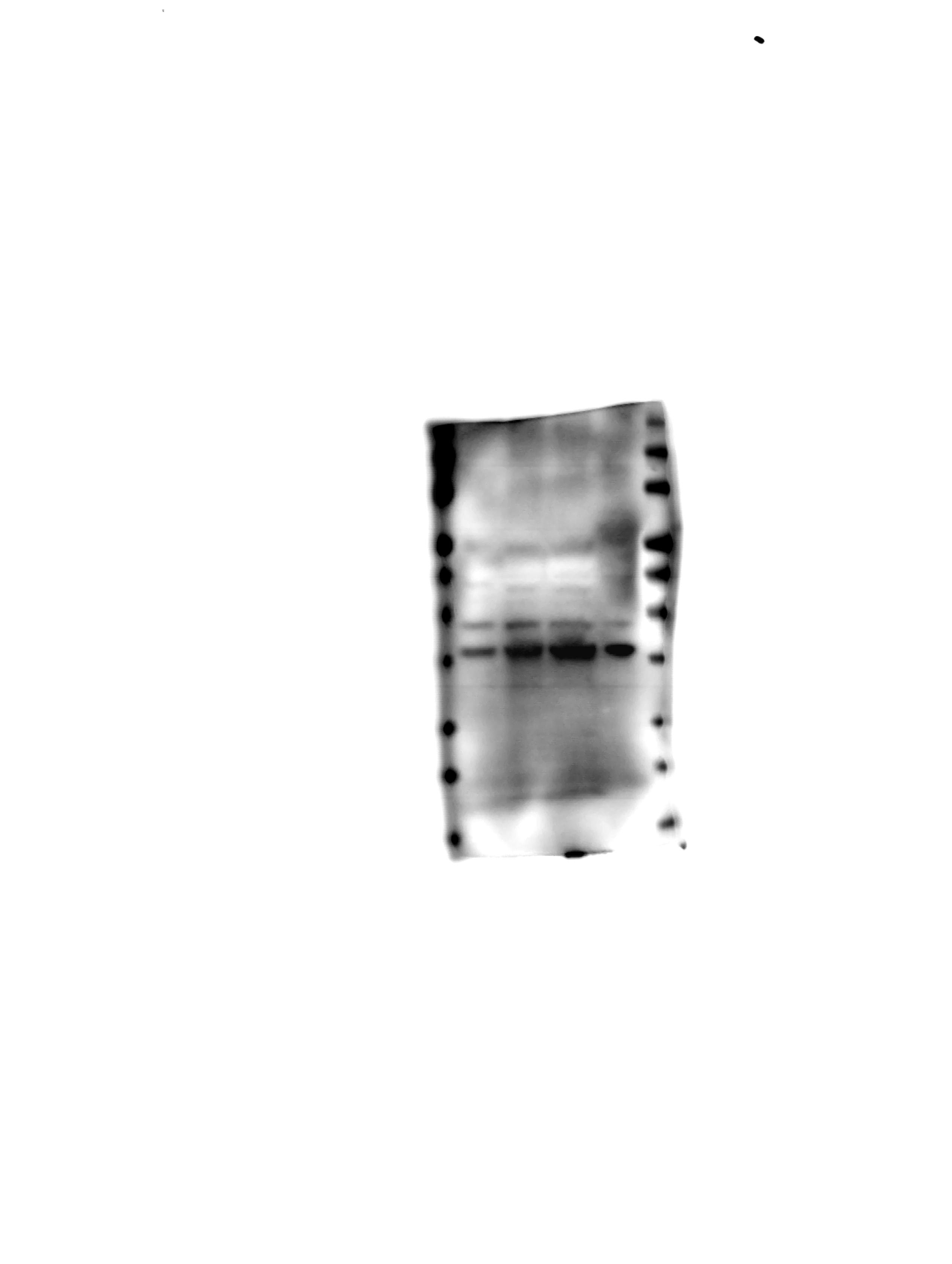

Supplement: Supplementary file 1 [file DataSheet3.zip › Figure4 wb/GSDMD XH 2 1.tif]

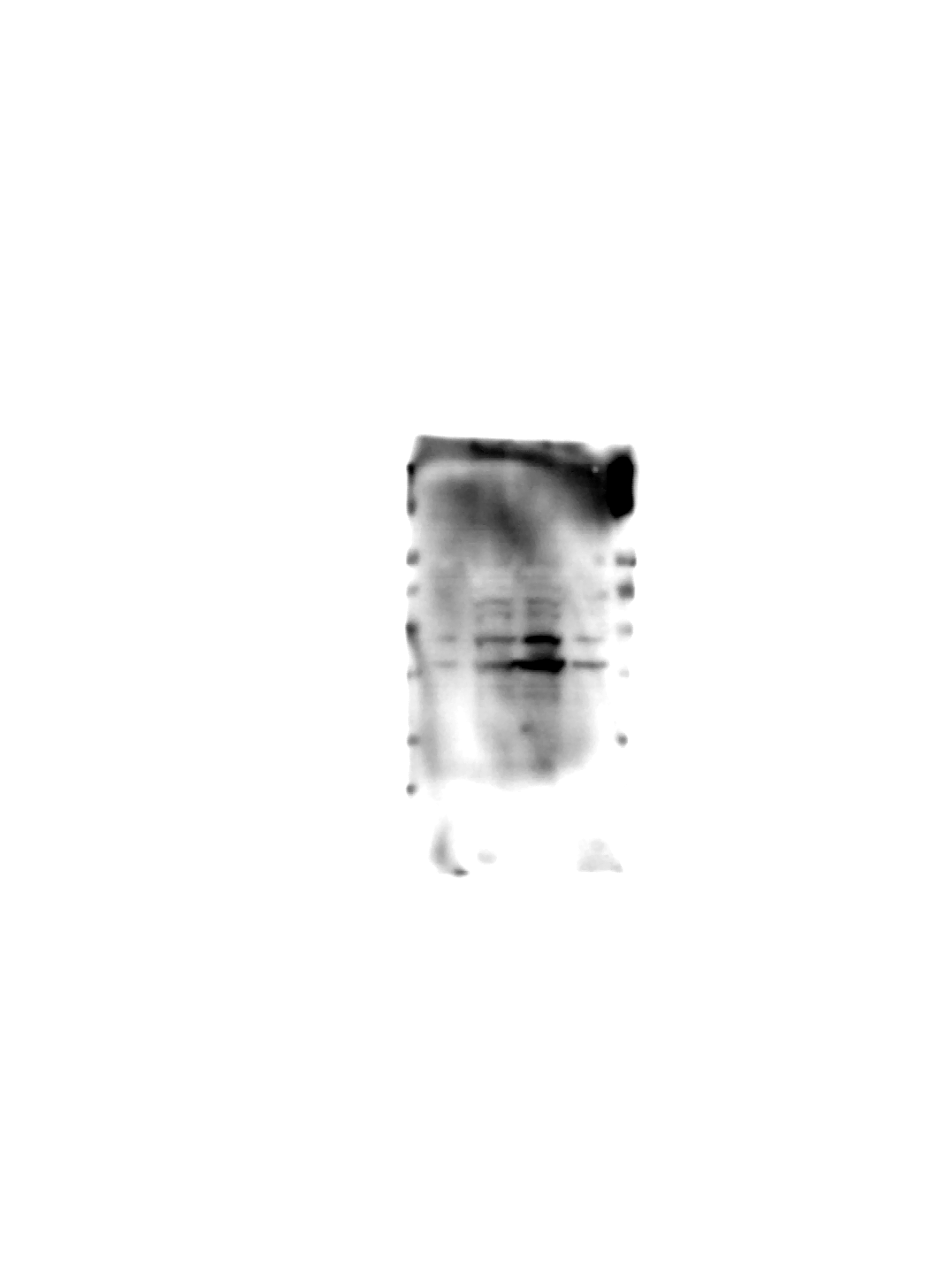

Supplement: Supplementary file 1 [file DataSheet3.zip › Figure4 wb/GSDMD XH 3-sample.tif]

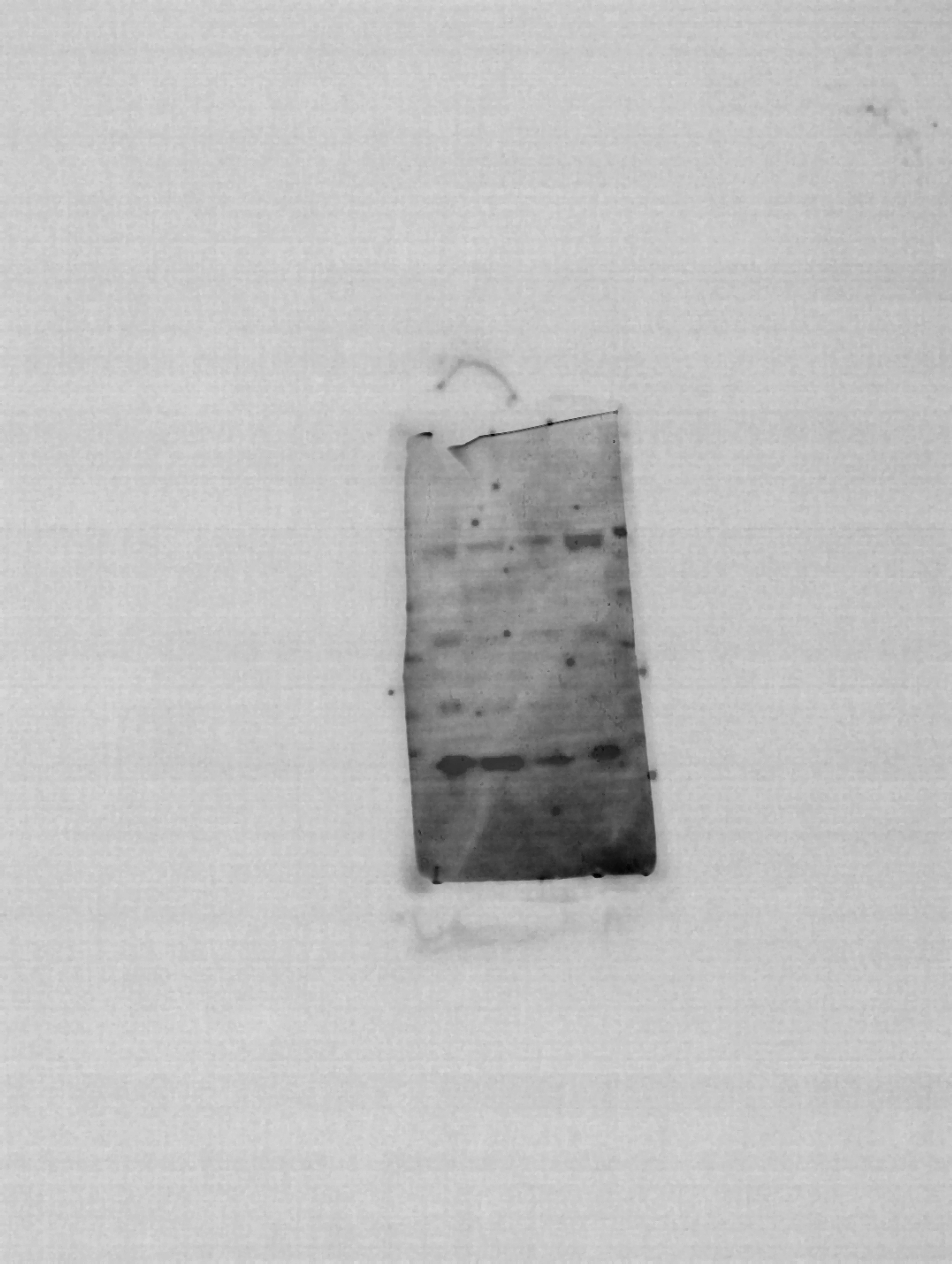

Supplement: Supplementary file 1 [file DataSheet3.zip › Figure4 wb/figure 4 A IKBa 3.tif]

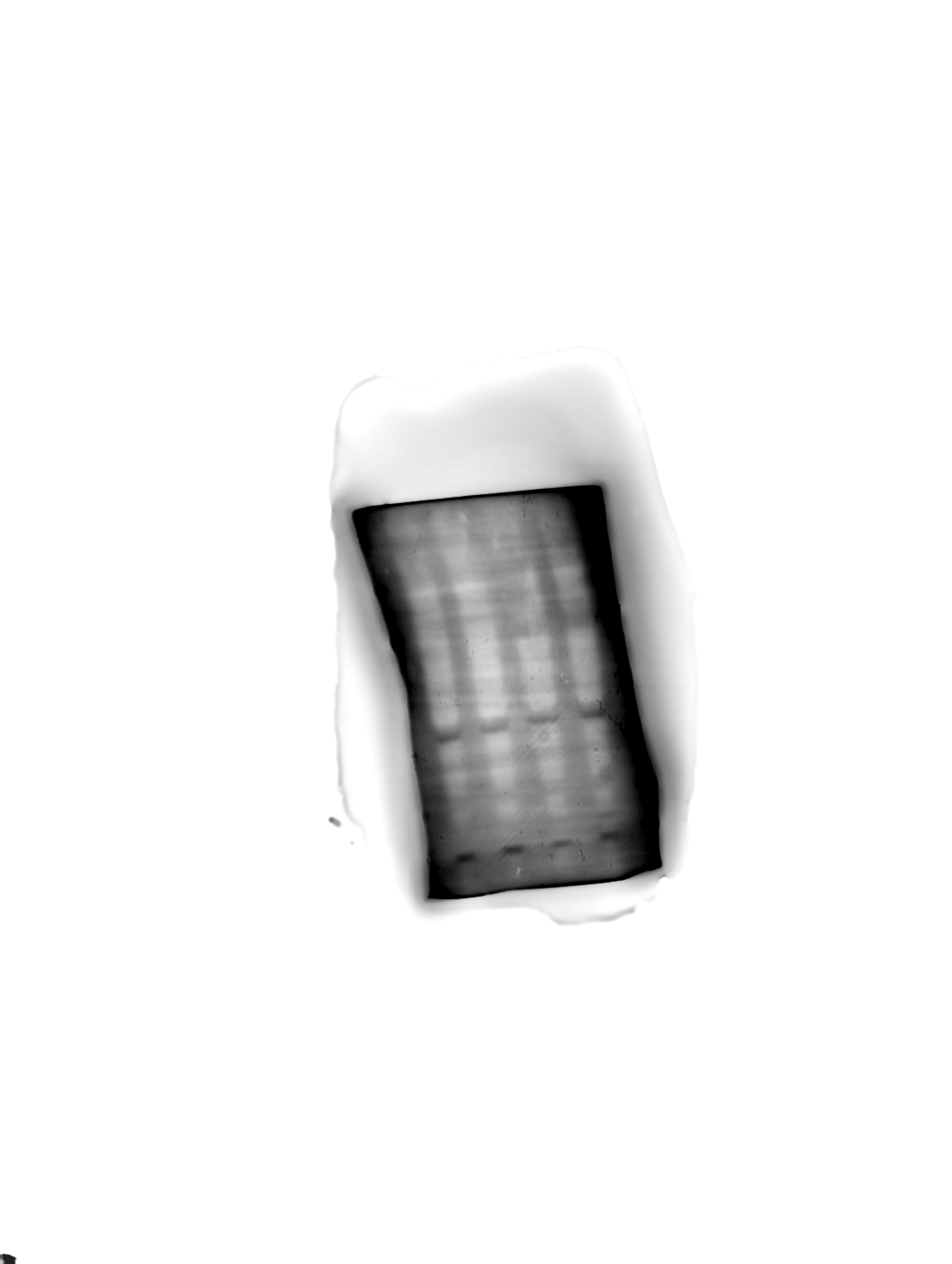

Supplement: Supplementary file 1 [file DataSheet3.zip › Figure4 wb/figure 4 A gapdh 2.tif]

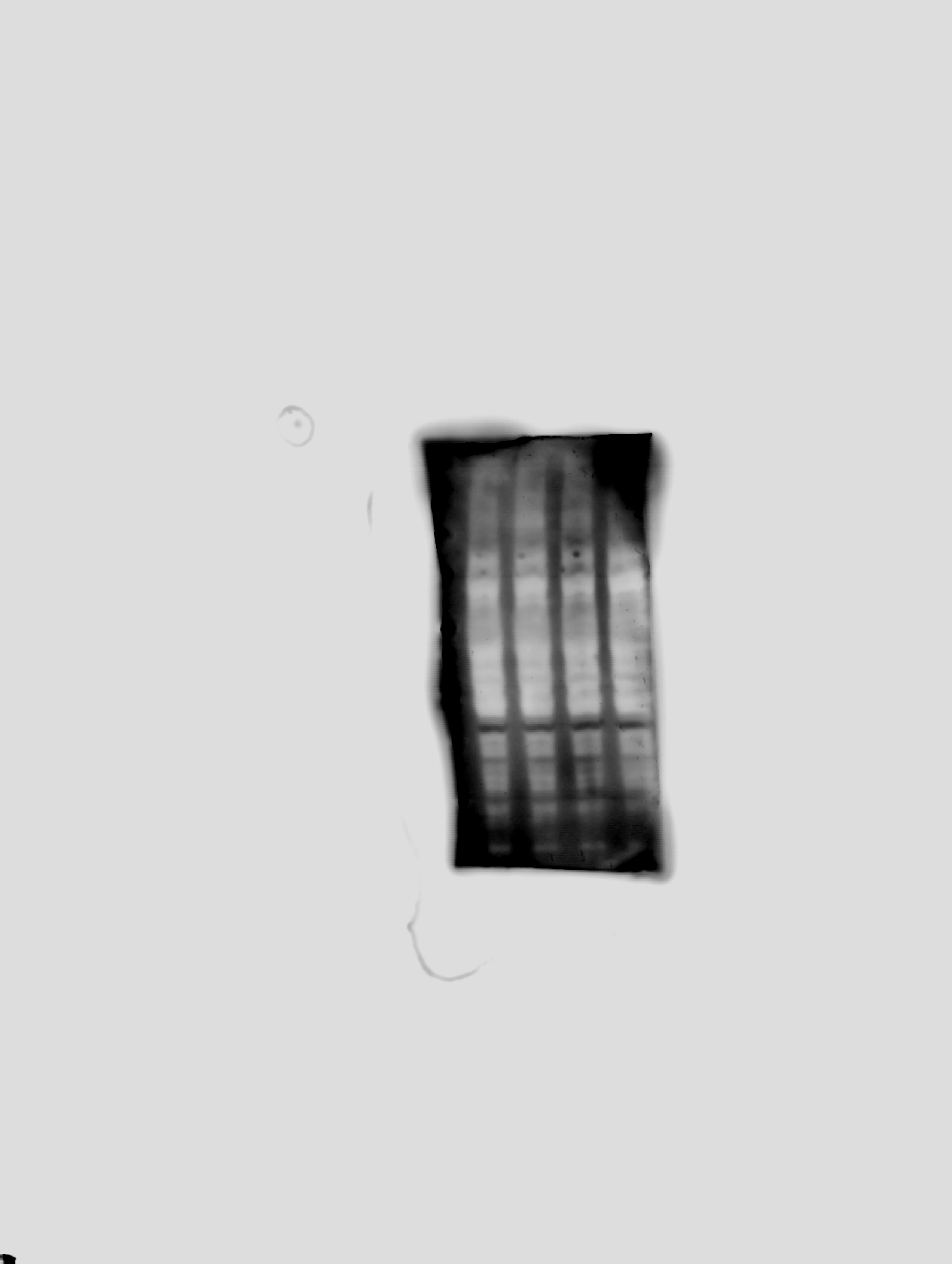

Supplement: Supplementary file 1 [file DataSheet3.zip › Figure4 wb/figure 4 A gapdh 3.tif]

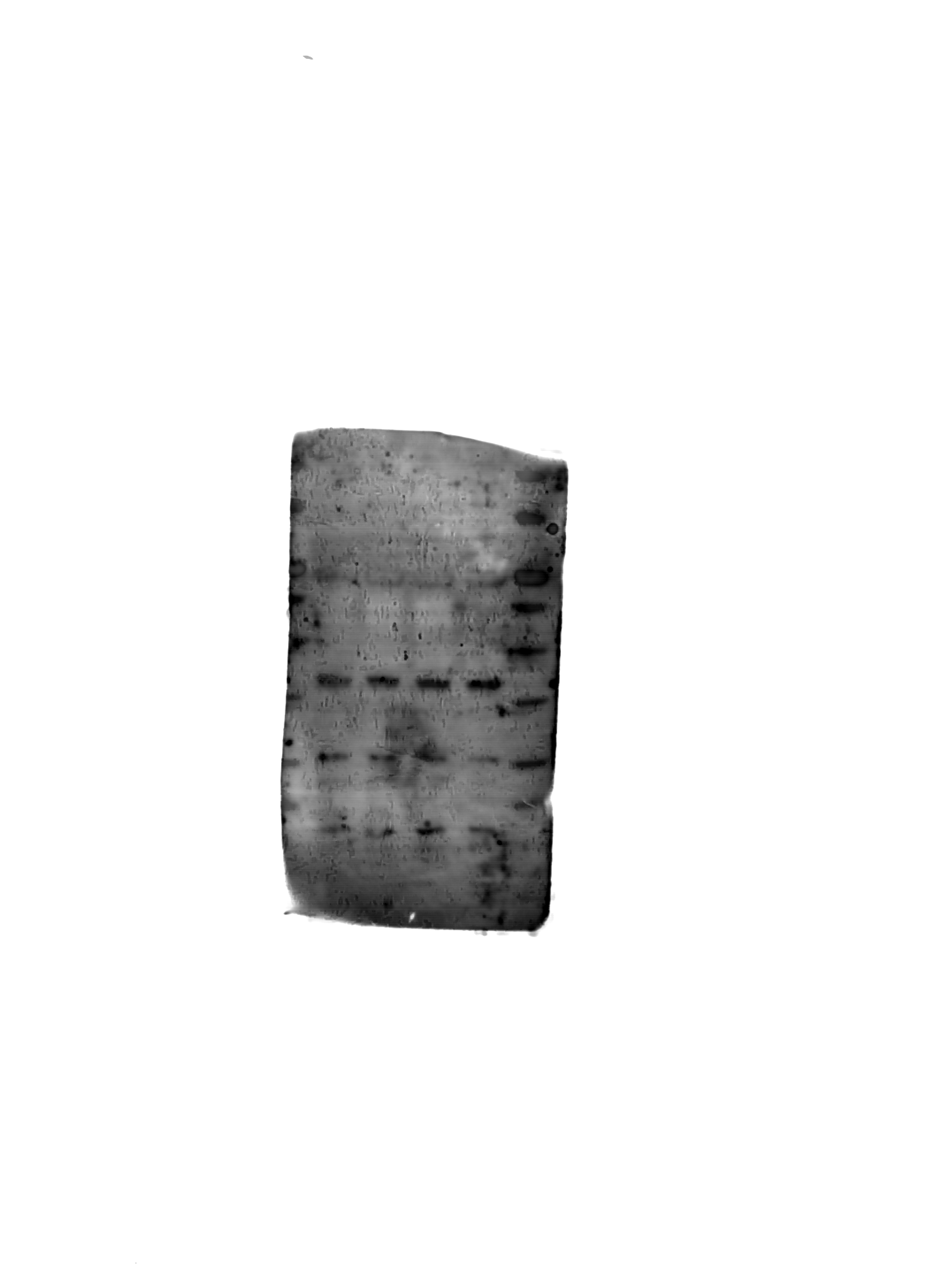

Supplement: Supplementary file 1 [file DataSheet3.zip › Figure4 wb/figure 4 A gapdh.tif]

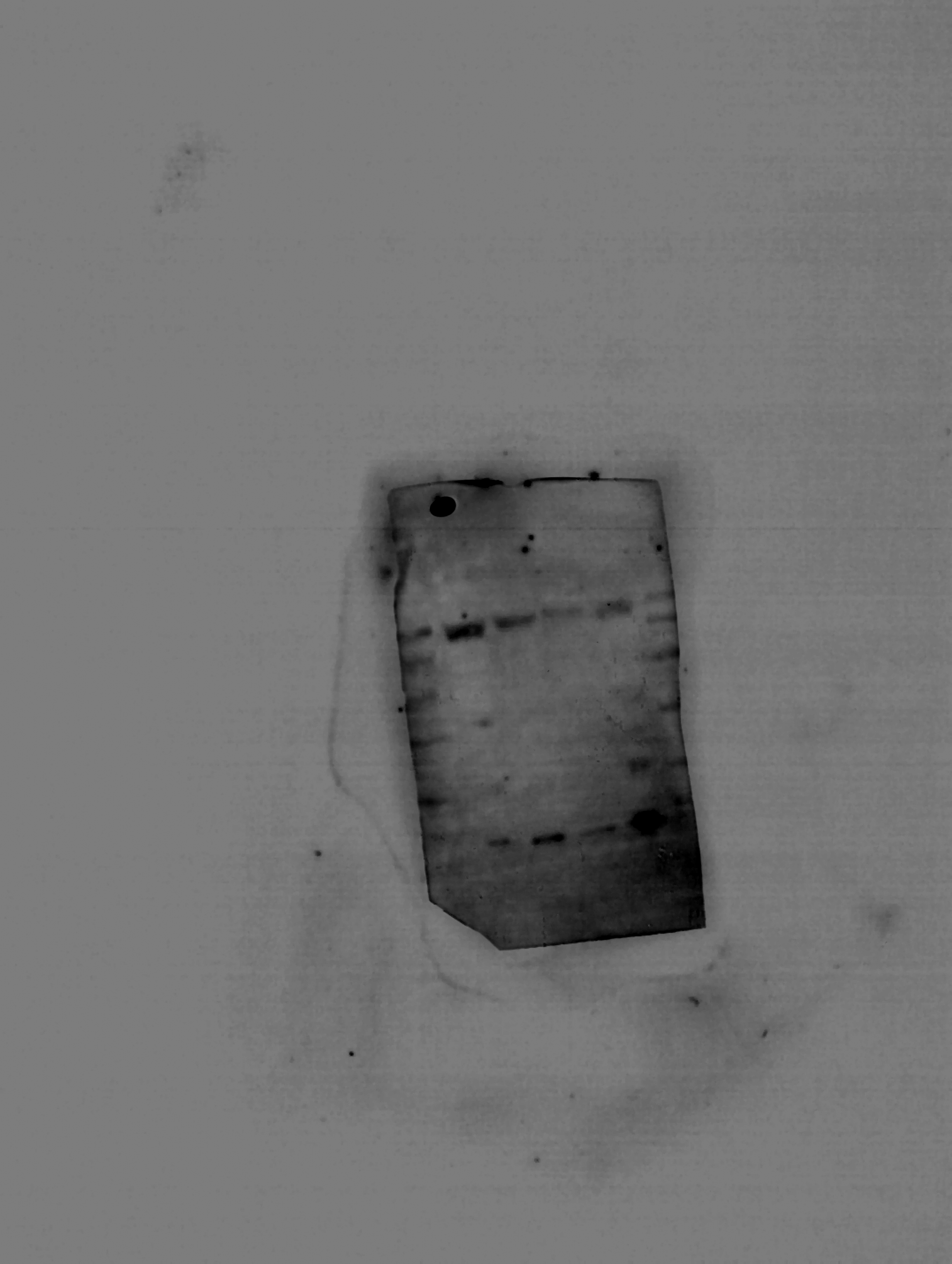

Supplement: Supplementary file 1 [file DataSheet3.zip › Figure4 wb/figure 4 A ikba 2.tif]

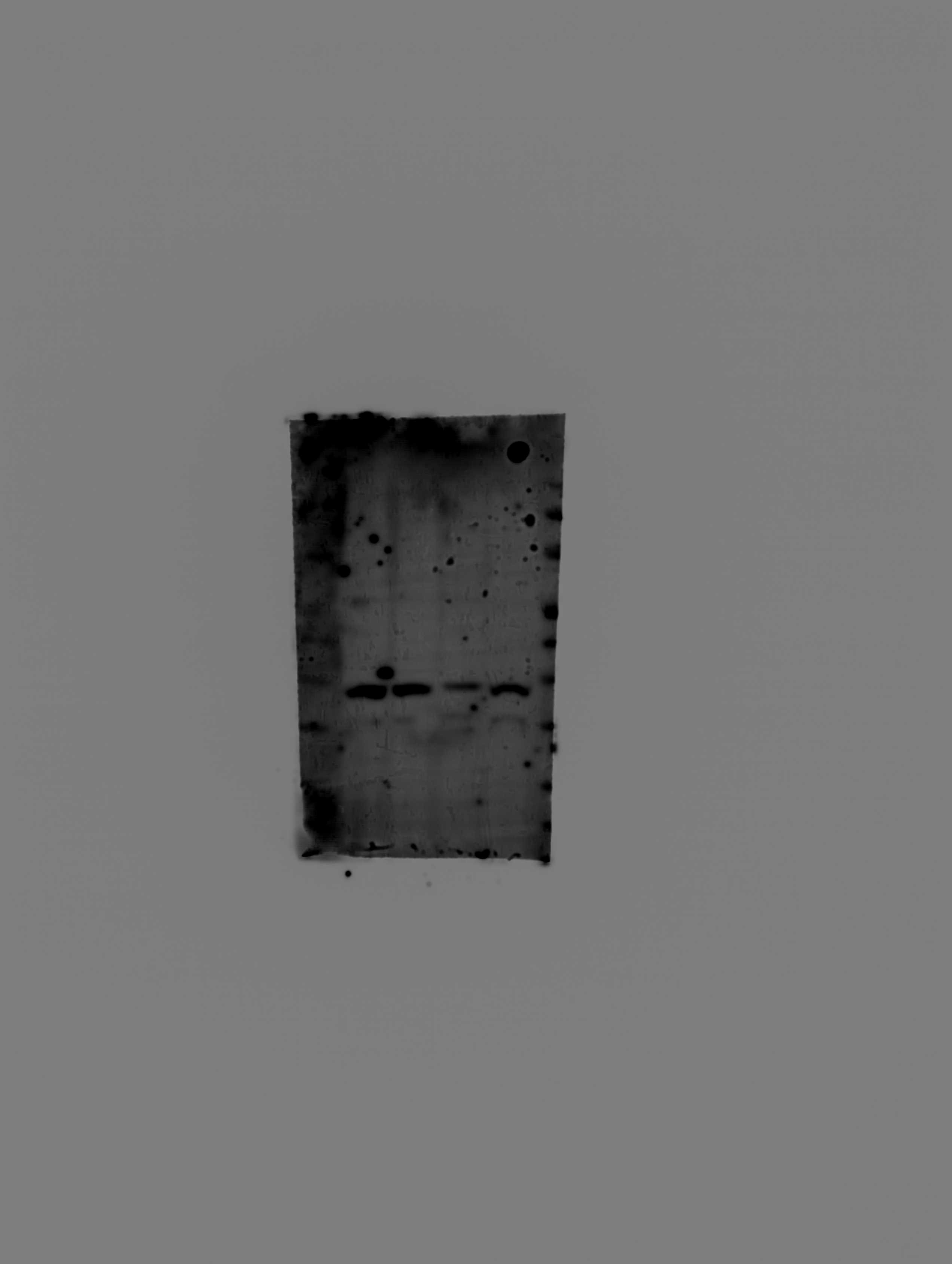

Supplement: Supplementary file 1 [file DataSheet3.zip › Figure4 wb/figure 4 A ikba.tif]

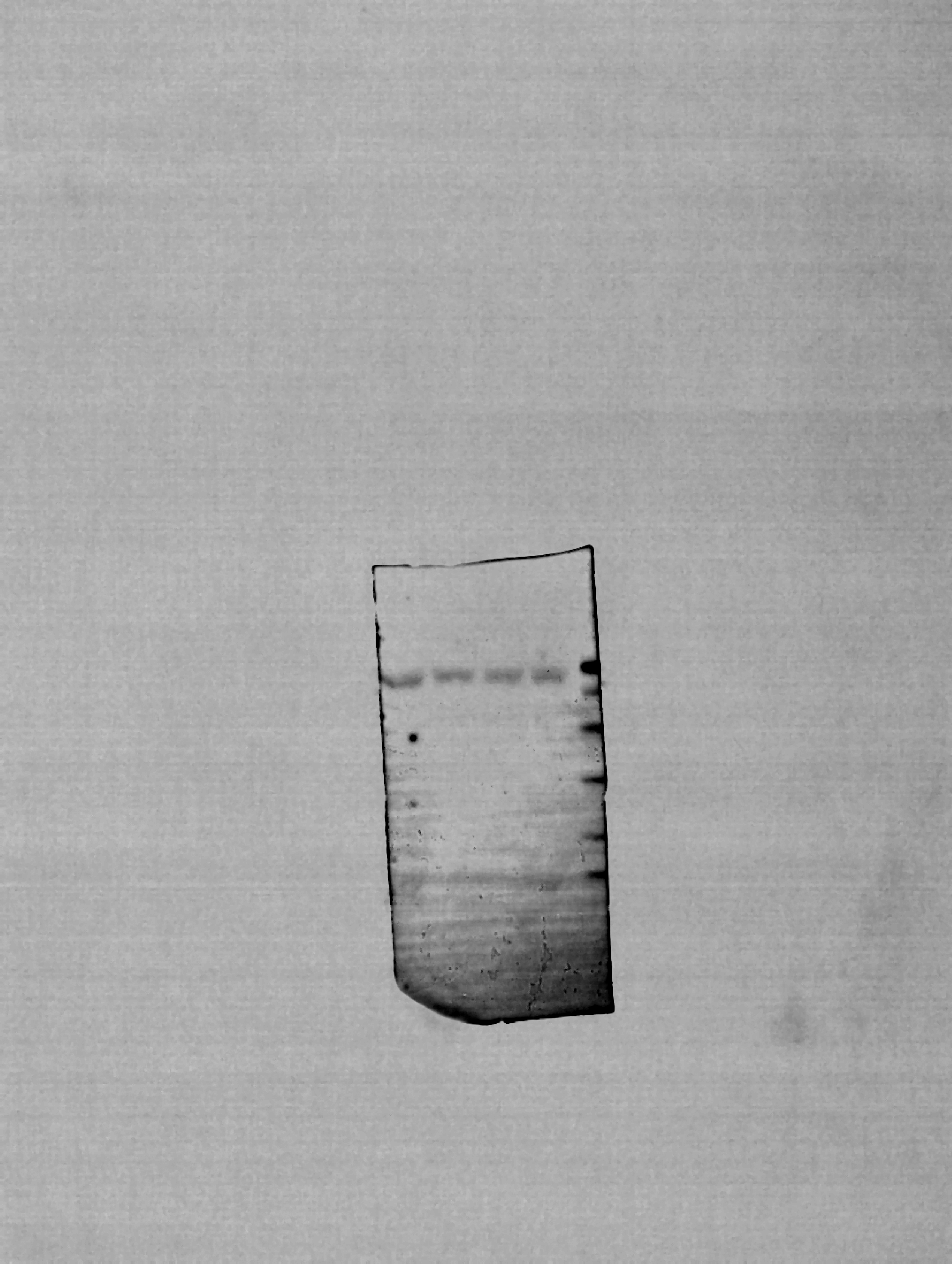

Supplement: Supplementary file 1 [file DataSheet3.zip › Figure4 wb/figure 4 A p65 2.tif]

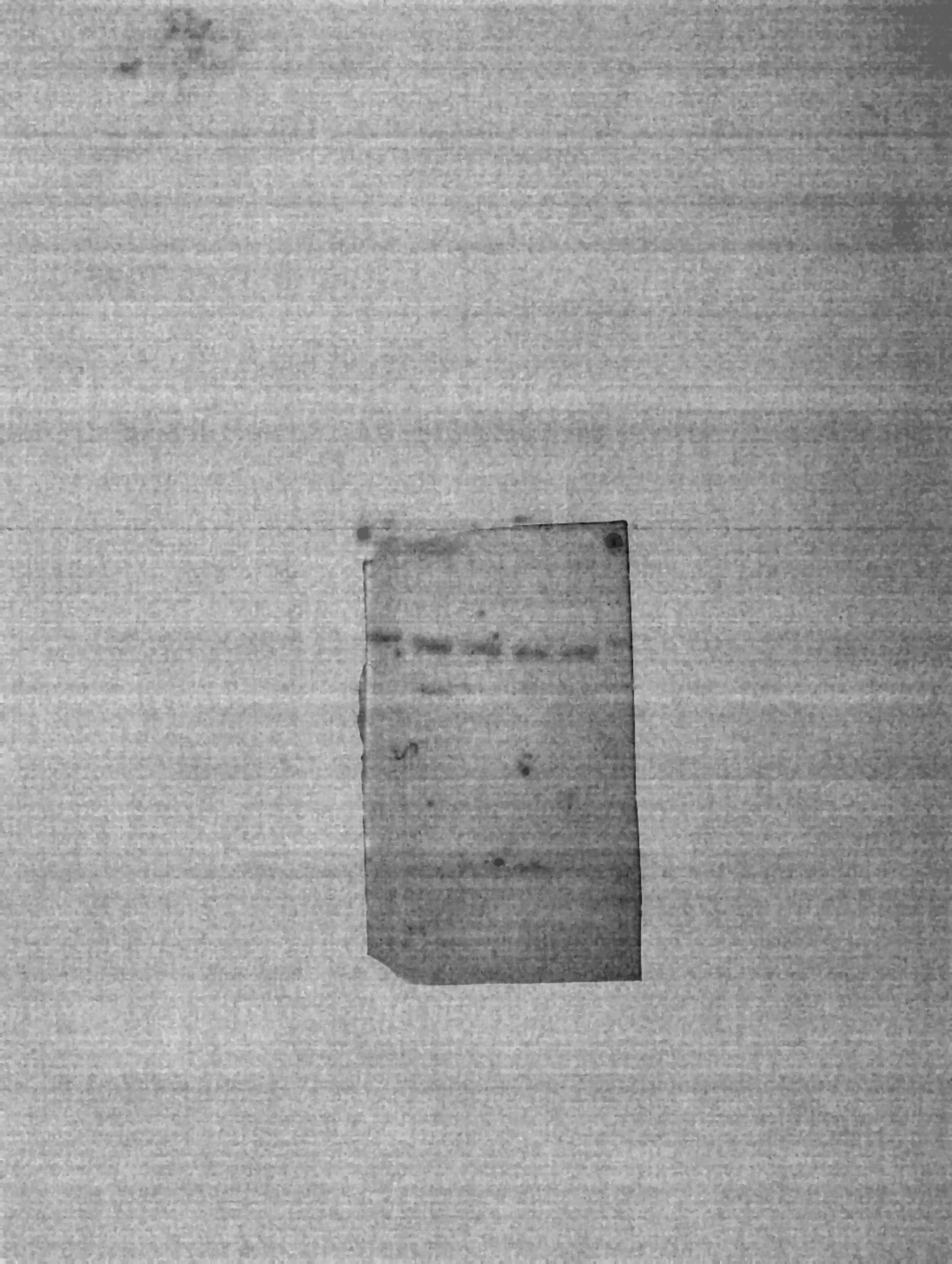

Supplement: Supplementary file 1 [file DataSheet3.zip › Figure4 wb/figure 4 A p65 3.tif]

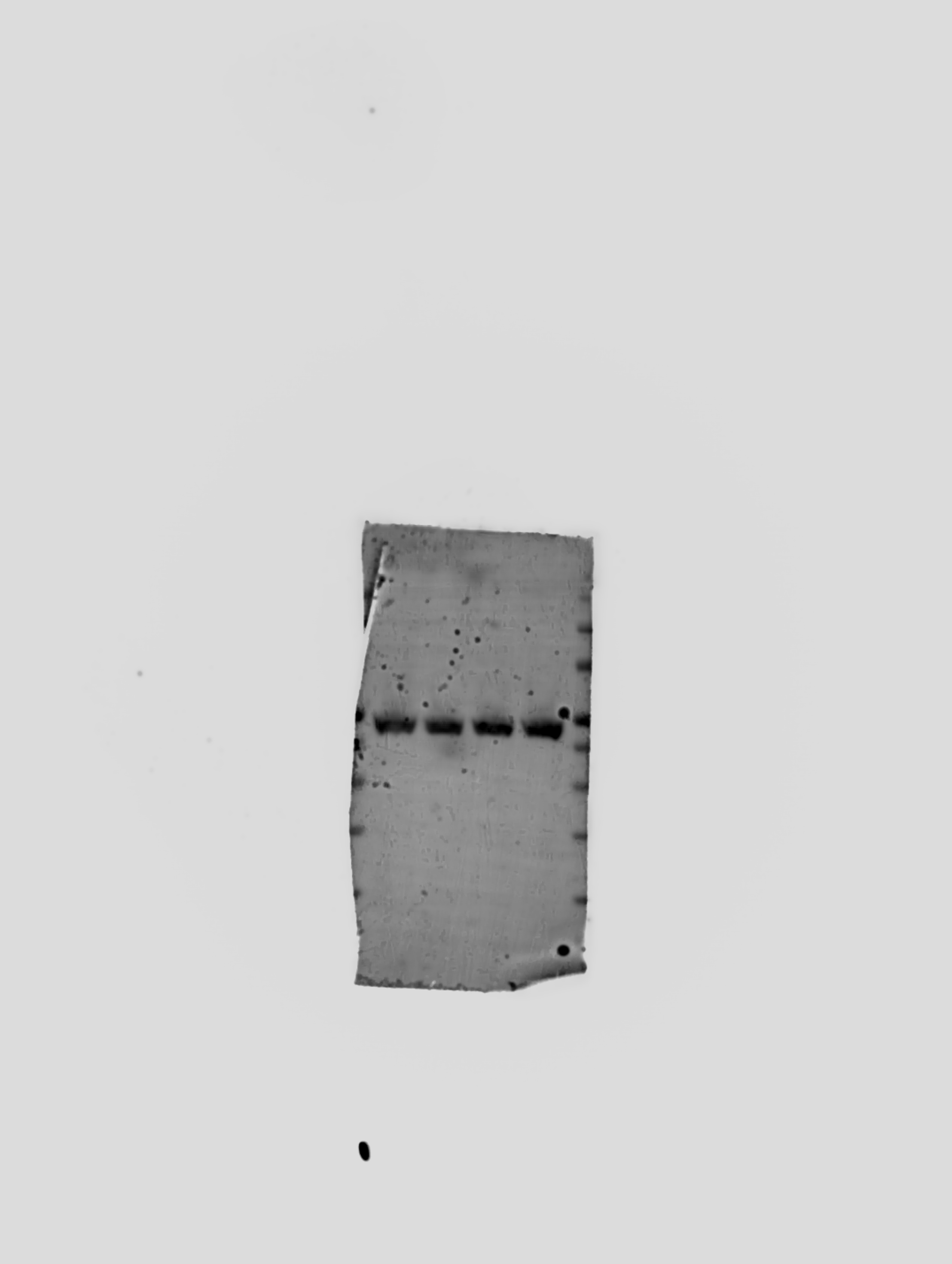

Supplement: Supplementary file 1 [file DataSheet3.zip › Figure4 wb/figure 4 A p65.tif]

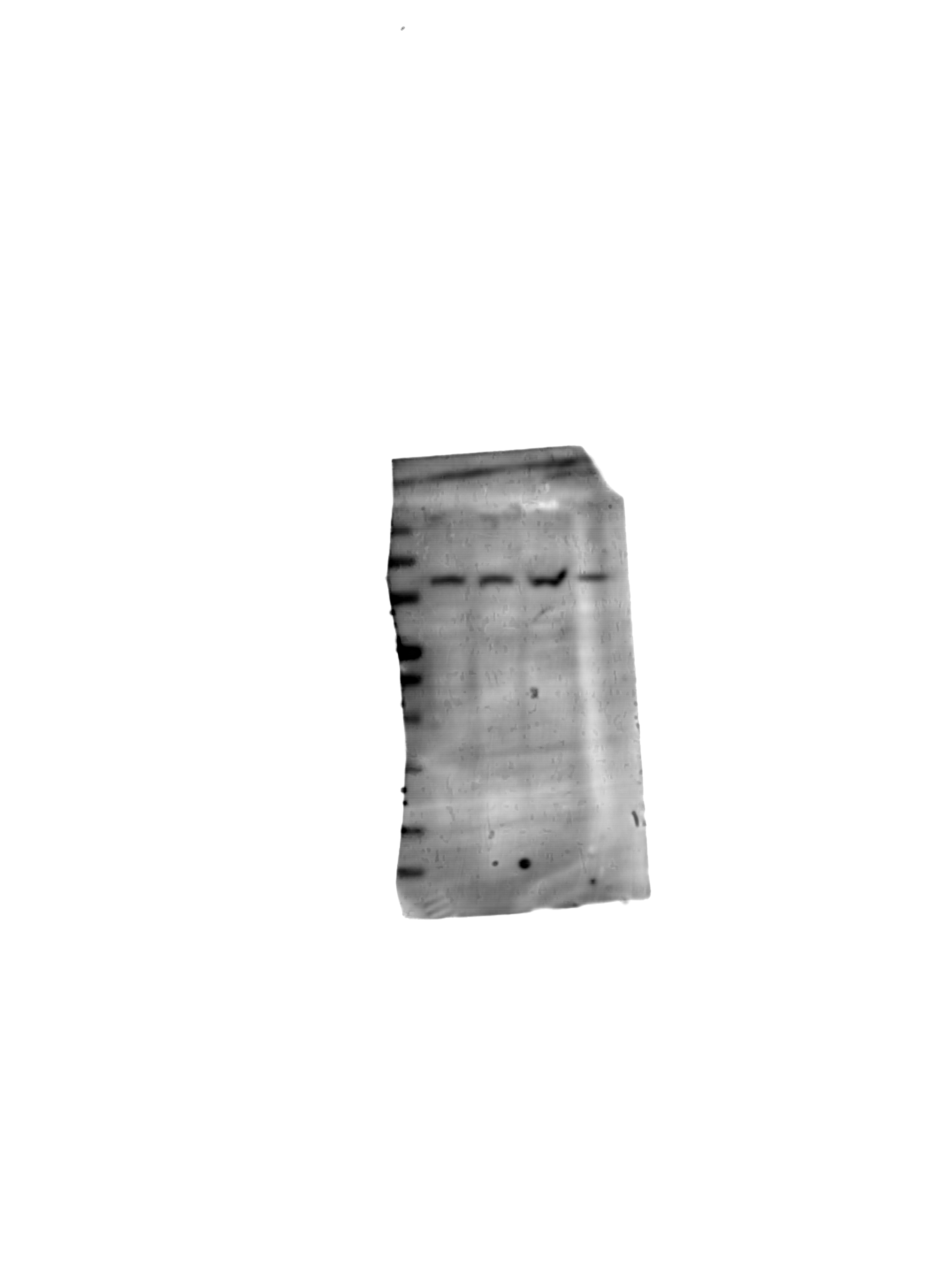

Supplement: Supplementary file 1 [file DataSheet3.zip › Figure4 wb/figure 4 A pp65.tif]

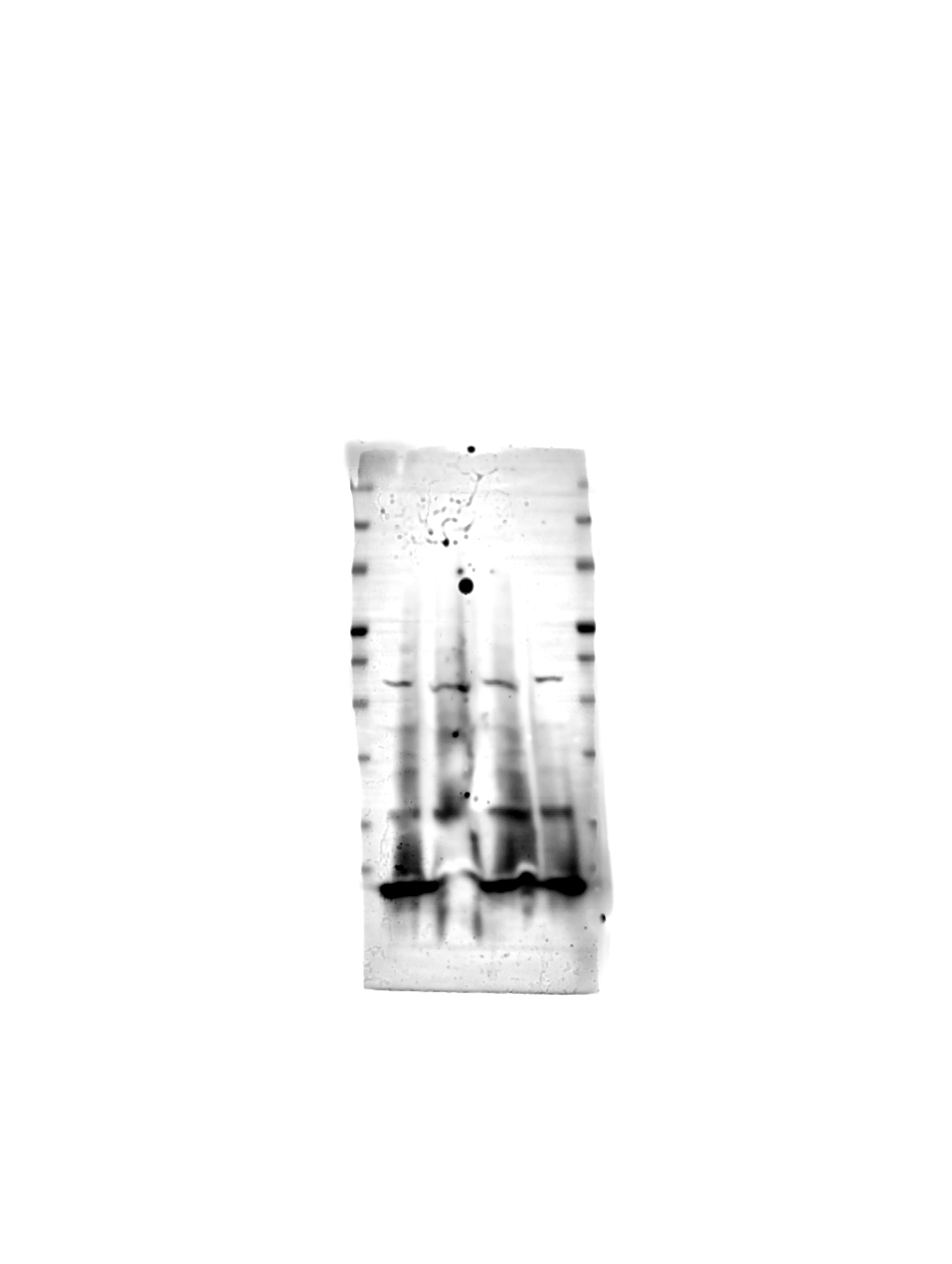

Supplement: Supplementary file 1 [file DataSheet3.zip › Figure4 wb/figure 4 B caspase1.tif]

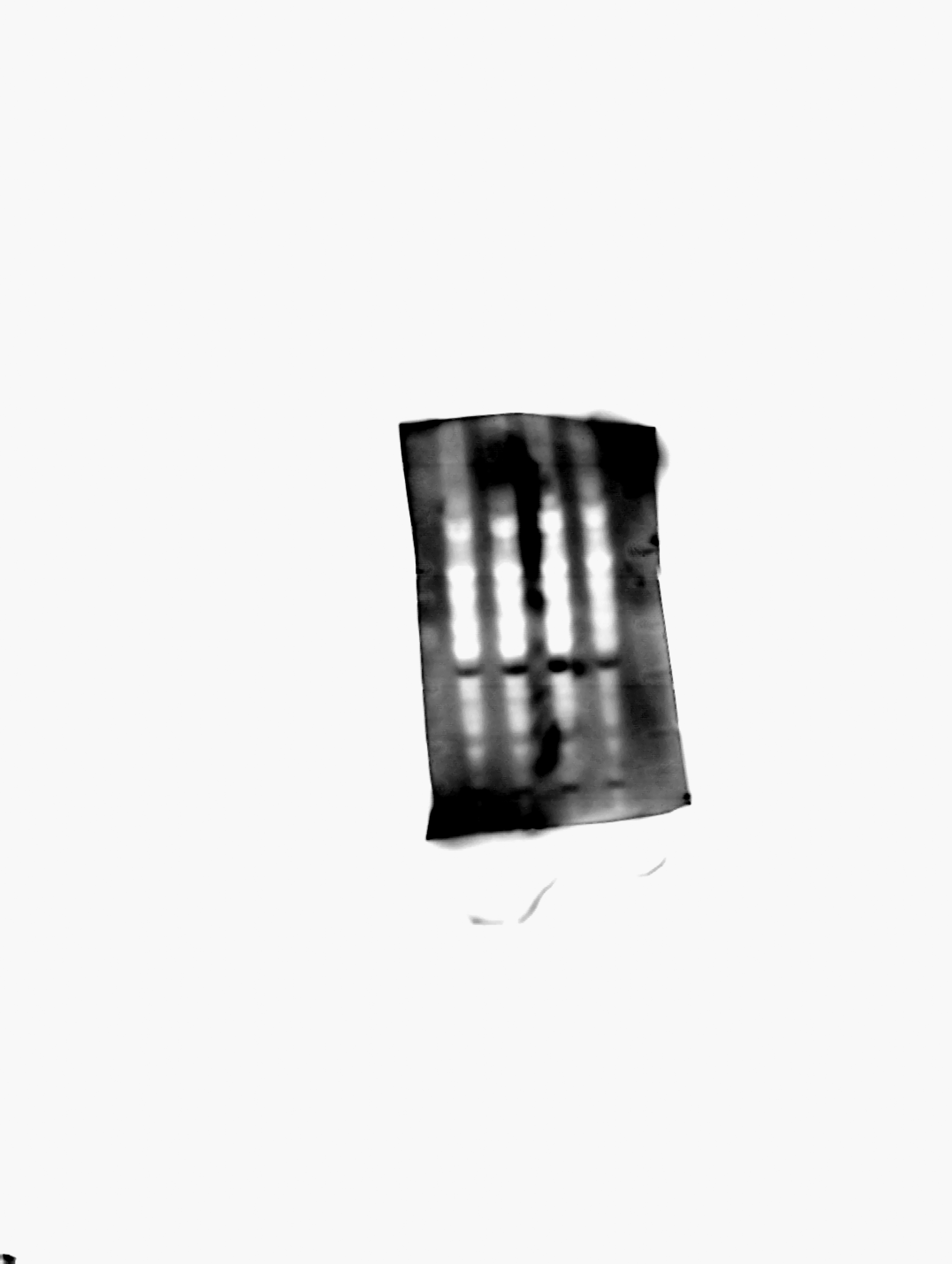

Supplement: Supplementary file 1 [file DataSheet3.zip › Figure4 wb/figure 4 B gapdh 2.tif]

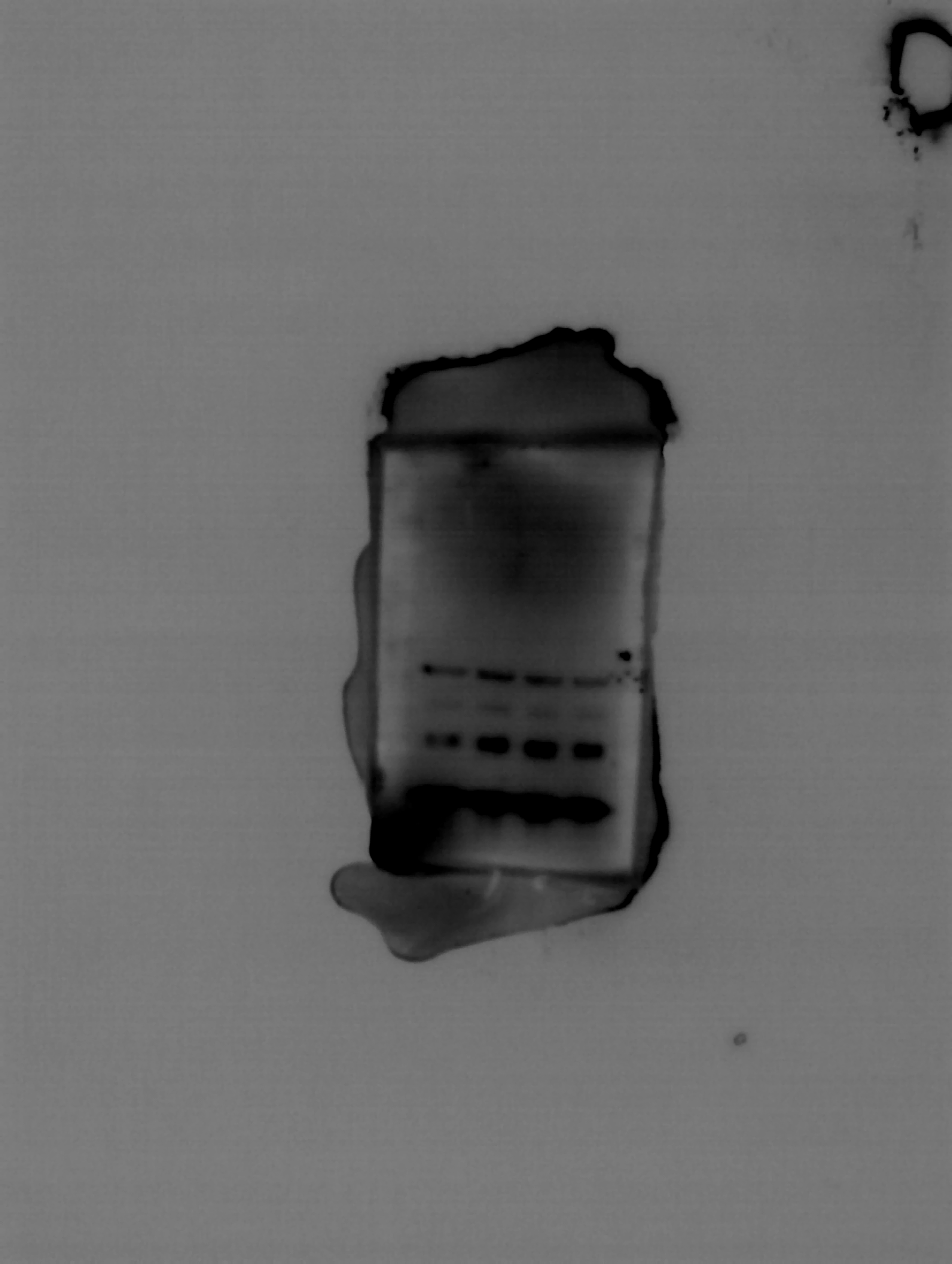

Supplement: Supplementary file 1 [file DataSheet3.zip › Figure4 wb/figure 4 B gapdh 3.tif]

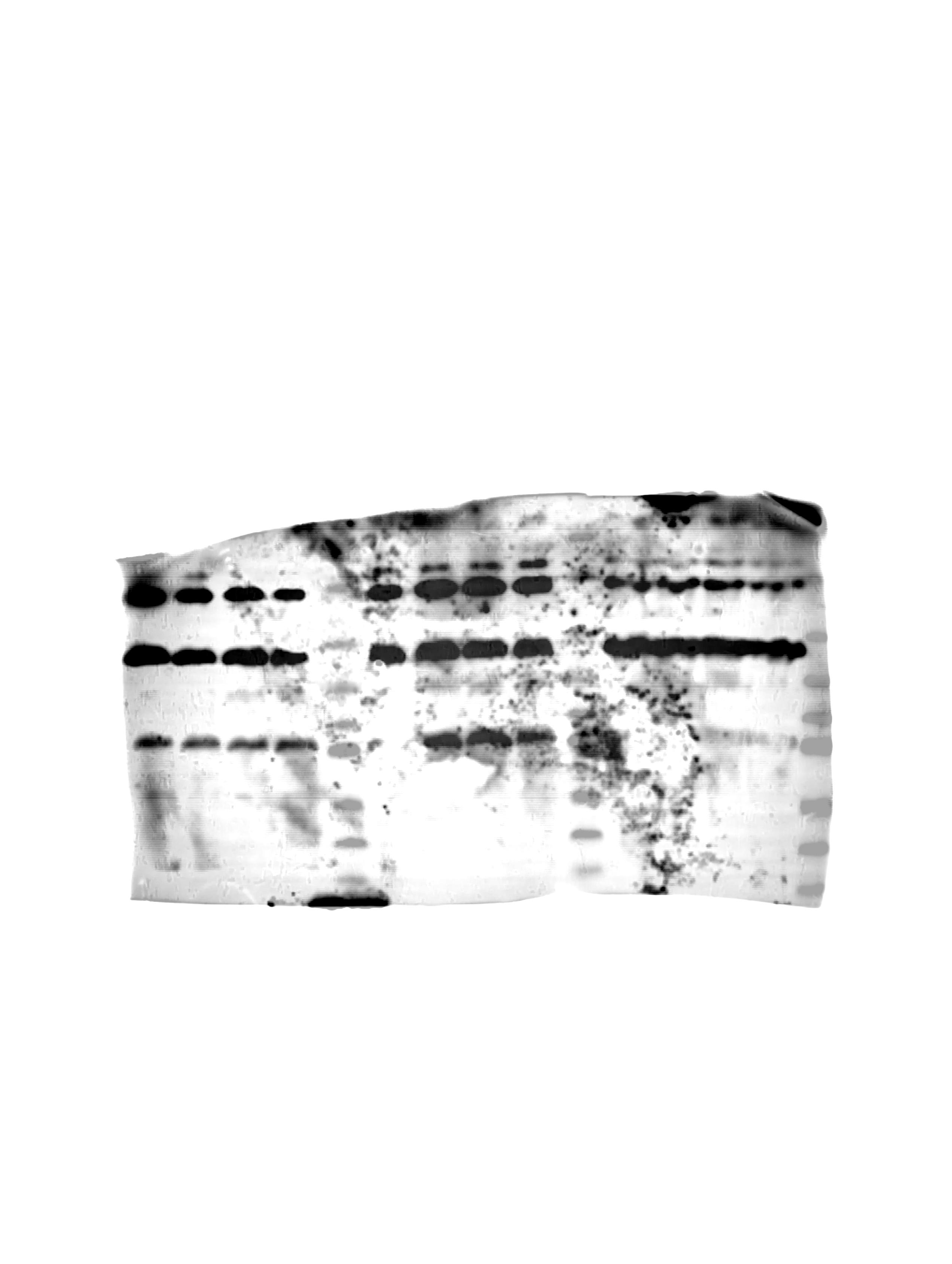

Supplement: Supplementary file 1 [file DataSheet3.zip › Figure4 wb/figure 4 B gapdh and figure5 B gapdh.tif]

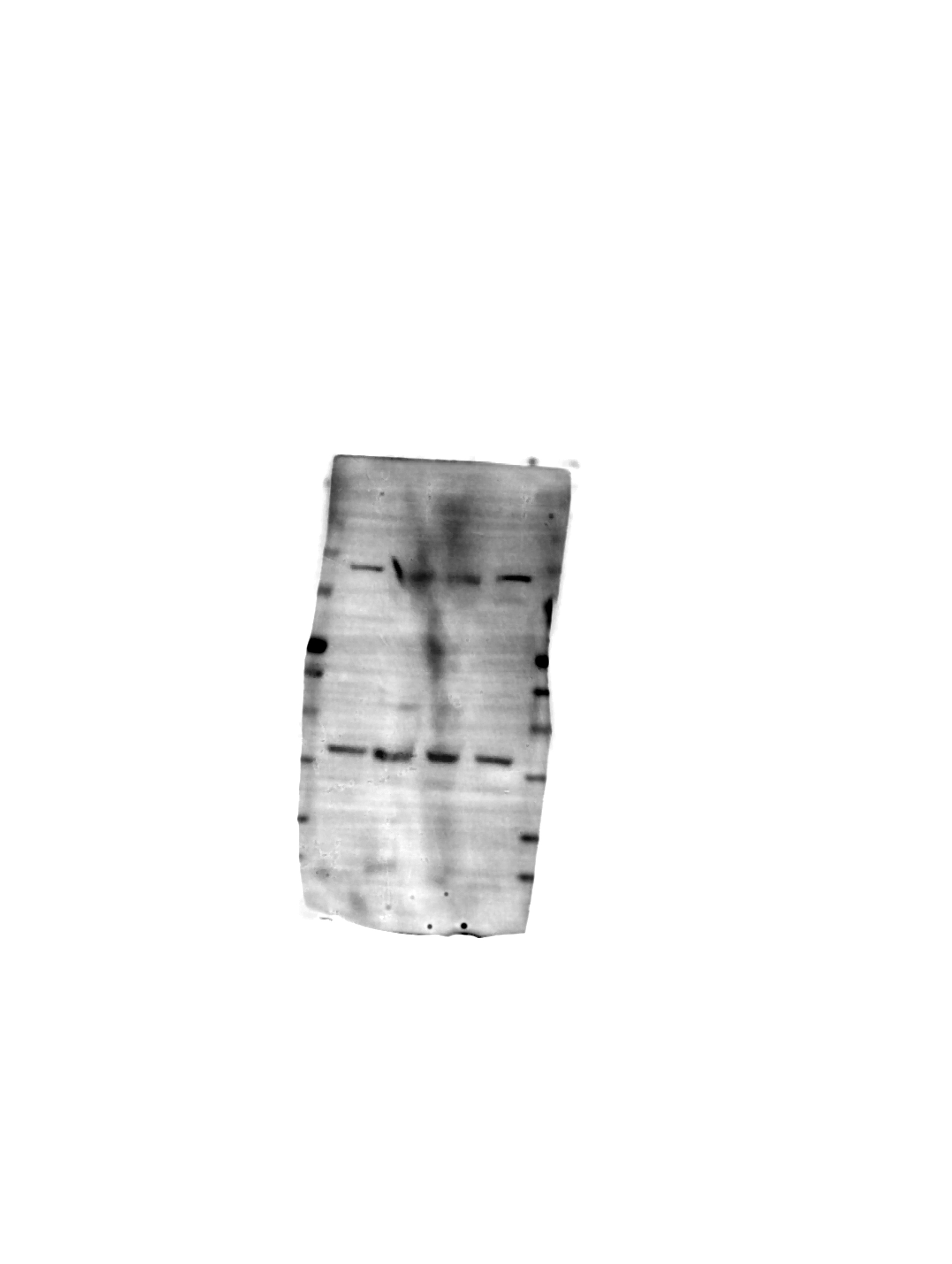

Supplement: Supplementary file 1 [file DataSheet3.zip › Figure4 wb/figure 4 B gsdmd 2.tif]

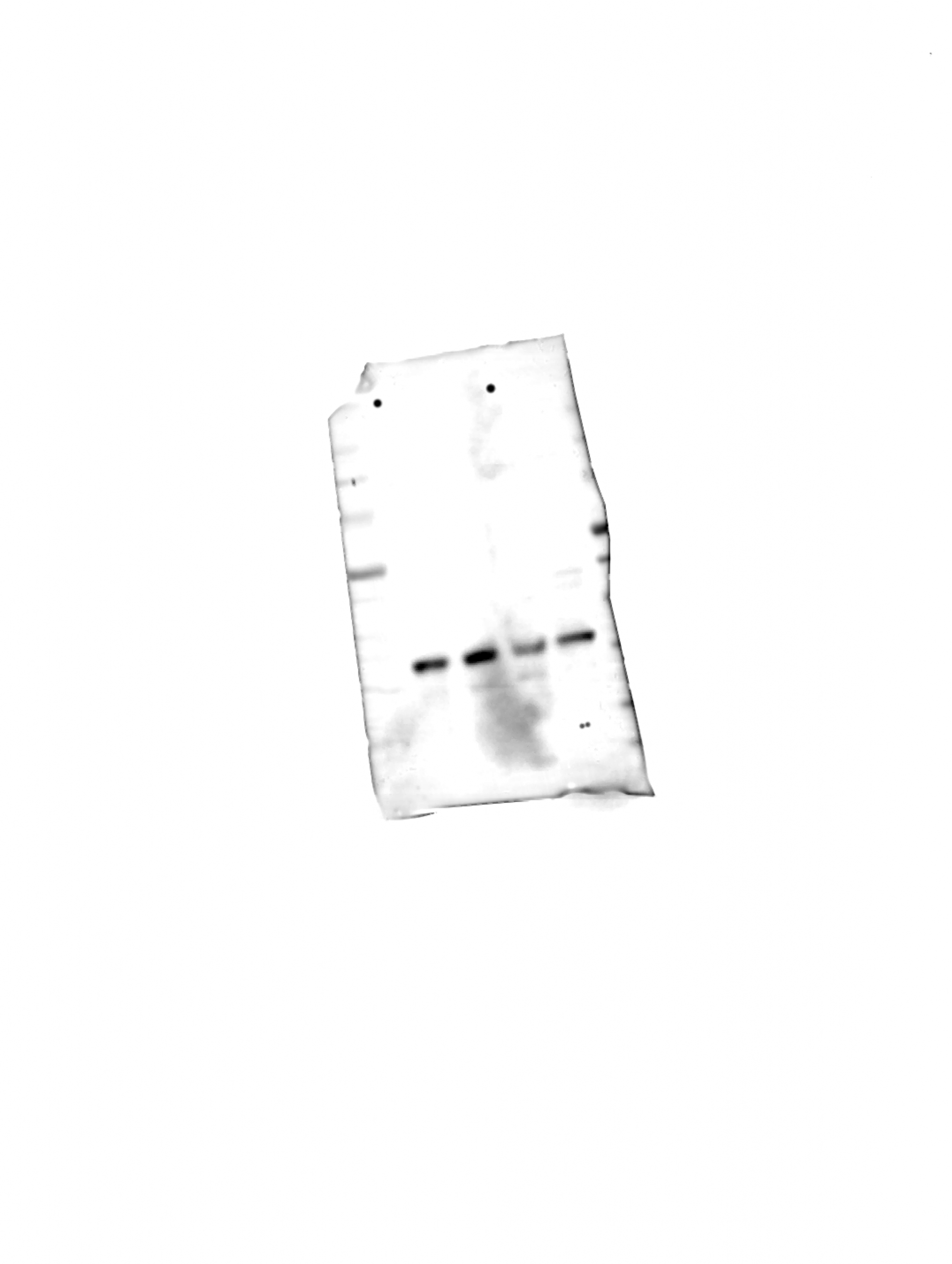

Supplement: Supplementary file 1 [file DataSheet3.zip › Figure4 wb/figure 4 B gsdmd 3.tif]

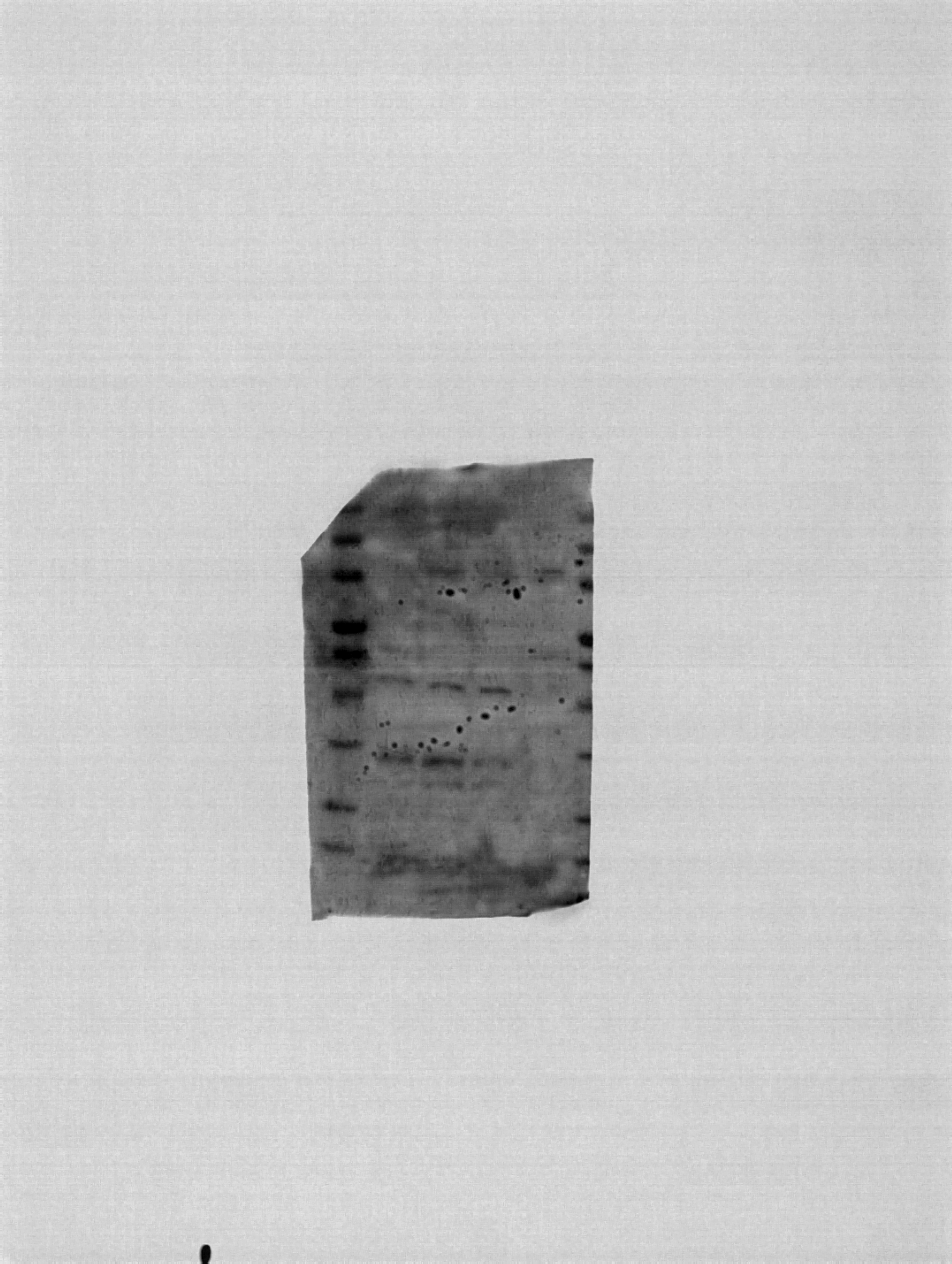

Supplement: Supplementary file 1 [file DataSheet3.zip › Figure4 wb/figure 4 B gsdmd.tif]

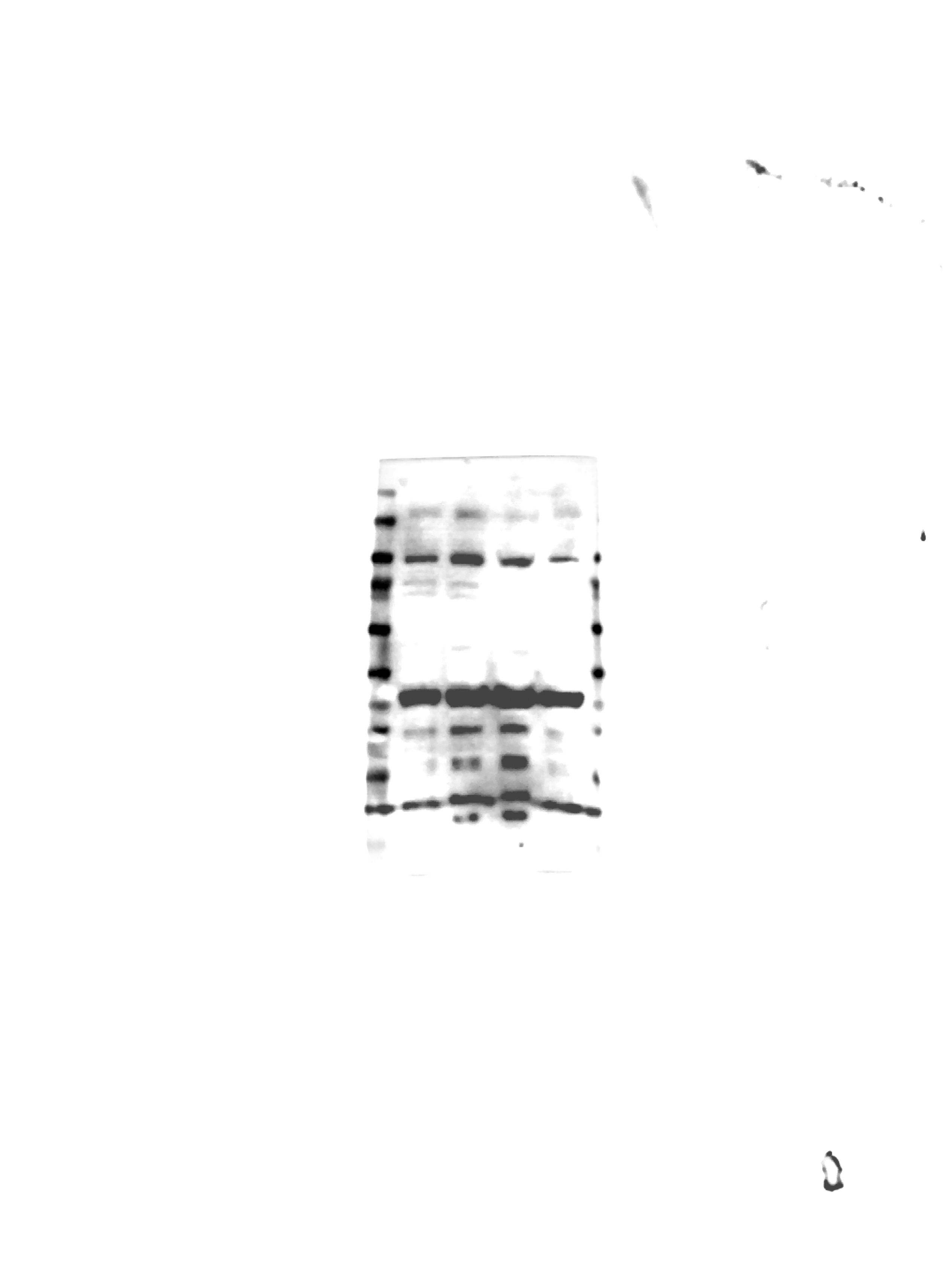

Supplement: Supplementary file 1 [file DataSheet3.zip › Figure4 wb/figure 4 B nlrp3 2.tif]

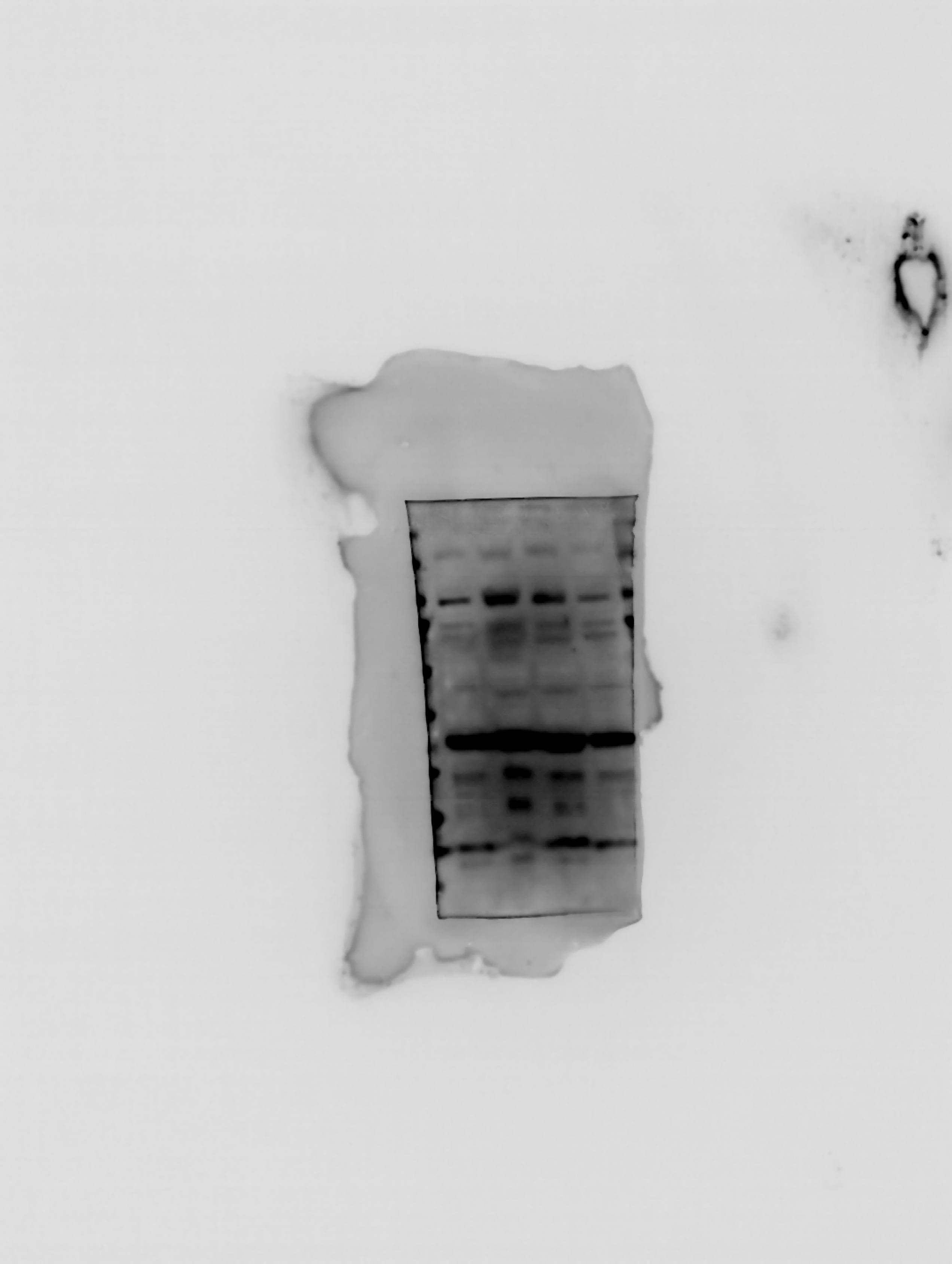

Supplement: Supplementary file 1 [file DataSheet3.zip › Figure4 wb/figure 4 B nlrp3 3.tif]

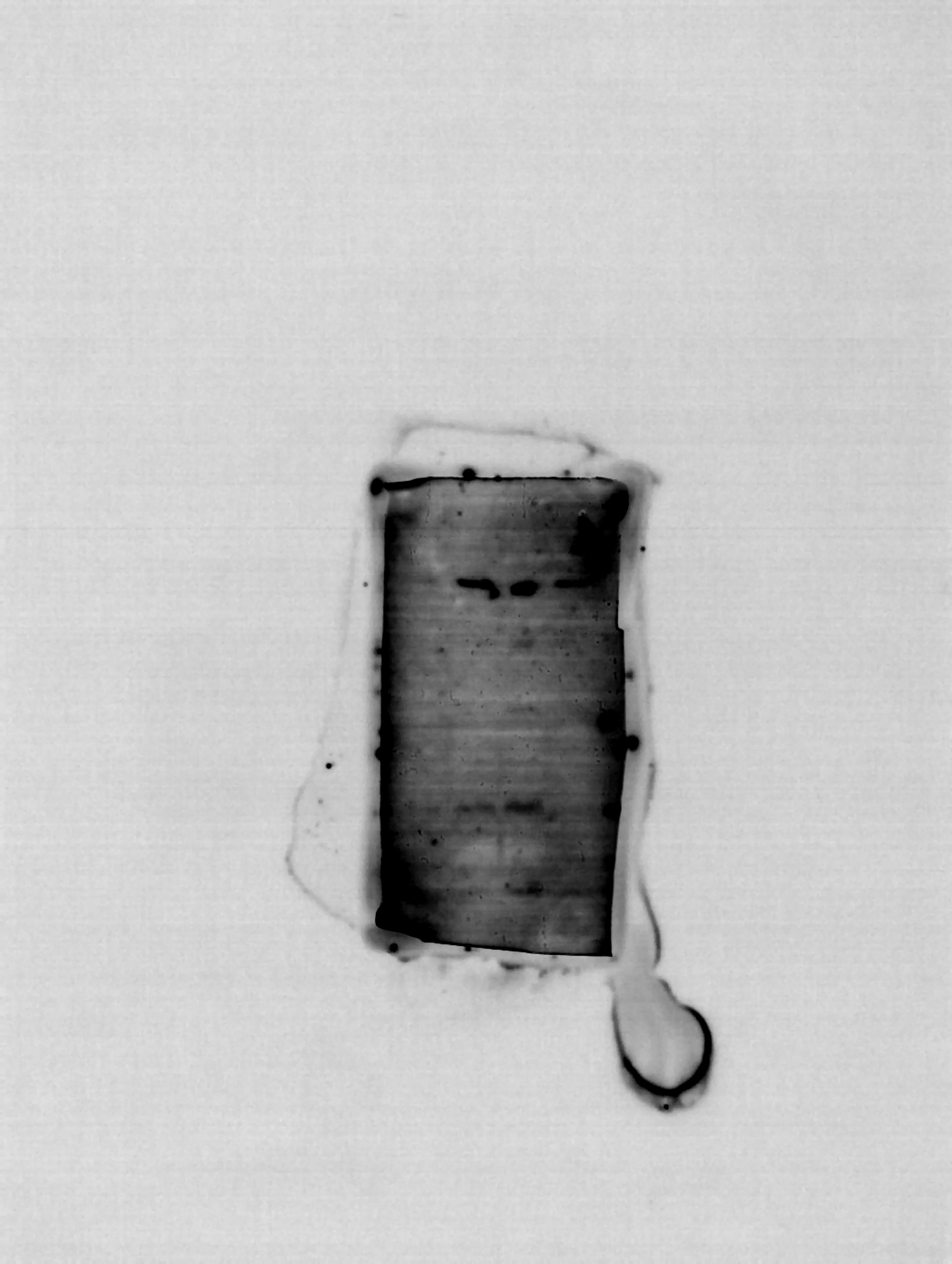

Supplement: Supplementary file 1 [file DataSheet3.zip › Figure4 wb/figure4 A pp65 2.tif]

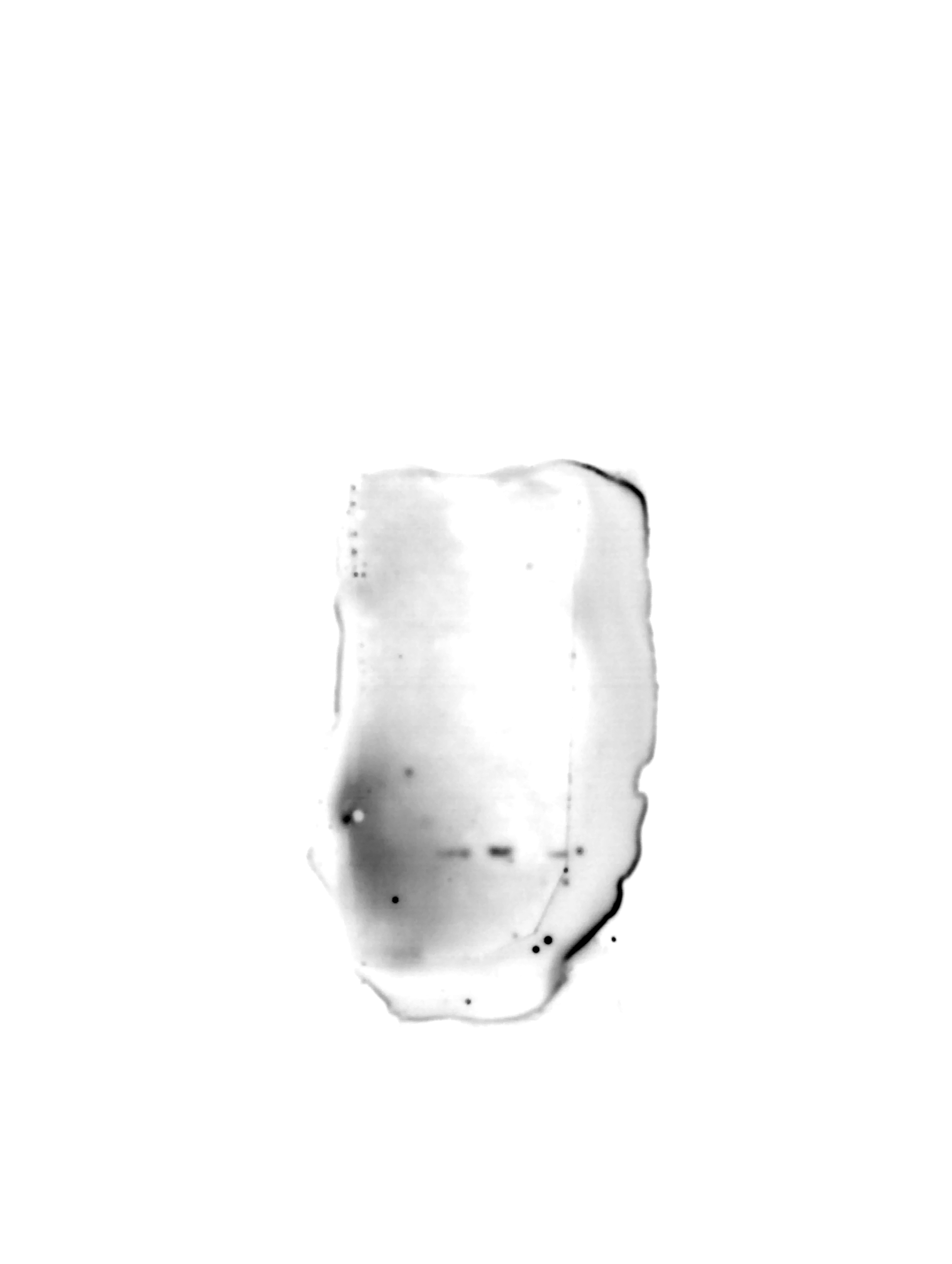

Supplement: Supplementary file 1 [file DataSheet3.zip › Figure4 wb/figure4 A pp65 3.tif]

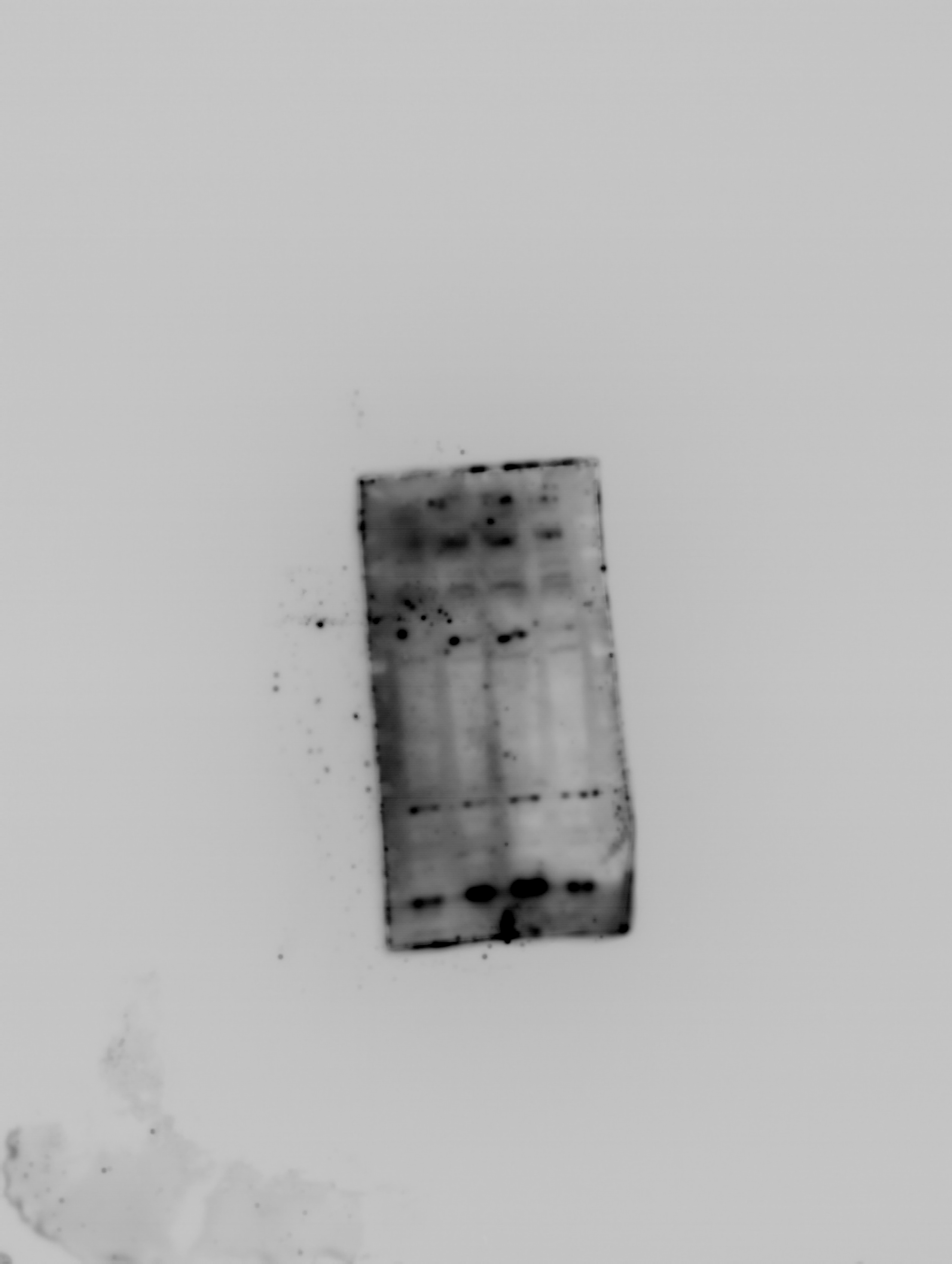

Supplement: Supplementary file 1 [file DataSheet3.zip › Figure4 wb/figure4 B nlrp3.tif]

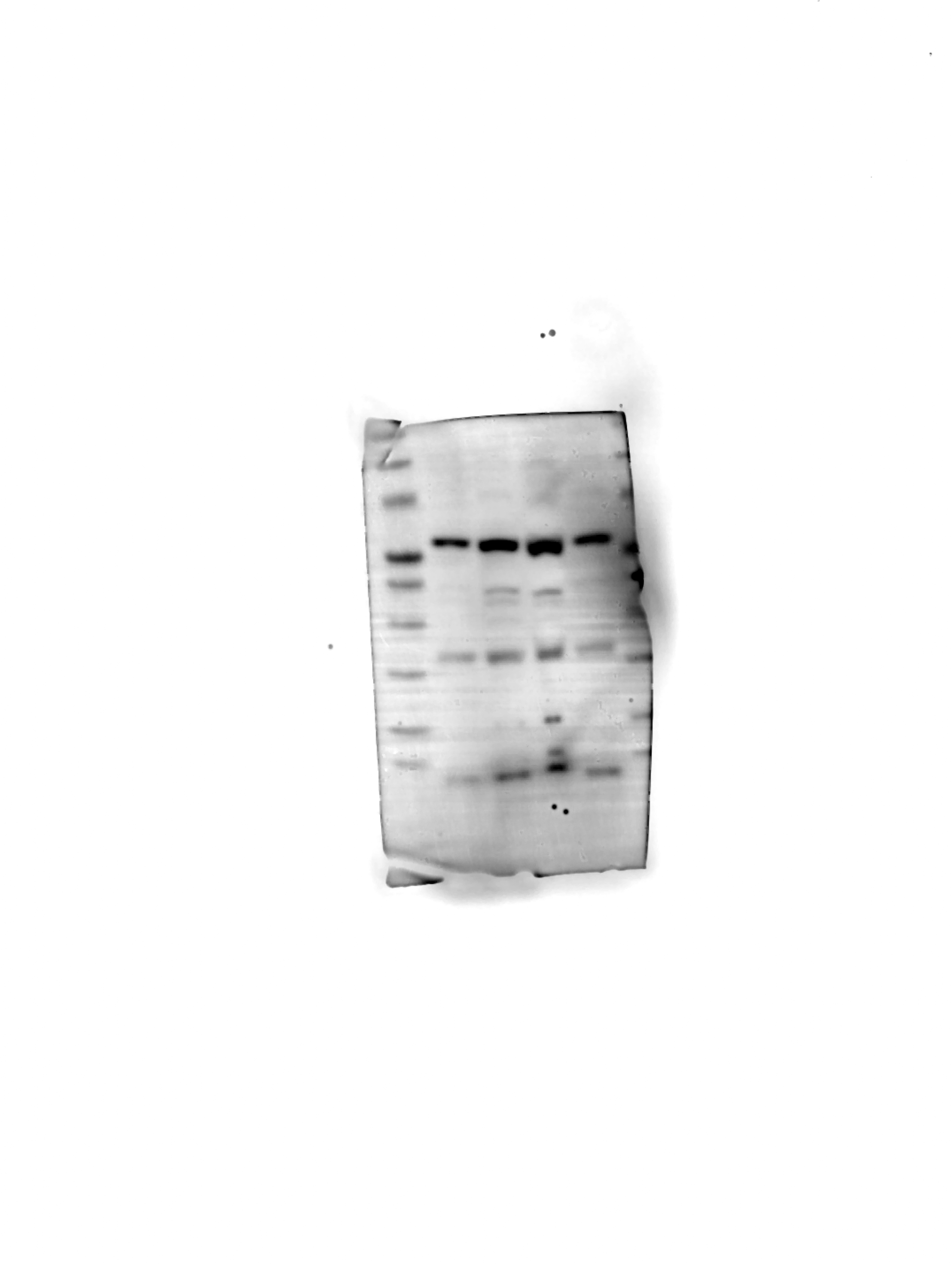

Supplement: Supplementary file 3 [file DataSheet4.zip › Figure5 wb/Figure 5 B adamts 5 1 .tif]

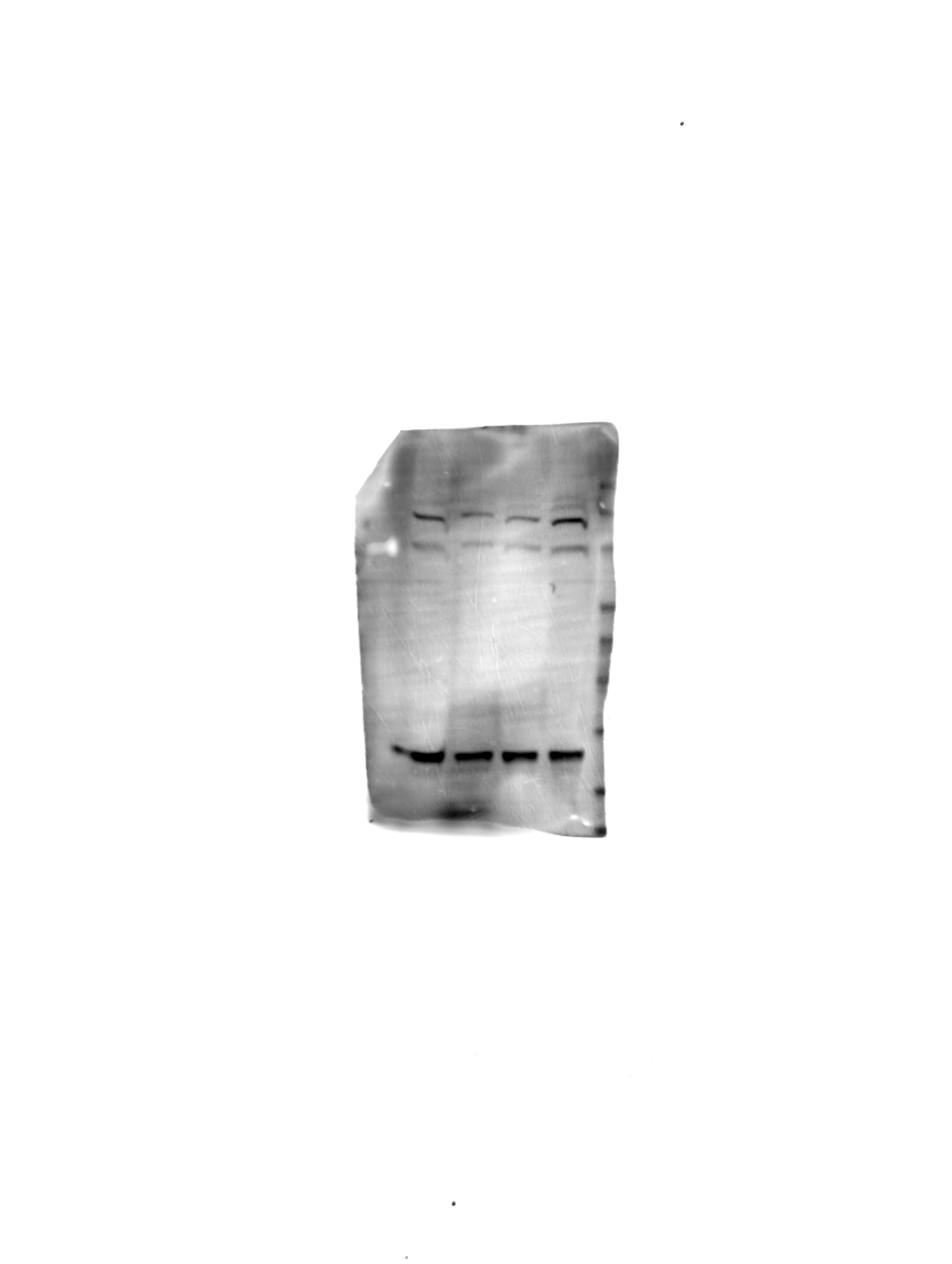

Supplement: Supplementary file 3 [file DataSheet4.zip › Figure5 wb/Figure 5 COL2A1 2 1.tif]

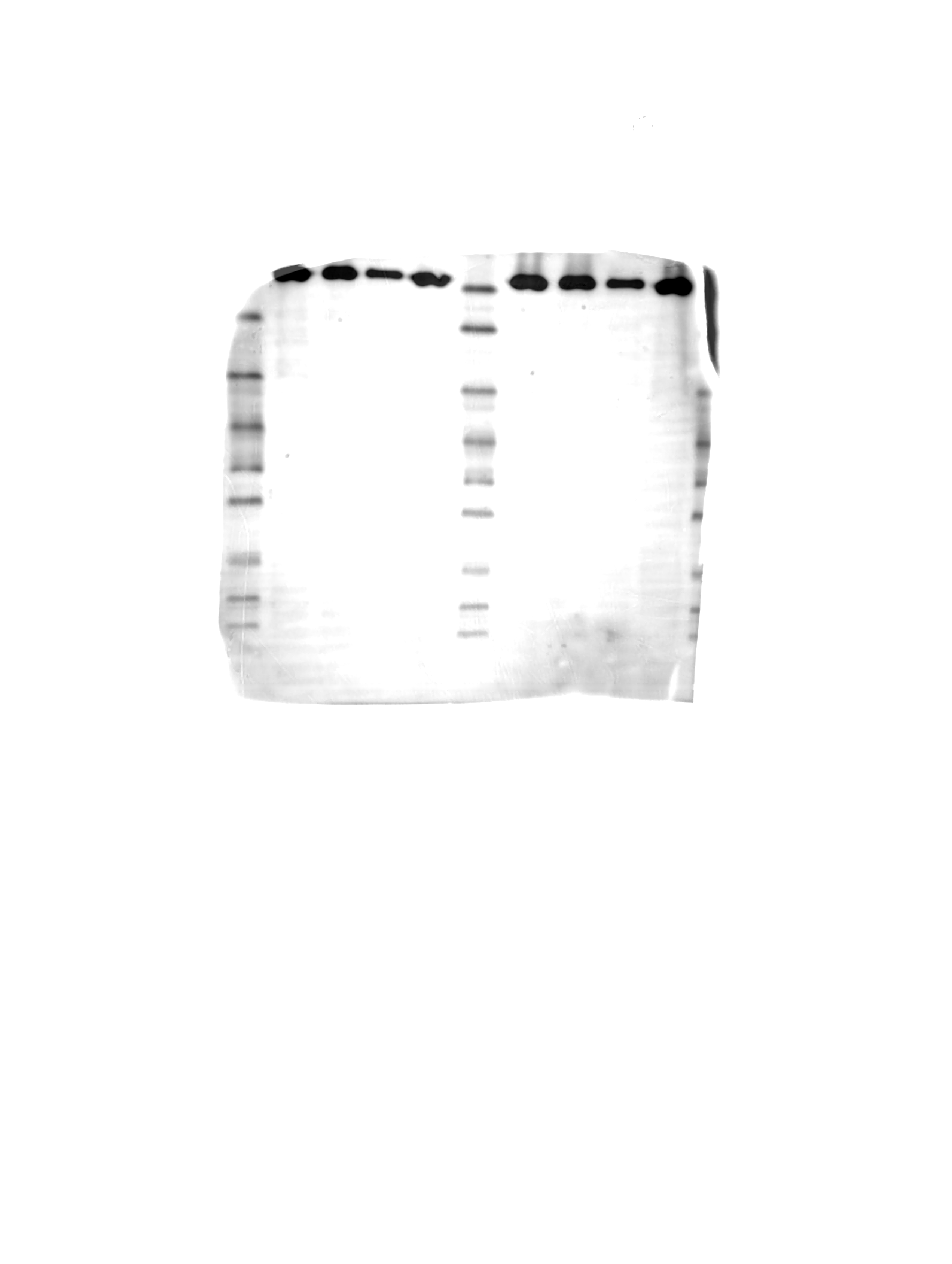

Supplement: Supplementary file 3 [file DataSheet4.zip › Figure5 wb/Figure 5 col2a1.tif]

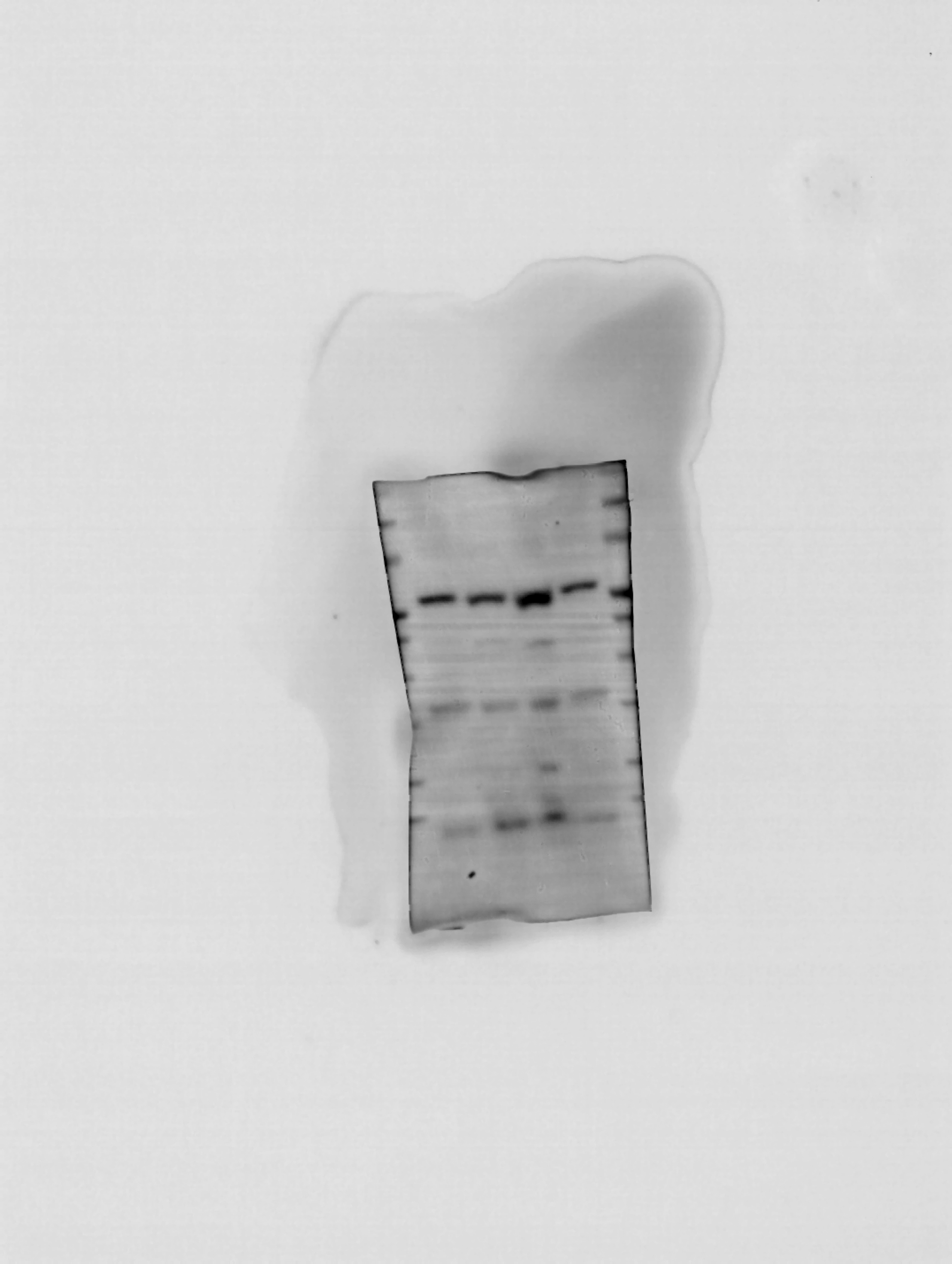

Supplement: Supplementary file 3 [file DataSheet4.zip › Figure5 wb/figure 5 B ADAMTs5 2.tif]

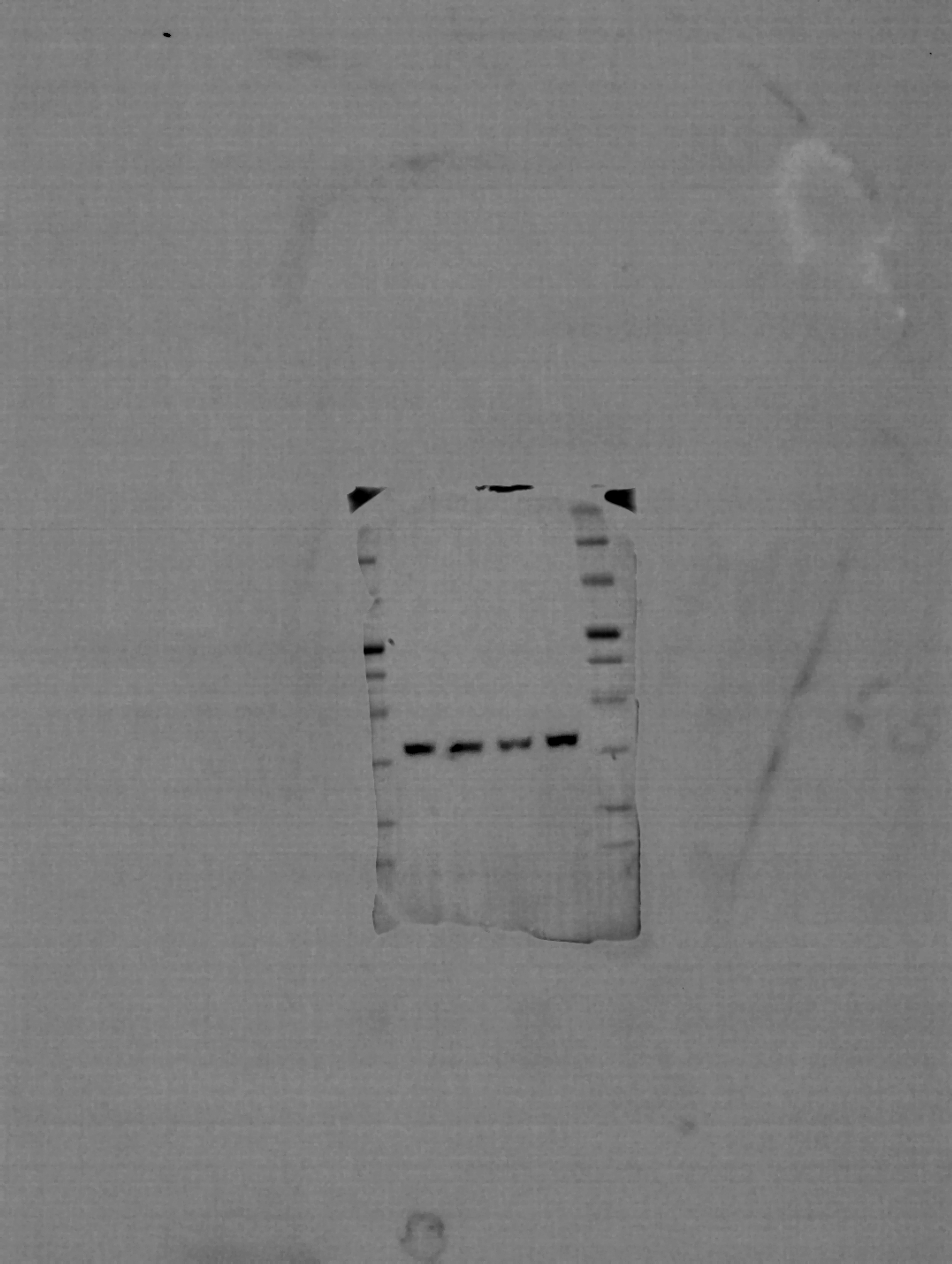

Supplement: Supplementary file 3 [file DataSheet4.zip › Figure5 wb/figure 5 B gapdh 2.tif]

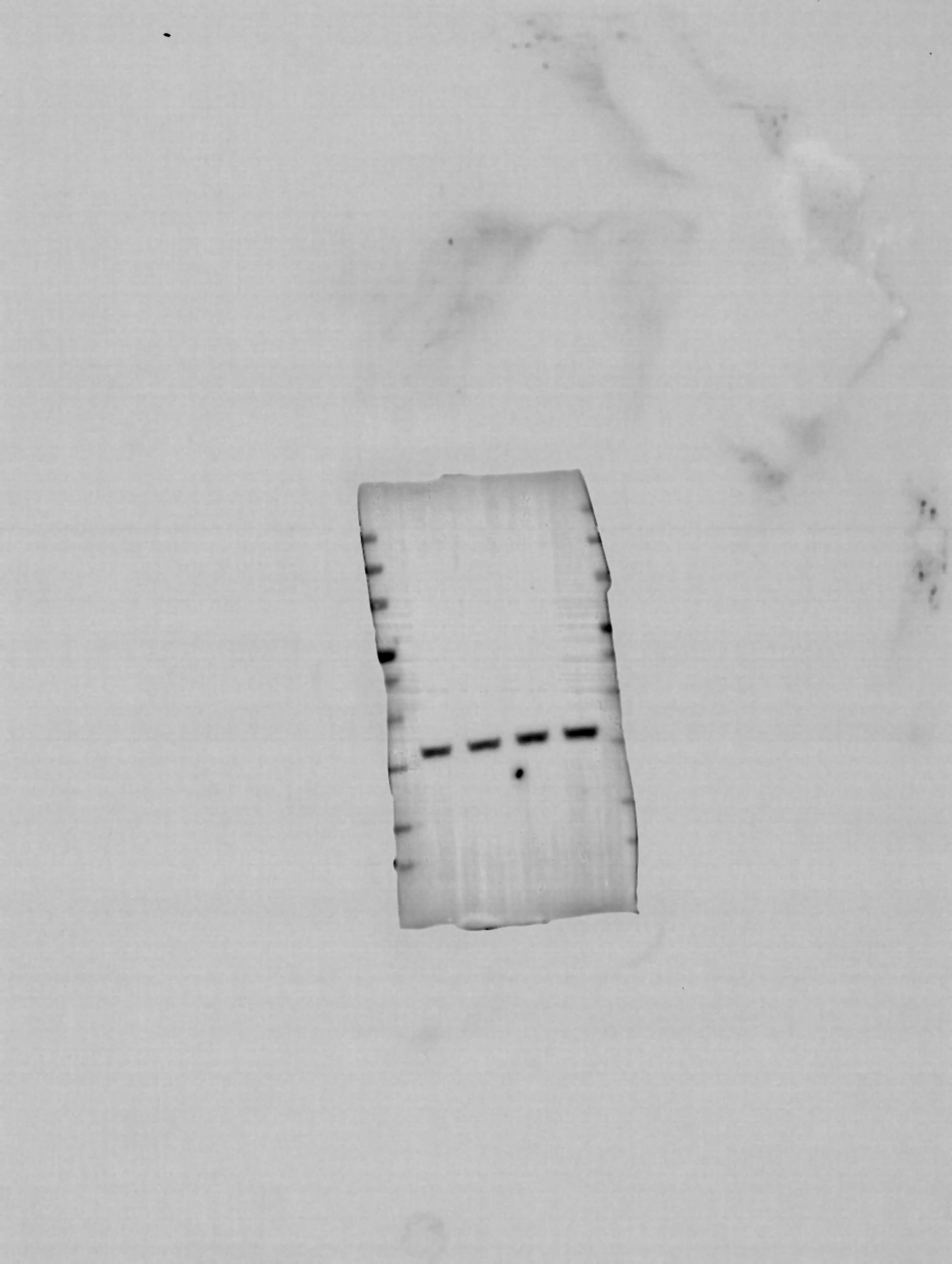

Supplement: Supplementary file 3 [file DataSheet4.zip › Figure5 wb/figure 5 B gapdh 3.tif]

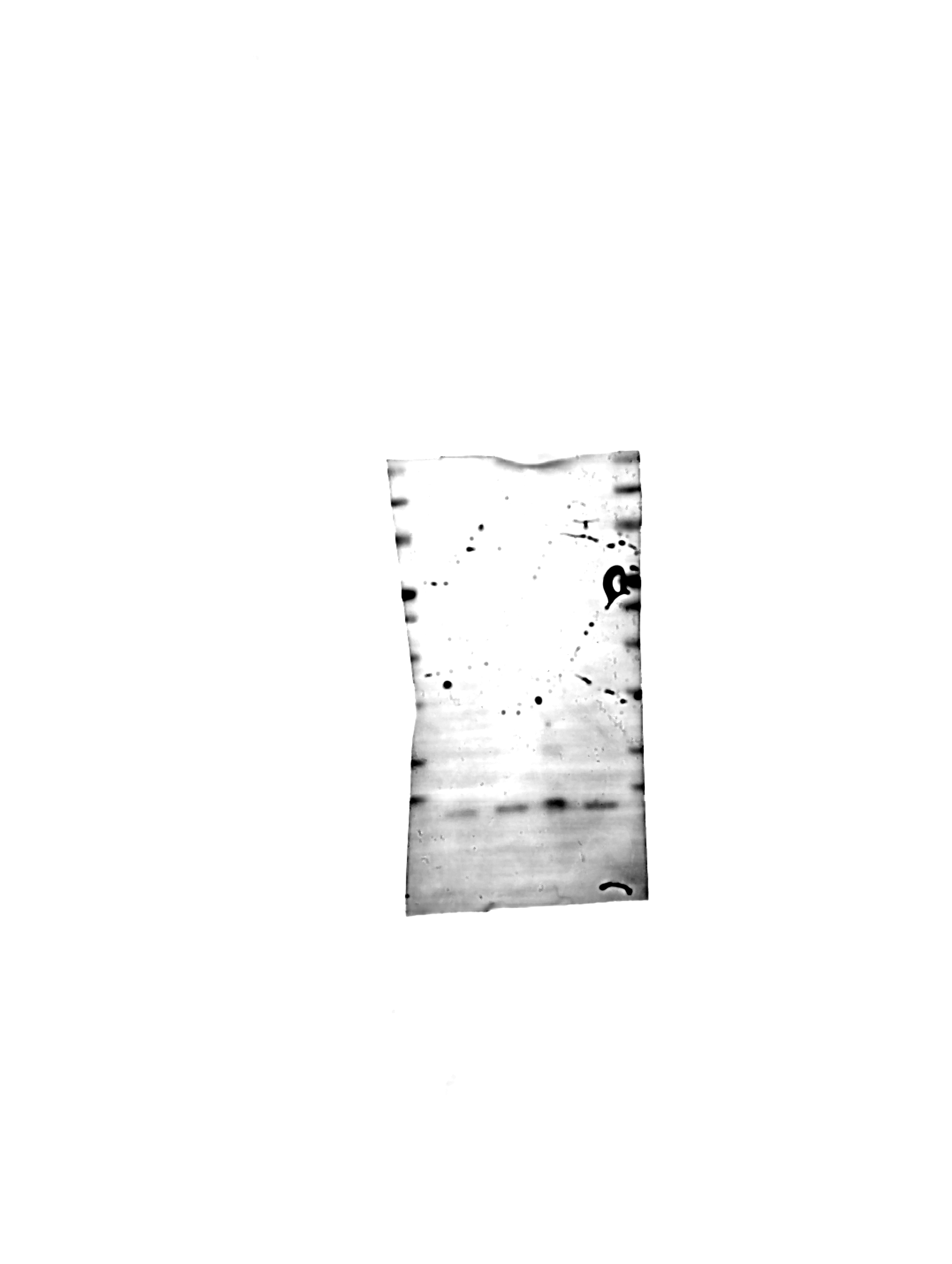

Supplement: Supplementary file 3 [file DataSheet4.zip › Figure5 wb/figure 5 B p21 2.tif]

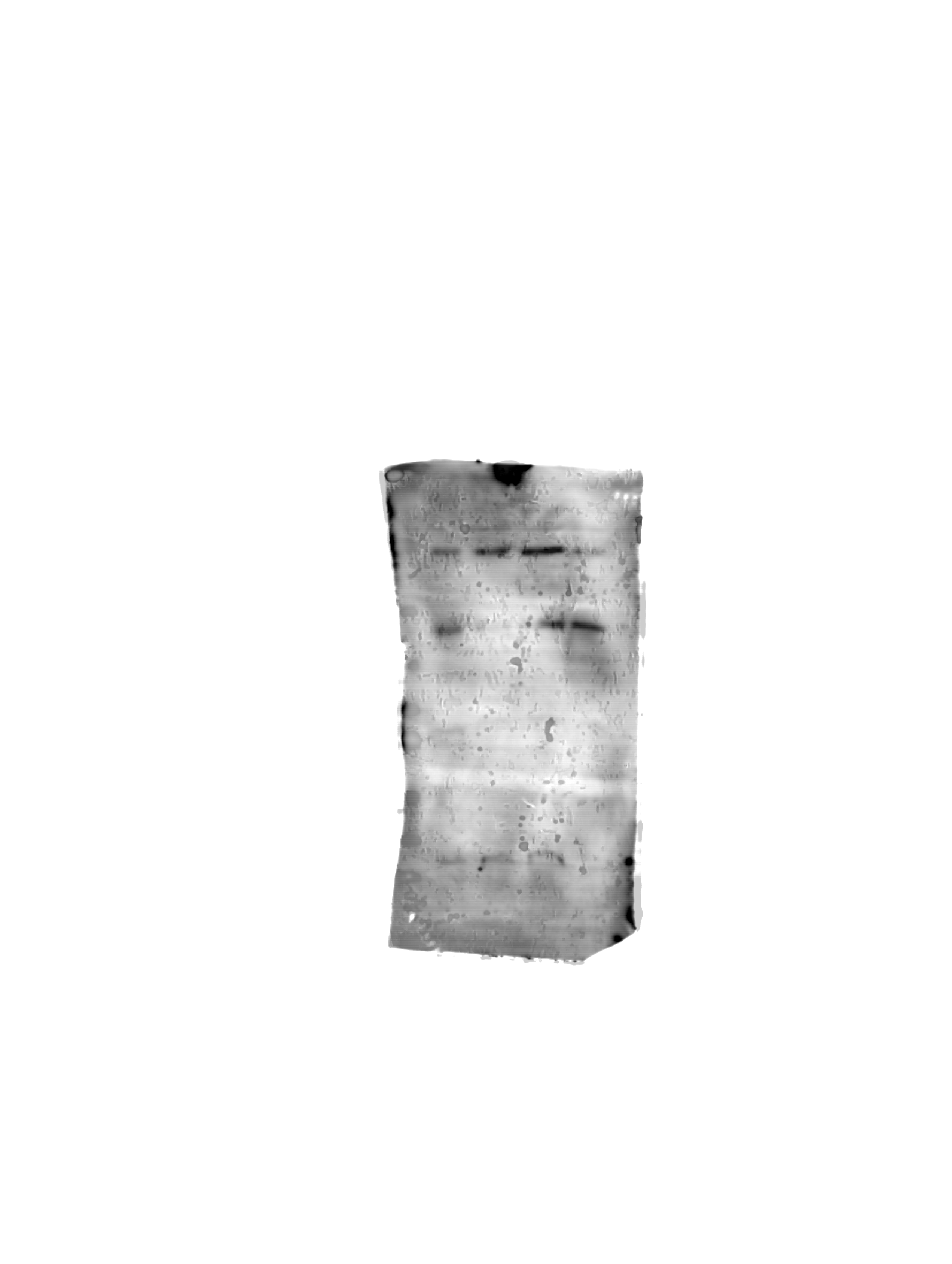

Supplement: Supplementary file 3 [file DataSheet4.zip › Figure5 wb/figure 5 adamts5.tif]

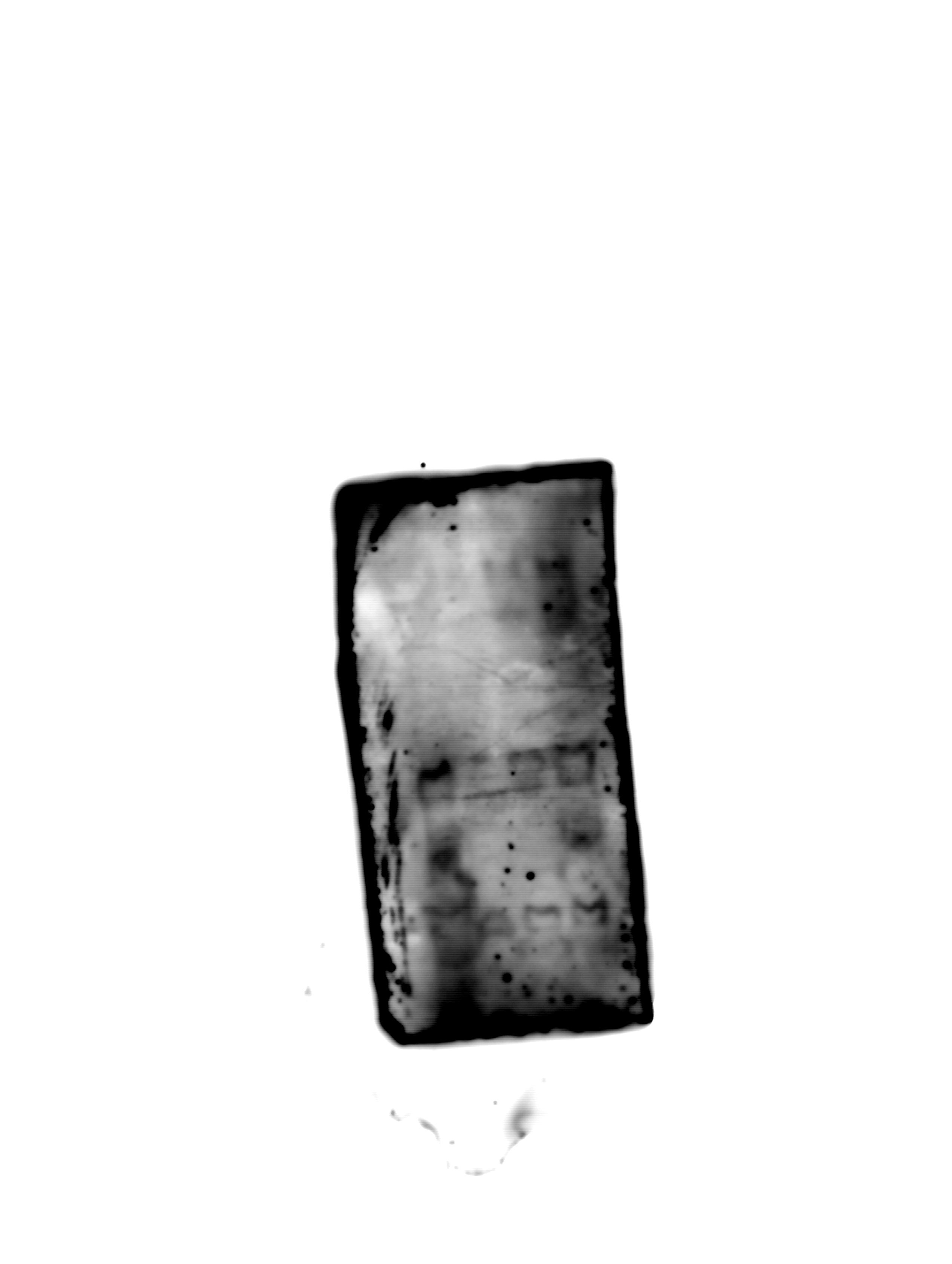

Supplement: Supplementary file 3 [file DataSheet4.zip › Figure5 wb/figure 5 col2a1 .tif]

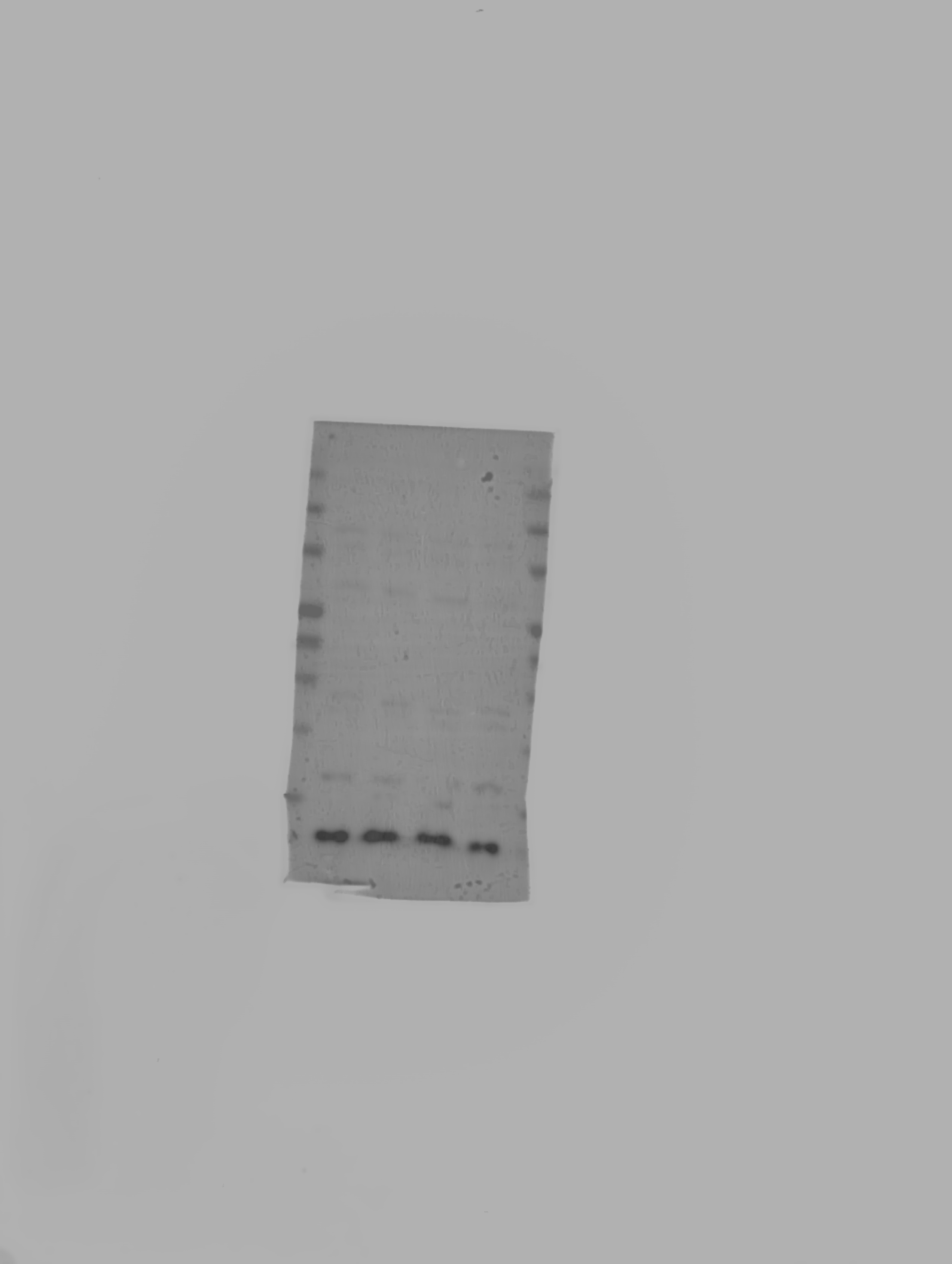

Supplement: Supplementary file 3 [file DataSheet4.zip › Figure5 wb/figure 5 p21.tif]

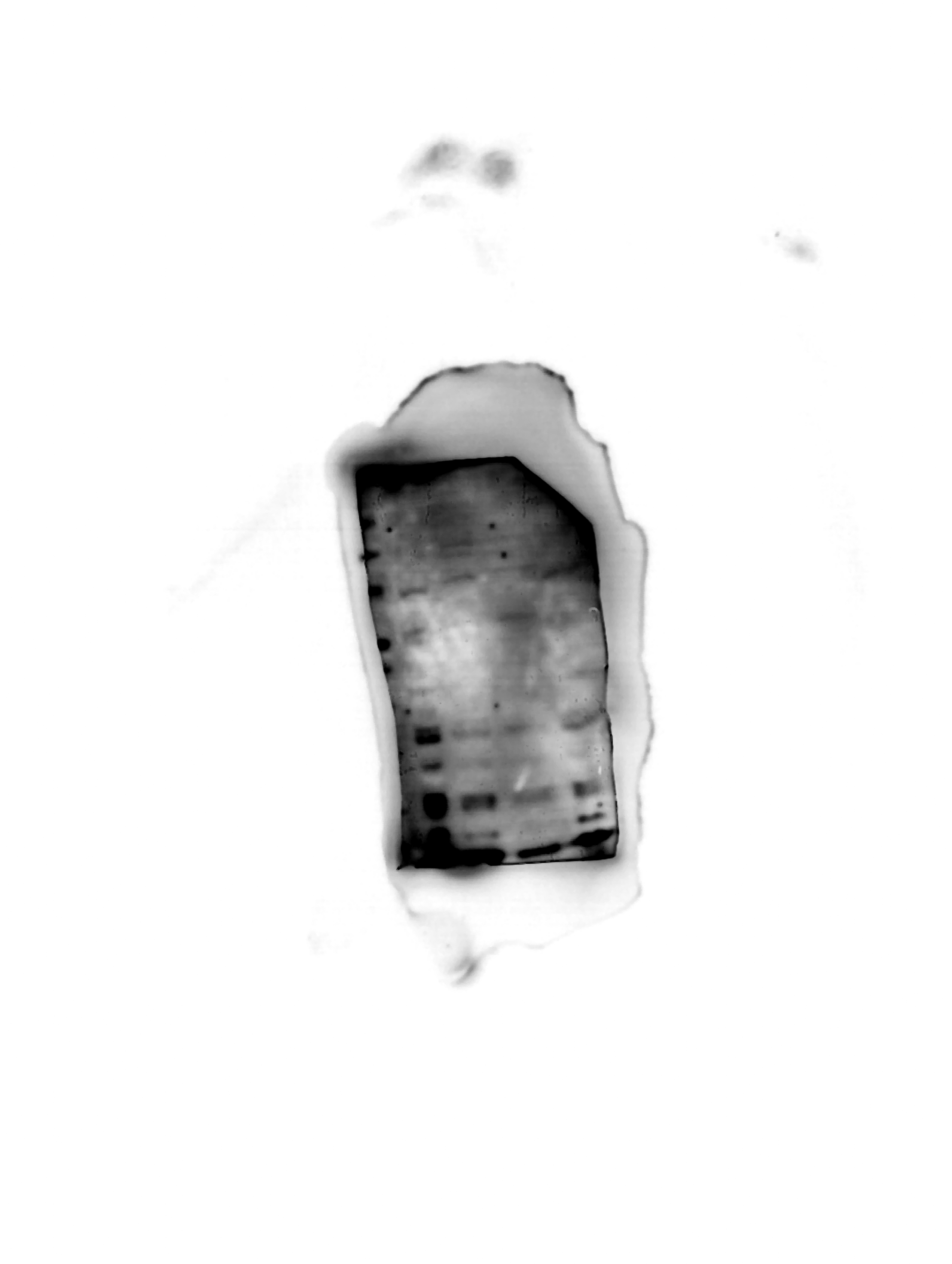

Supplement: Supplementary file 3 [file DataSheet4.zip › Figure5 wb/figure5 B col2a1 2.tif]

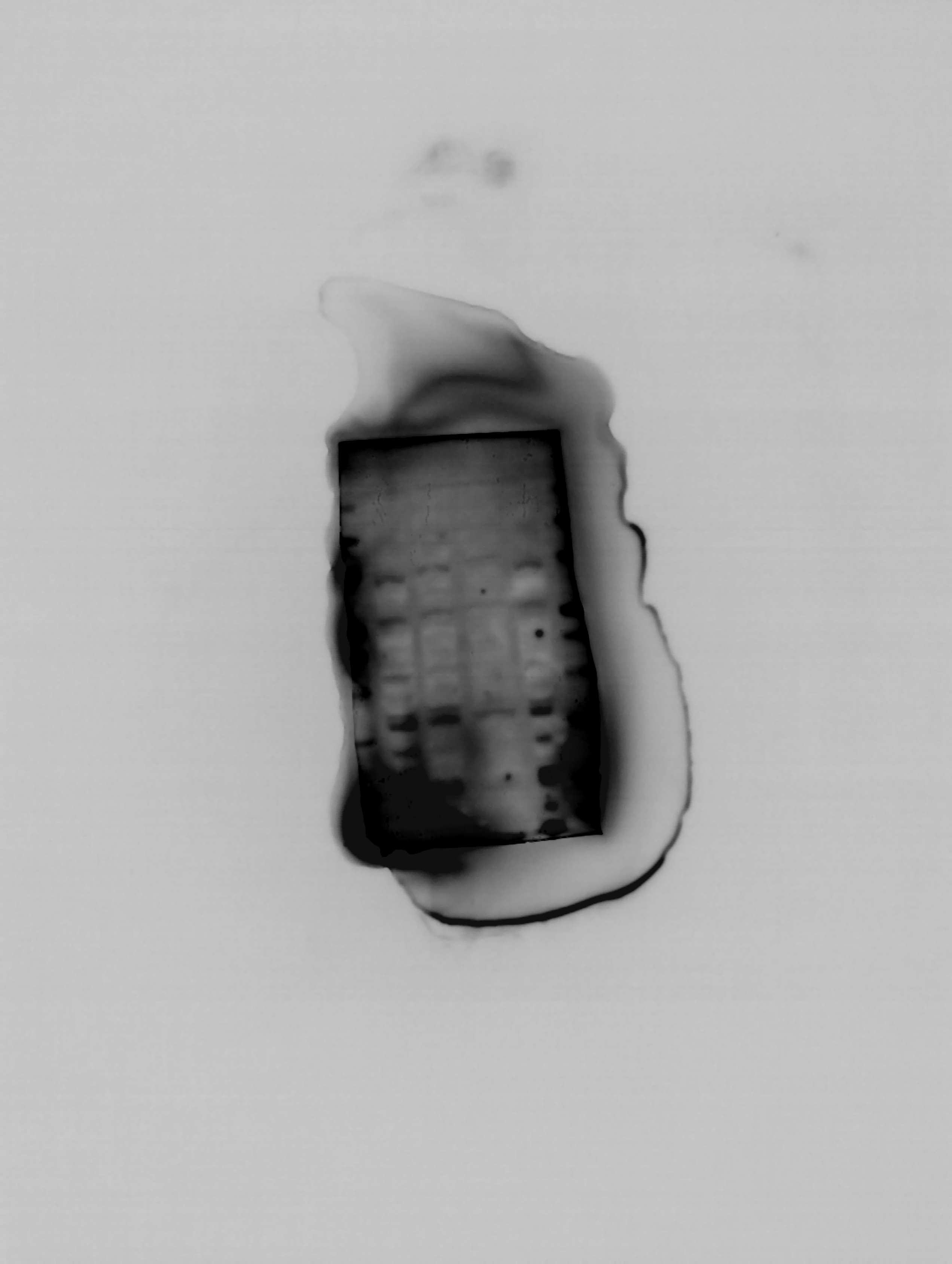

Supplement: Supplementary file 3 [file DataSheet4.zip › Figure5 wb/figure5 B col2a1 3.tif]

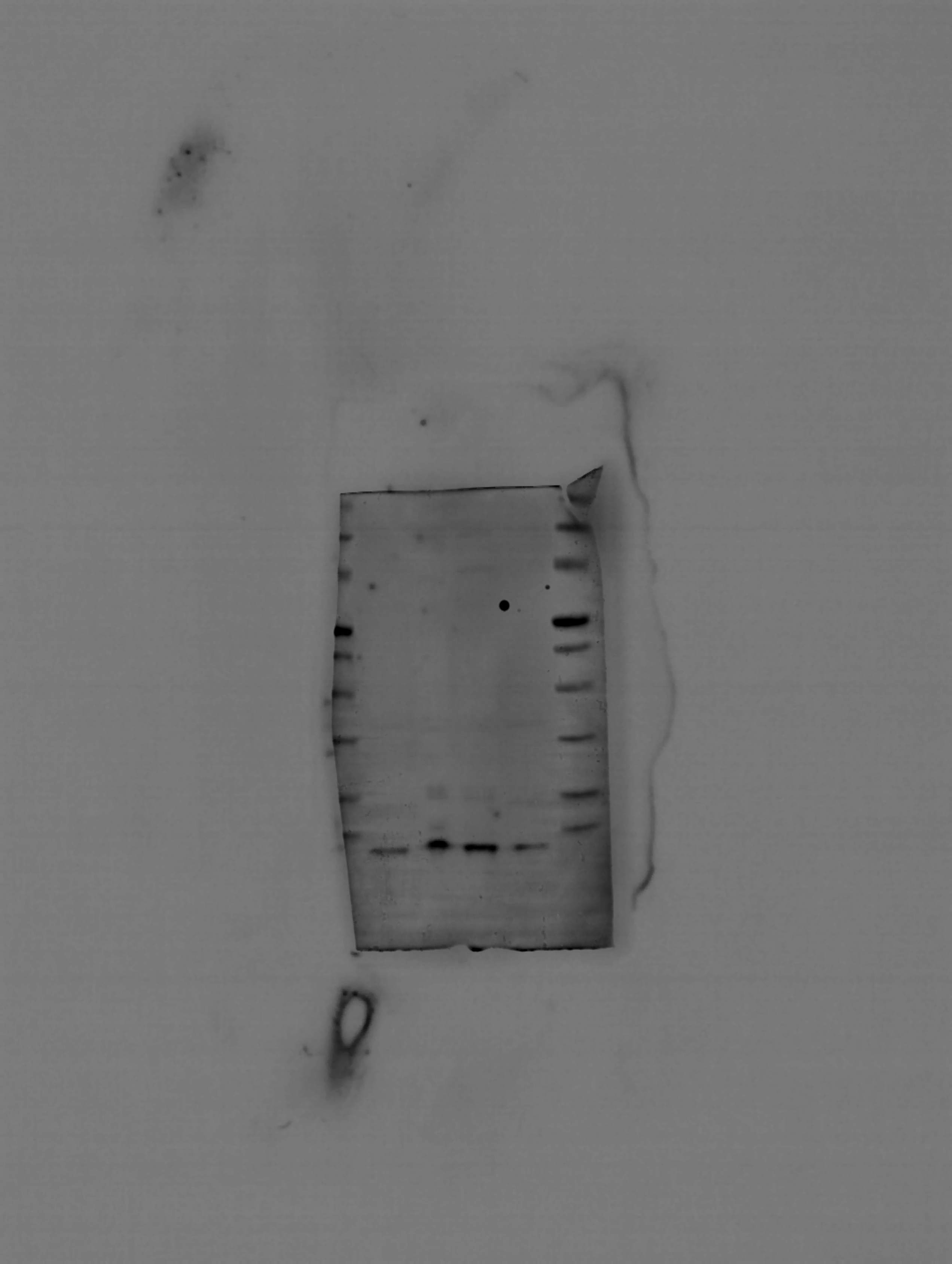

Supplement: Supplementary file 3 [file DataSheet4.zip › Figure5 wb/figure5 B p21 3.tif]

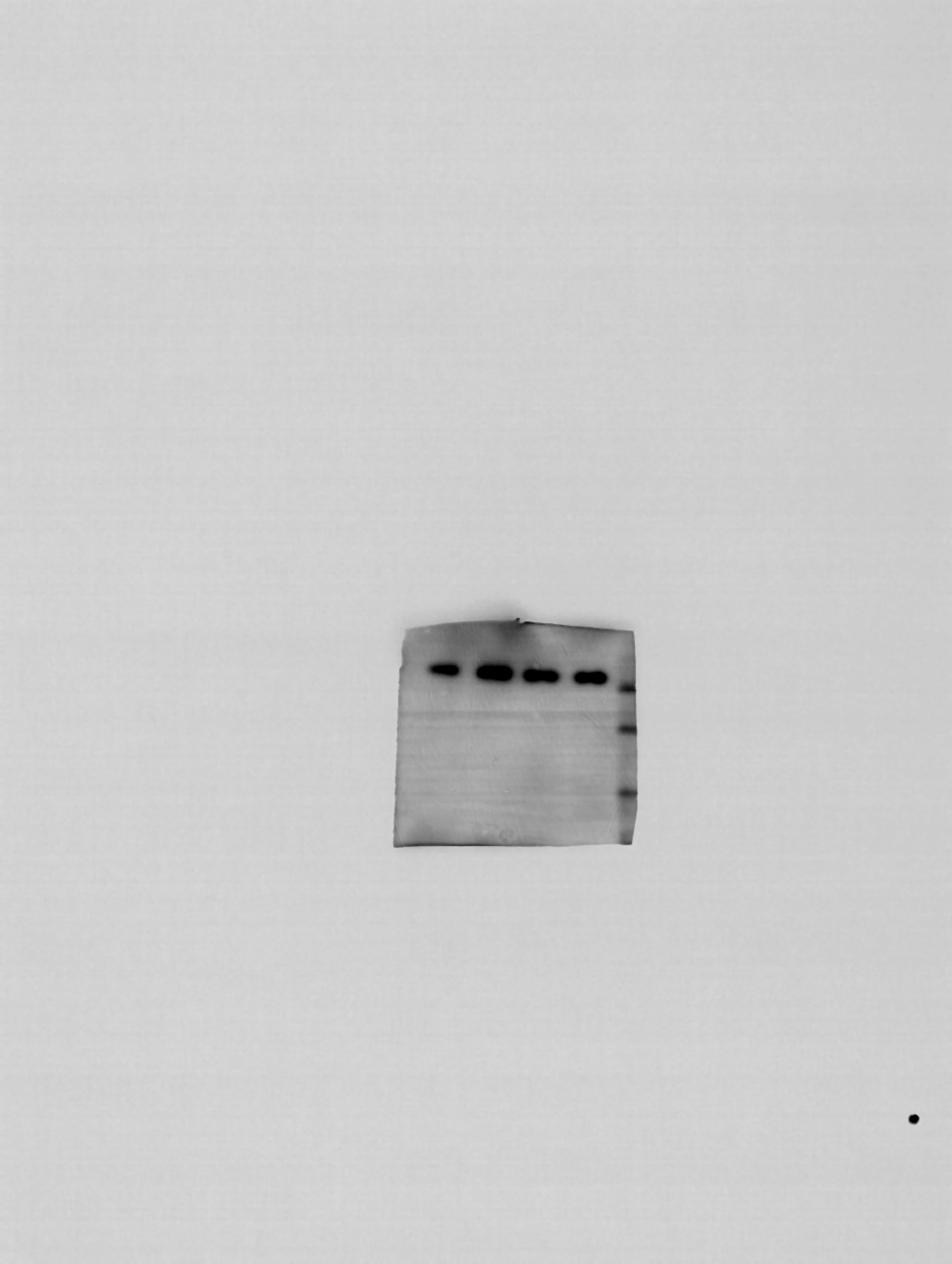

Supplement: Supplementary file 3 [file DataSheet4.zip › Figure5 wb/gapdh 11.tif]

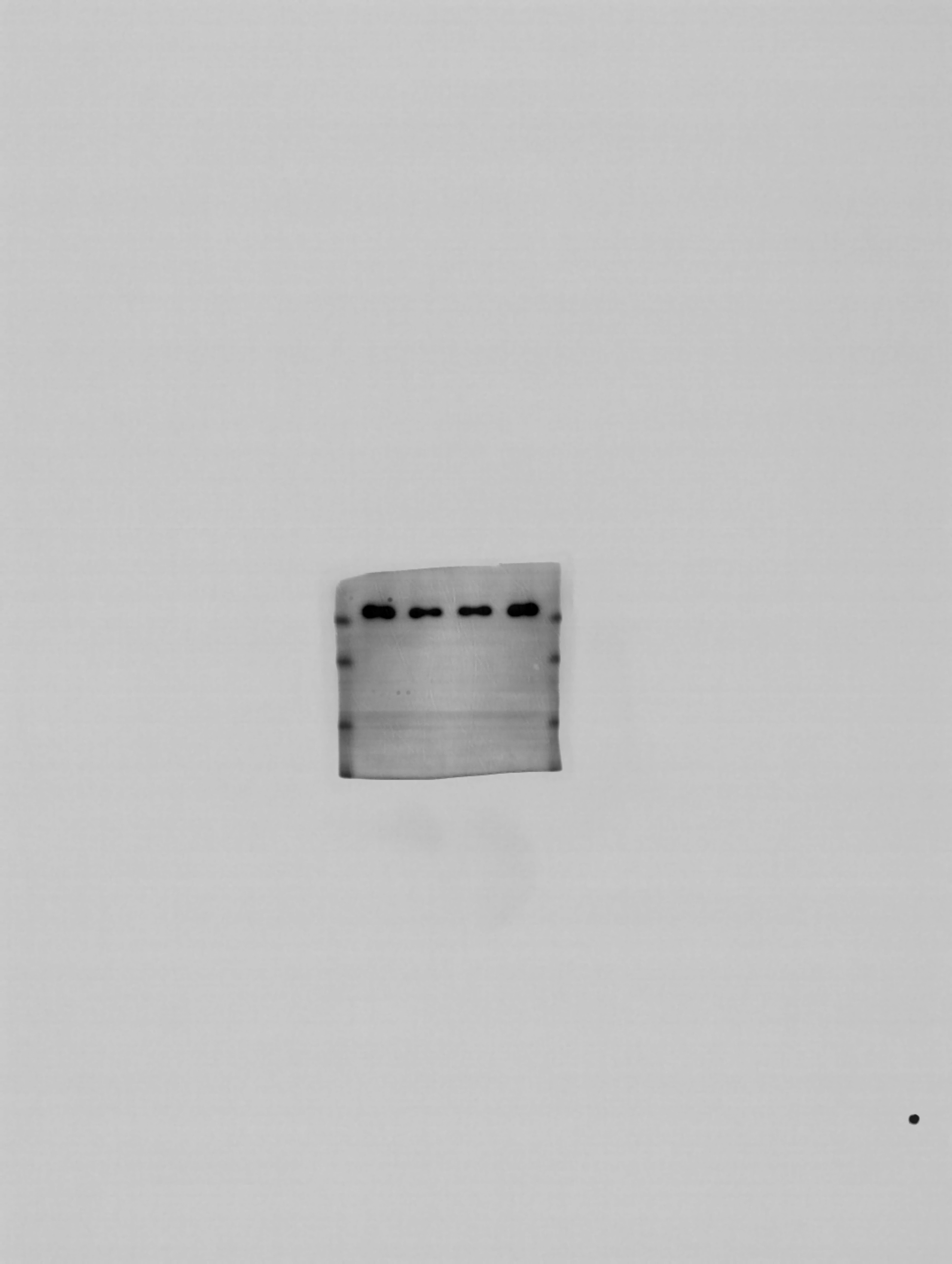

Supplement: Supplementary file 3 [file DataSheet4.zip › Figure5 wb/gapdh 12.tif]

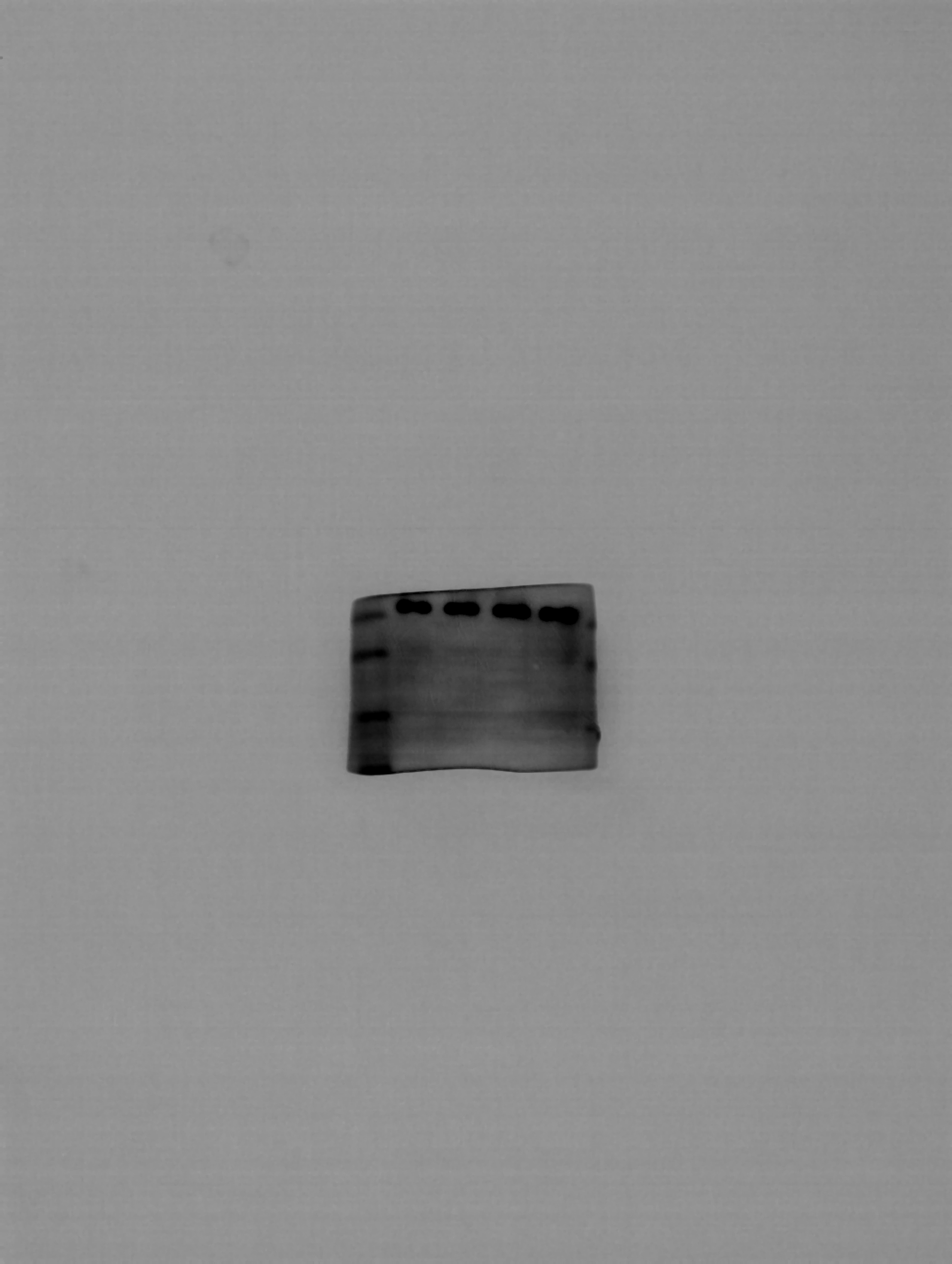

Supplement: Supplementary file 3 [file DataSheet4.zip › Figure5 wb/gapdh 13.tif]

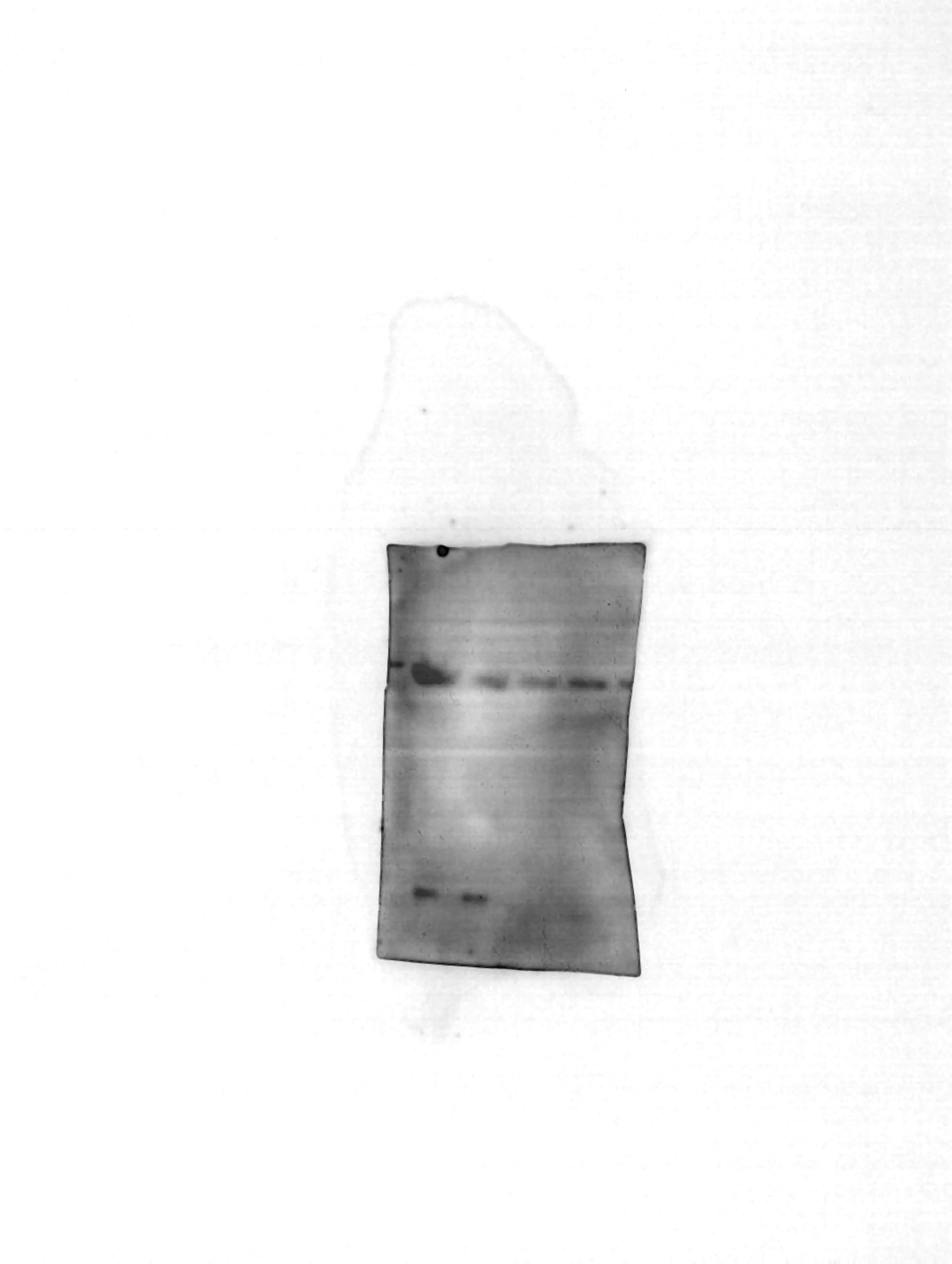

Supplement: Supplementary file 4 [file DataSheet1.zip › Figure1 wb/Figure 1 B dlat 2.tif]

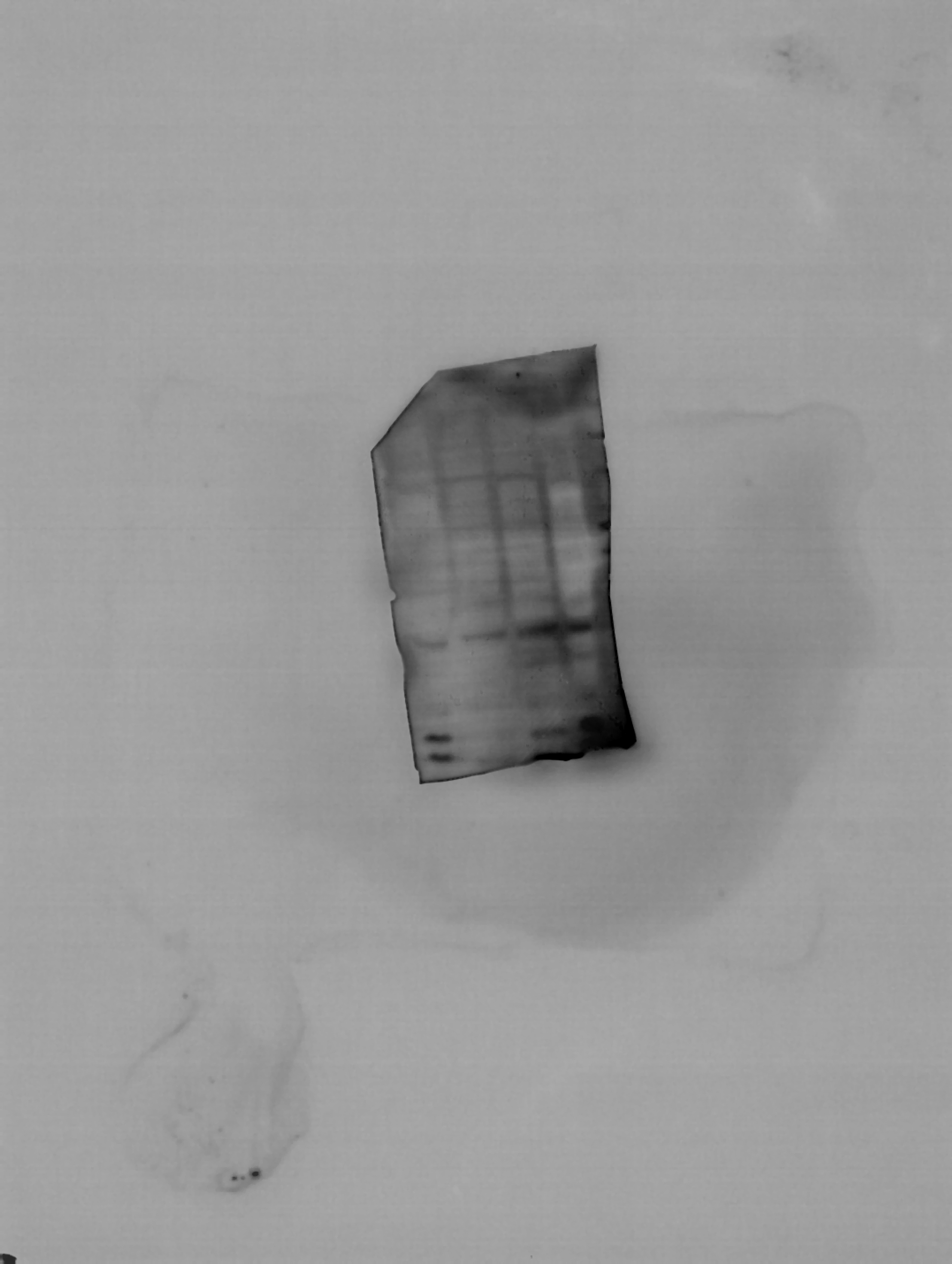

Supplement: Supplementary file 4 [file DataSheet1.zip › Figure1 wb/figure 1 B fdx1 2.tif]

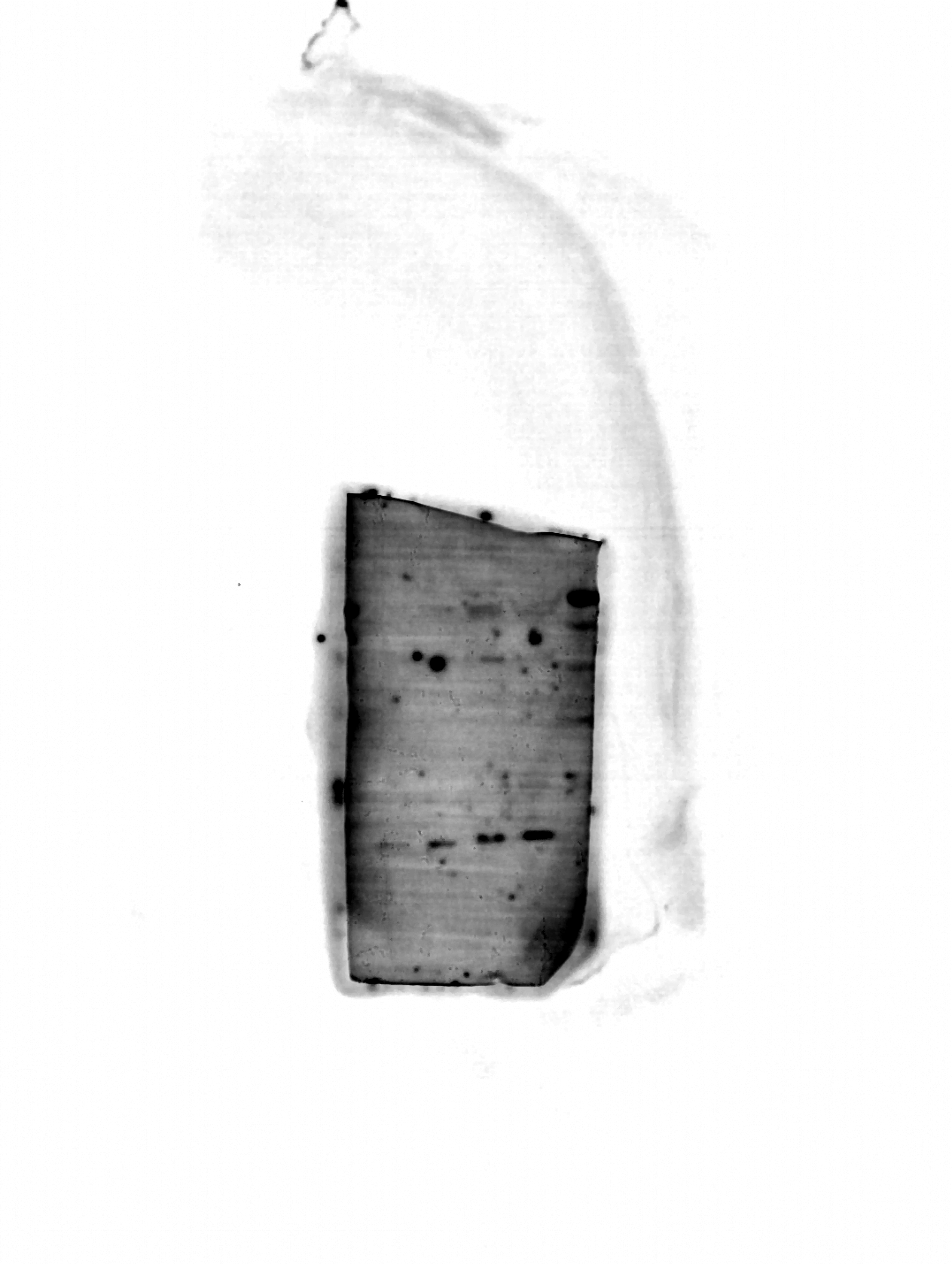

Supplement: Supplementary file 4 [file DataSheet1.zip › Figure1 wb/figure 1 B fdx1 3.tif]

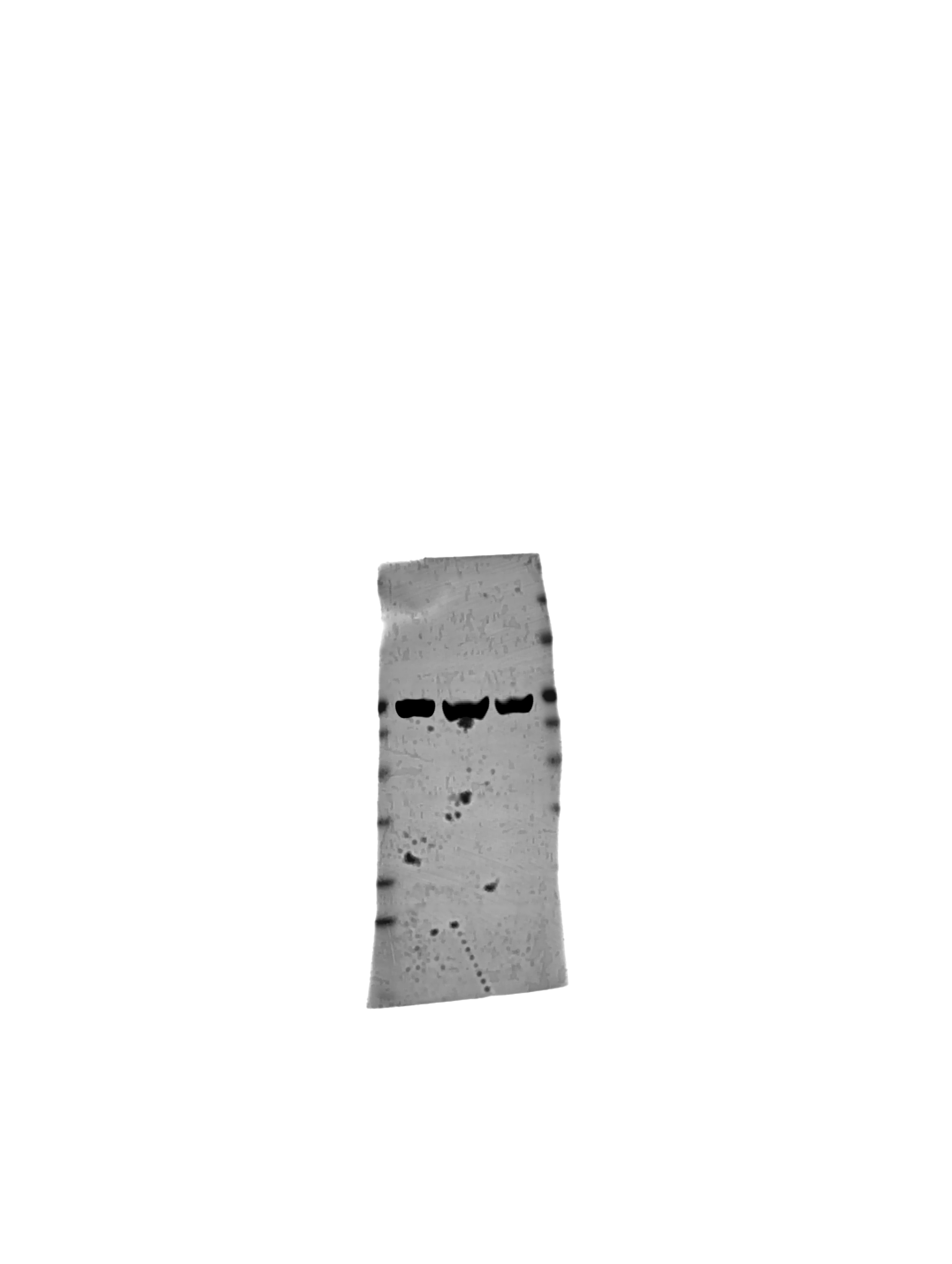

Supplement: Supplementary file 4 [file DataSheet1.zip › Figure1 wb/figure 1 C dlat 3.tif]

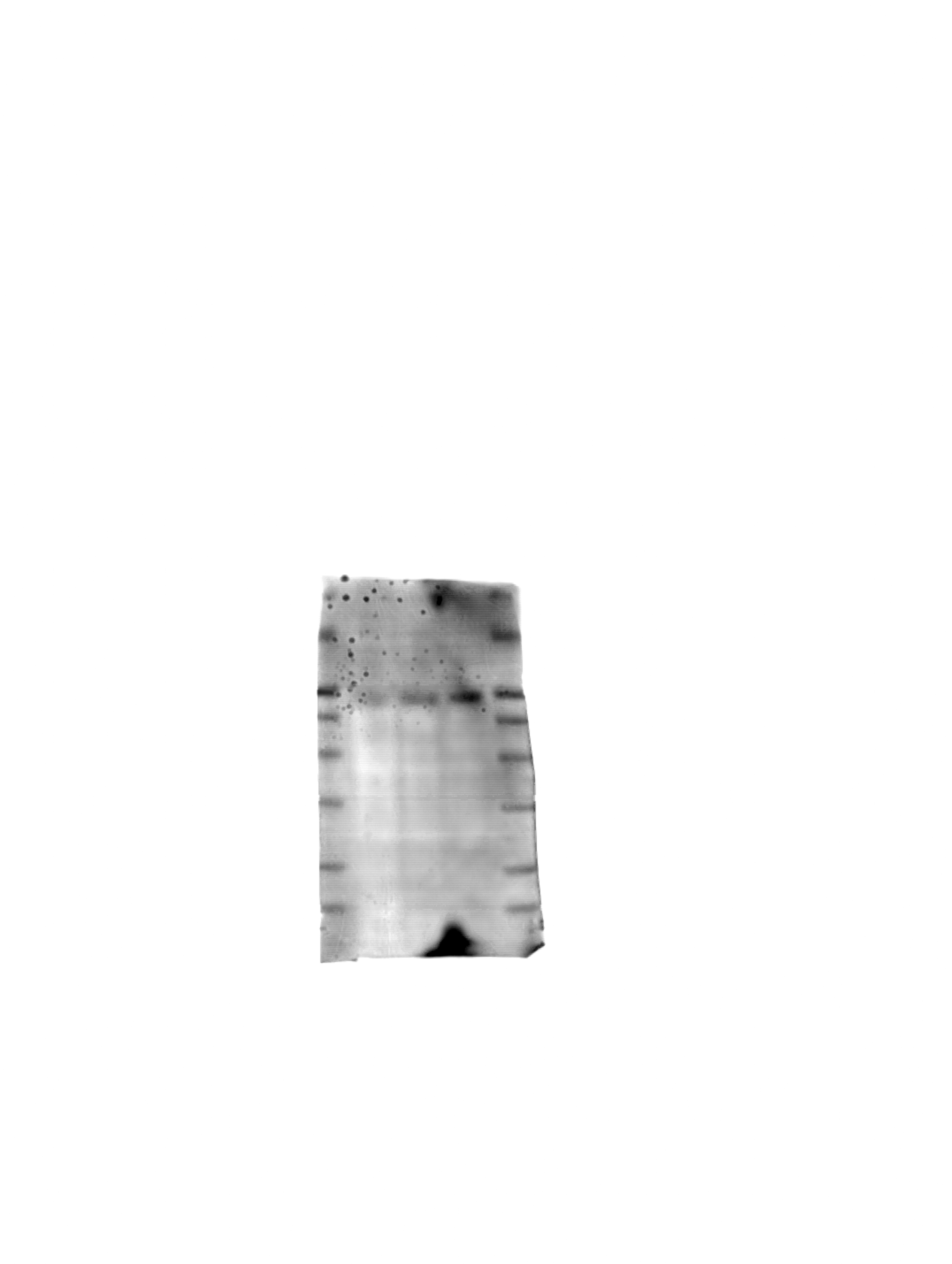

Supplement: Supplementary file 4 [file DataSheet1.zip › Figure1 wb/figure 1 C dlat.tif]

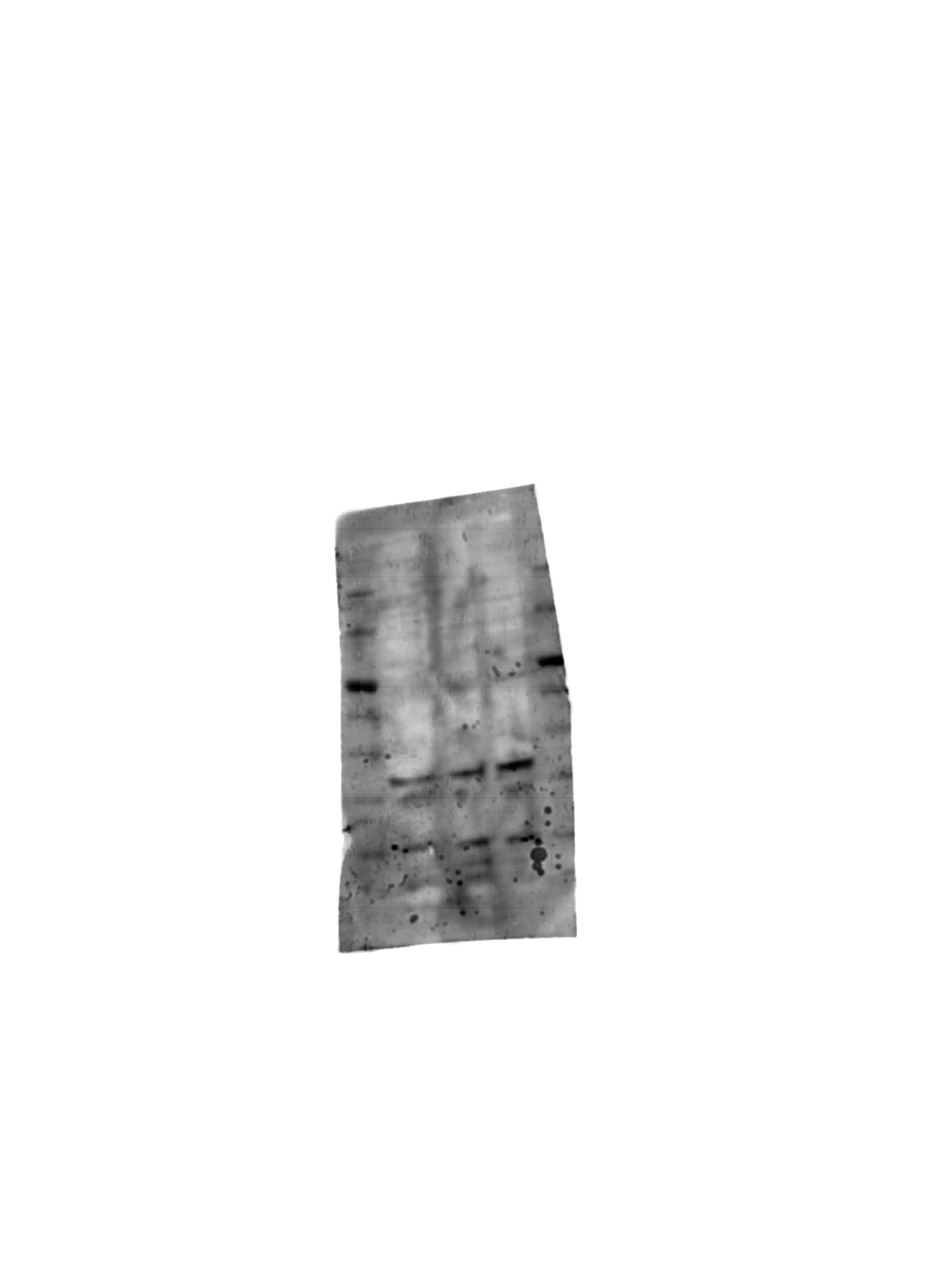

Supplement: Supplementary file 4 [file DataSheet1.zip › Figure1 wb/figure 1 C fdx1 .tif]

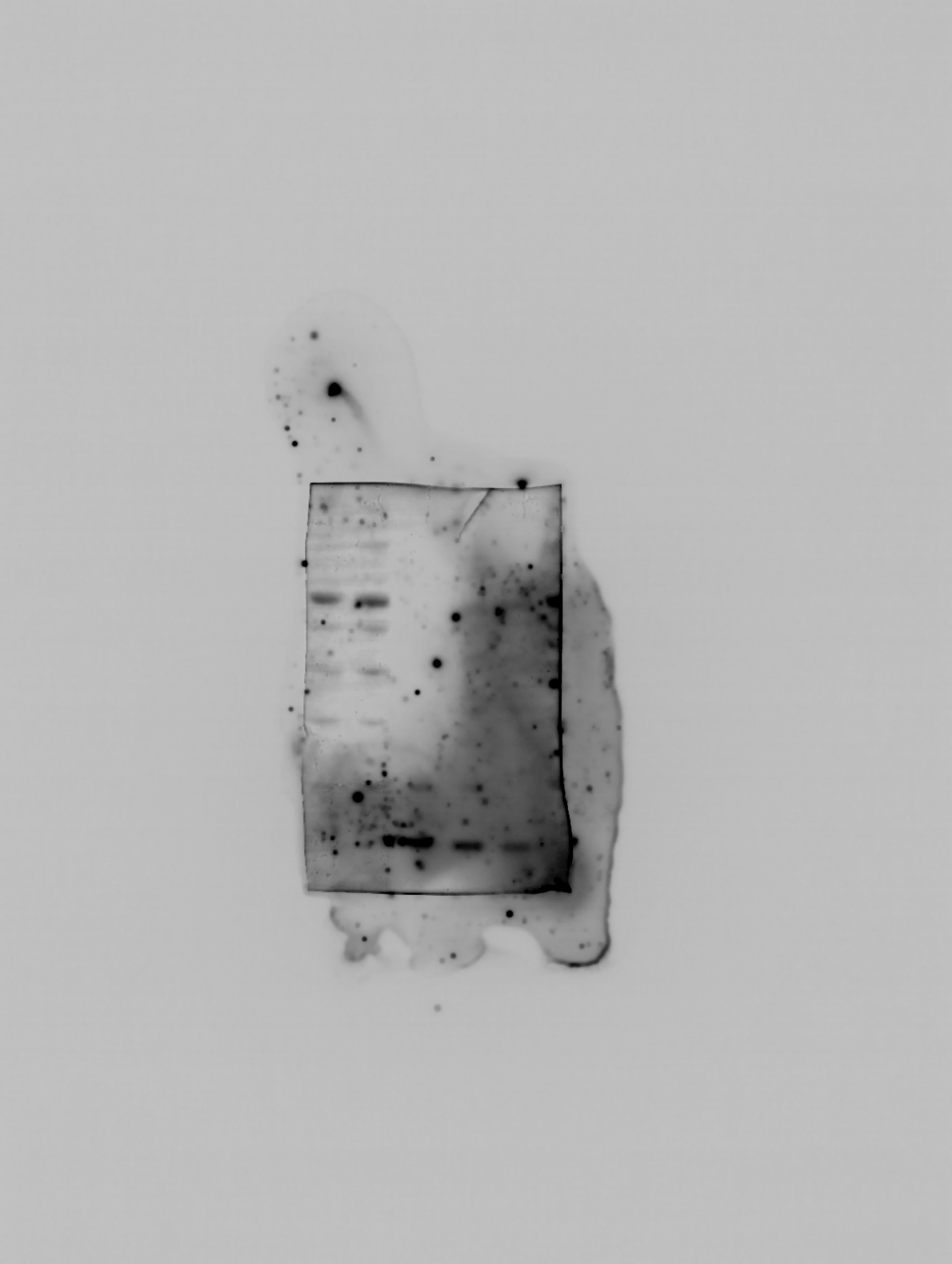

Supplement: Supplementary file 4 [file DataSheet1.zip › Figure1 wb/figure 1 C fdx1 2.tif]

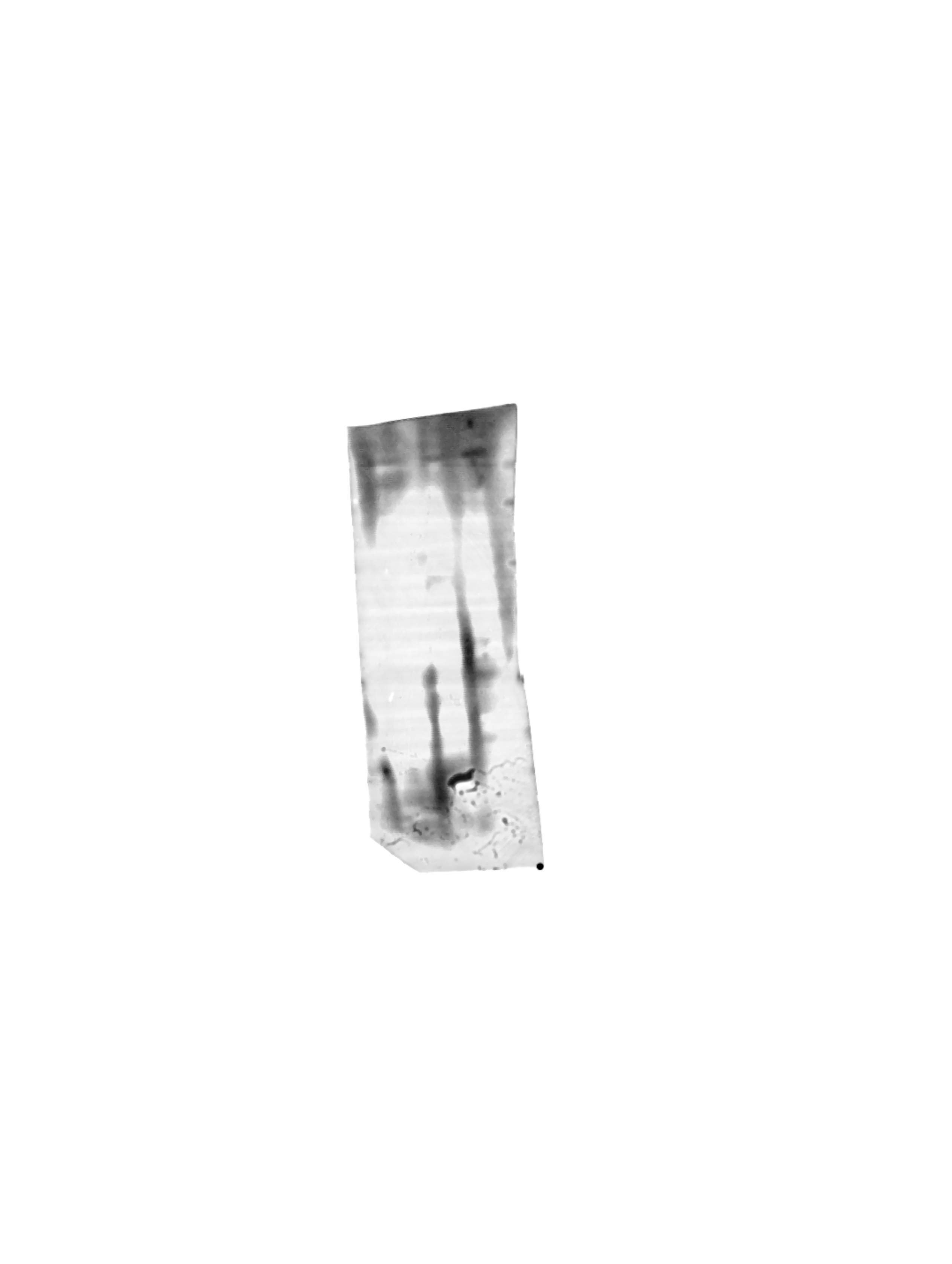

Supplement: Supplementary file 4 [file DataSheet1.zip › Figure1 wb/figure 1 C fdx1 3.tif]

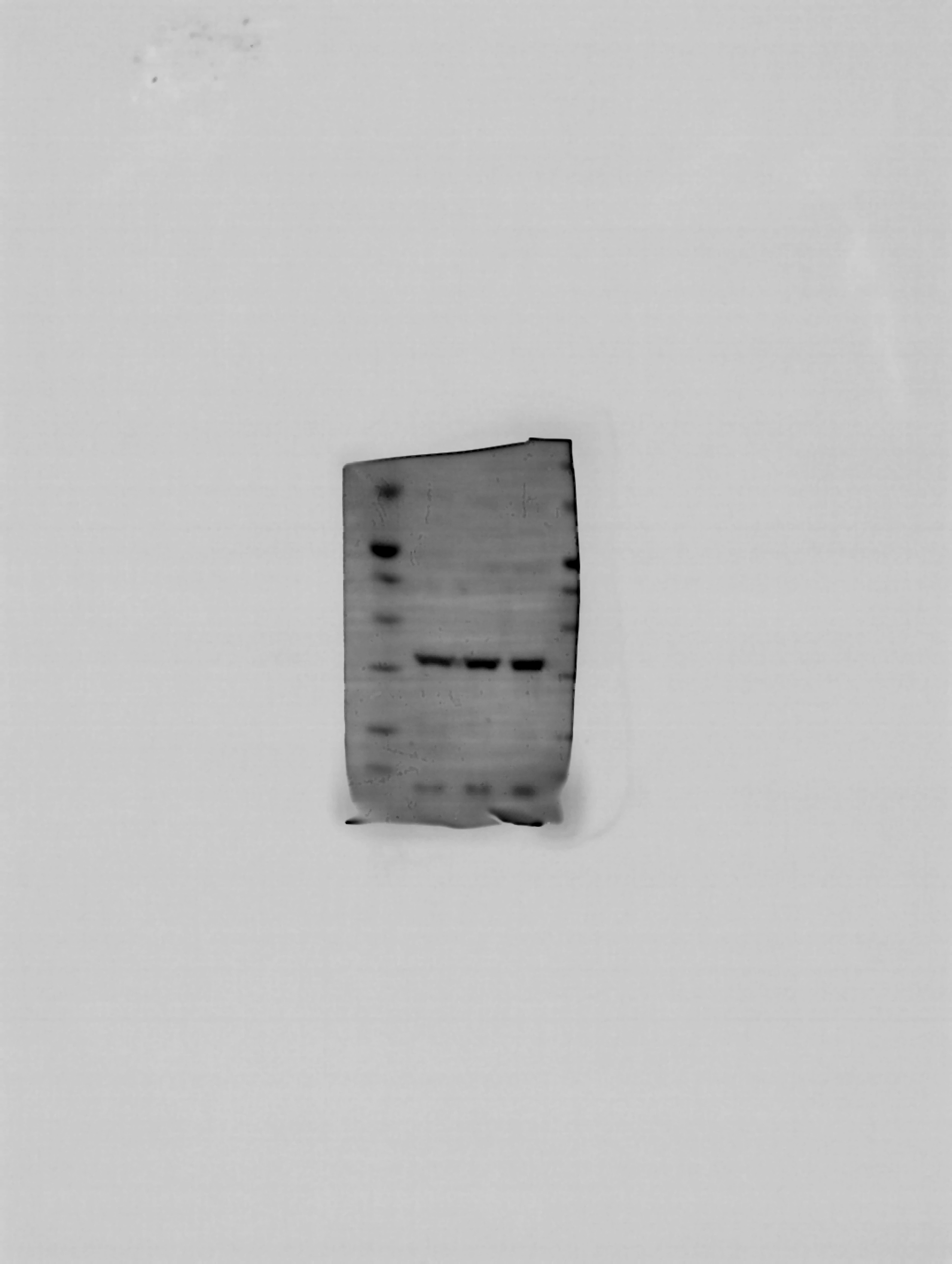

Supplement: Supplementary file 4 [file DataSheet1.zip › Figure1 wb/figure 1 C gapdh 2.tif]

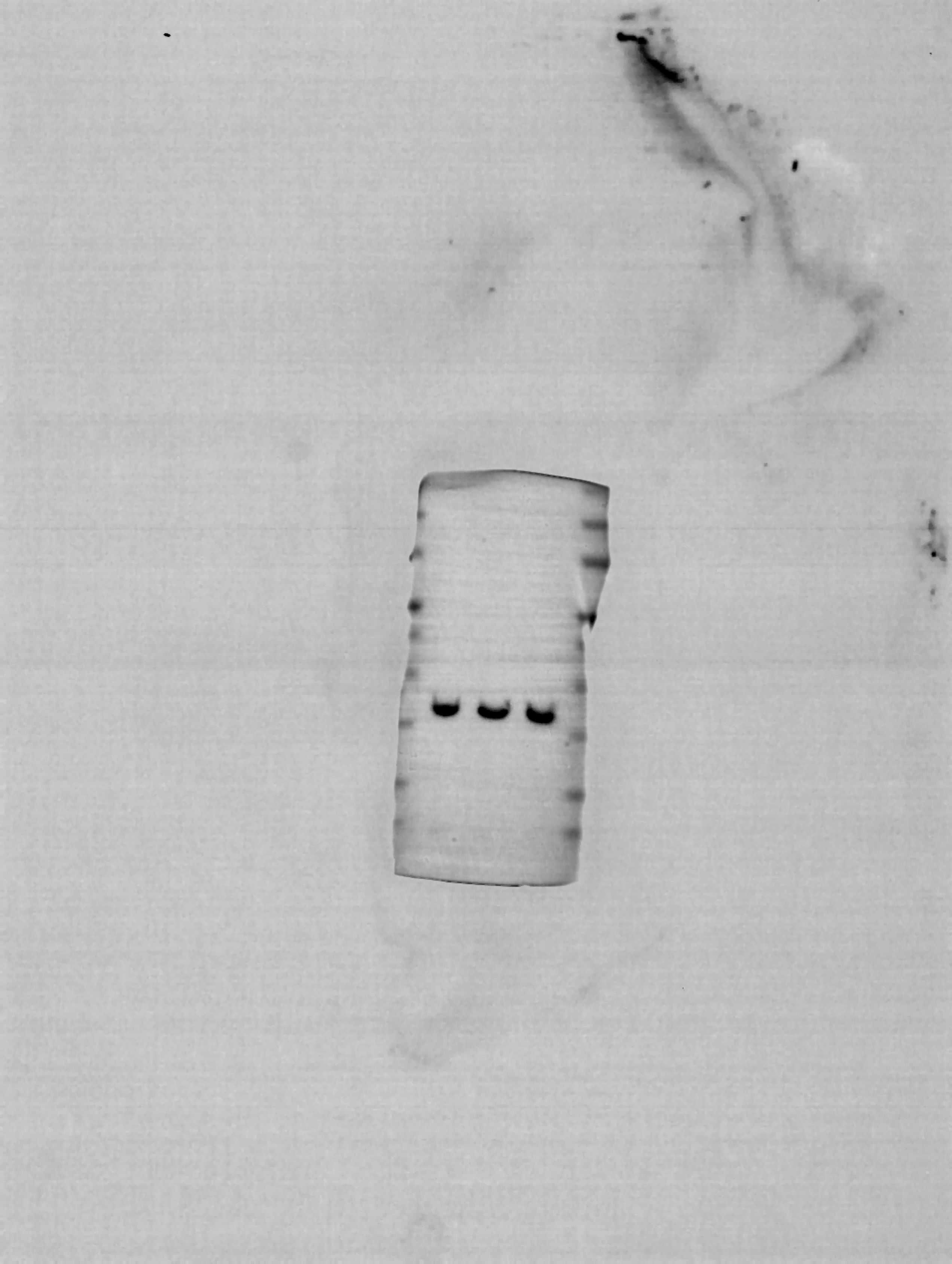

Supplement: Supplementary file 4 [file DataSheet1.zip › Figure1 wb/figure 1 C gapdh 3.tif]

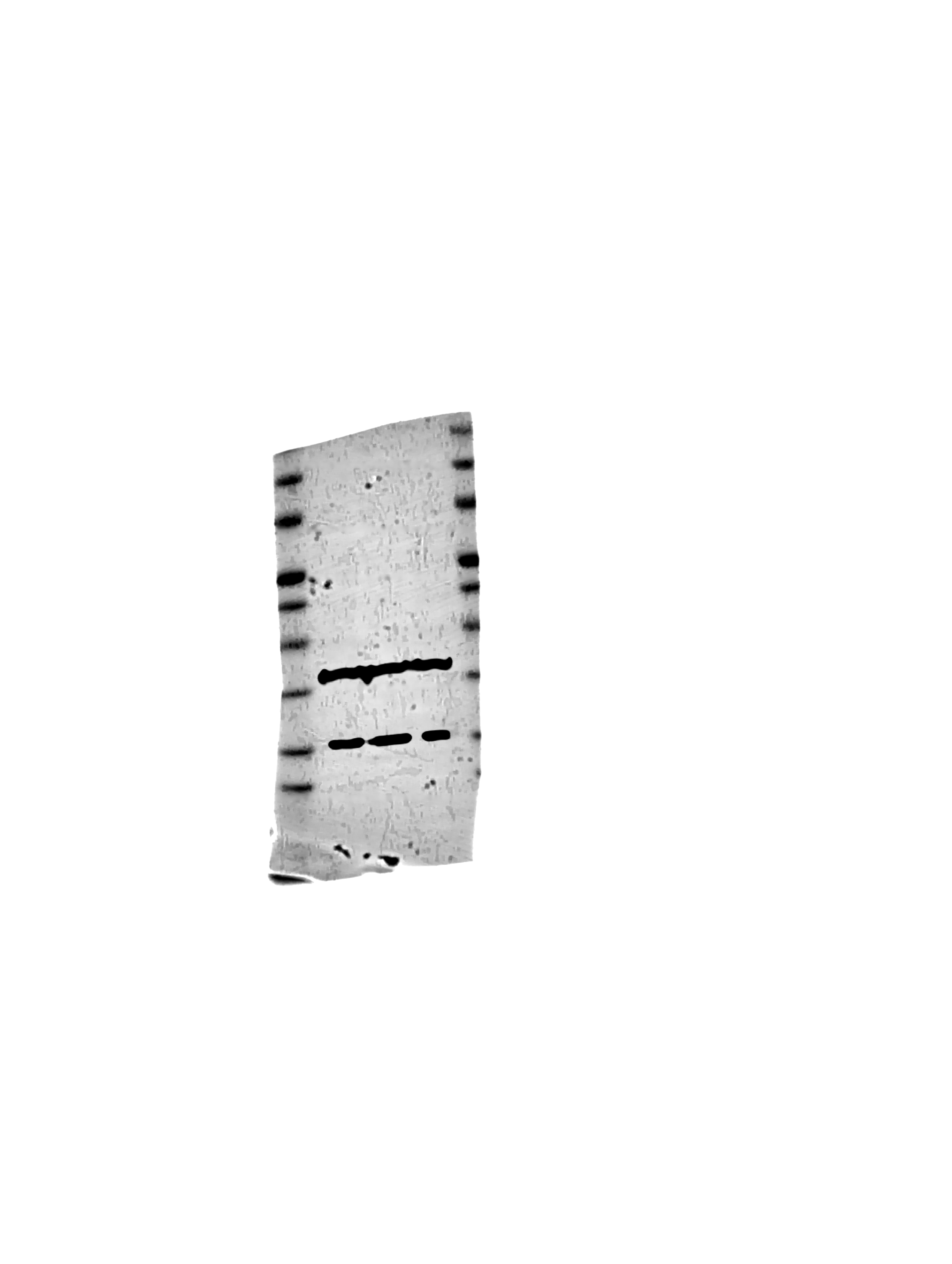

Supplement: Supplementary file 4 [file DataSheet1.zip › Figure1 wb/figure 1 C gapdh.tif]

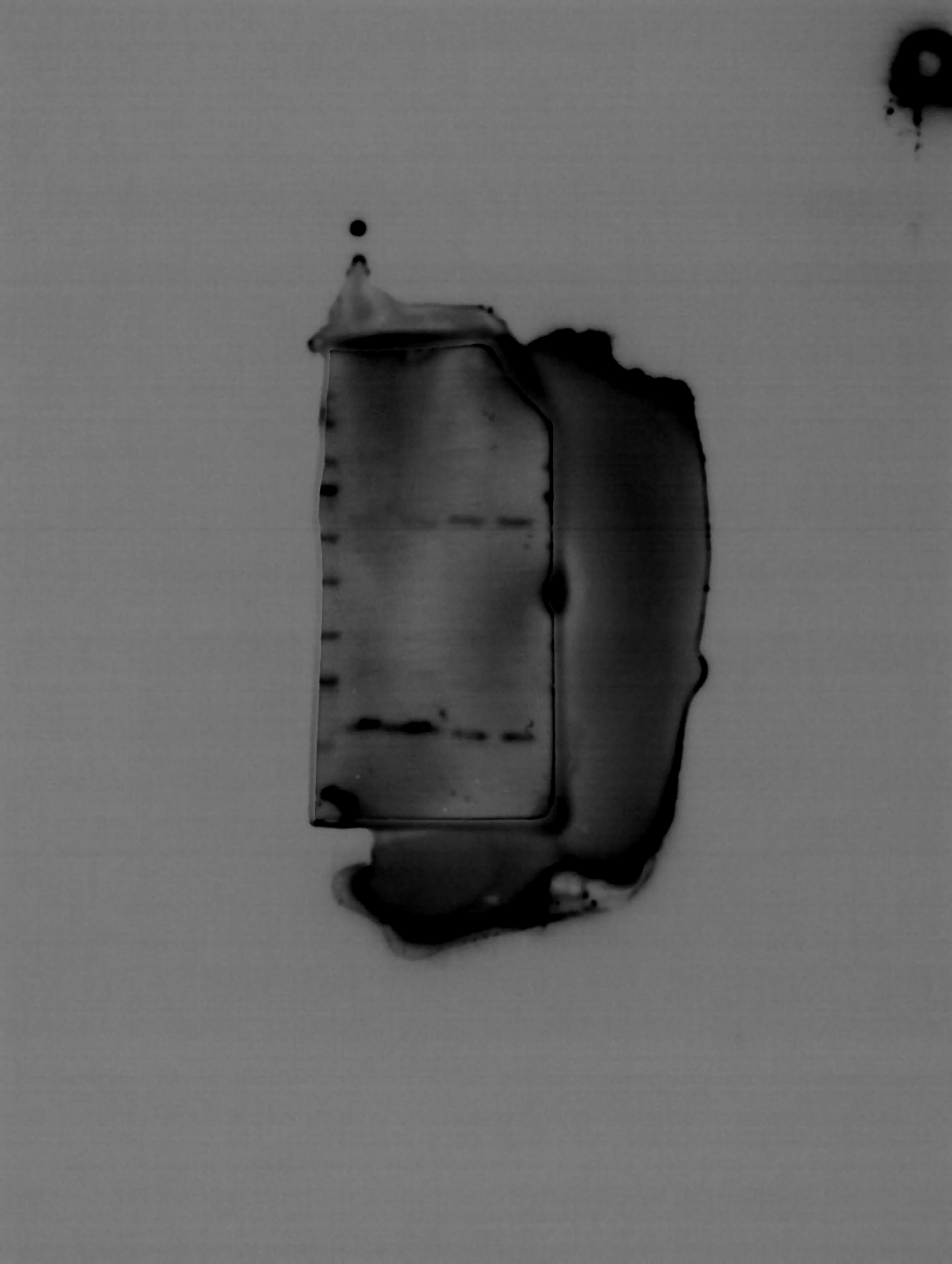

Supplement: Supplementary file 4 [file DataSheet1.zip › Figure1 wb/figure 1 D dlat 2.tif]

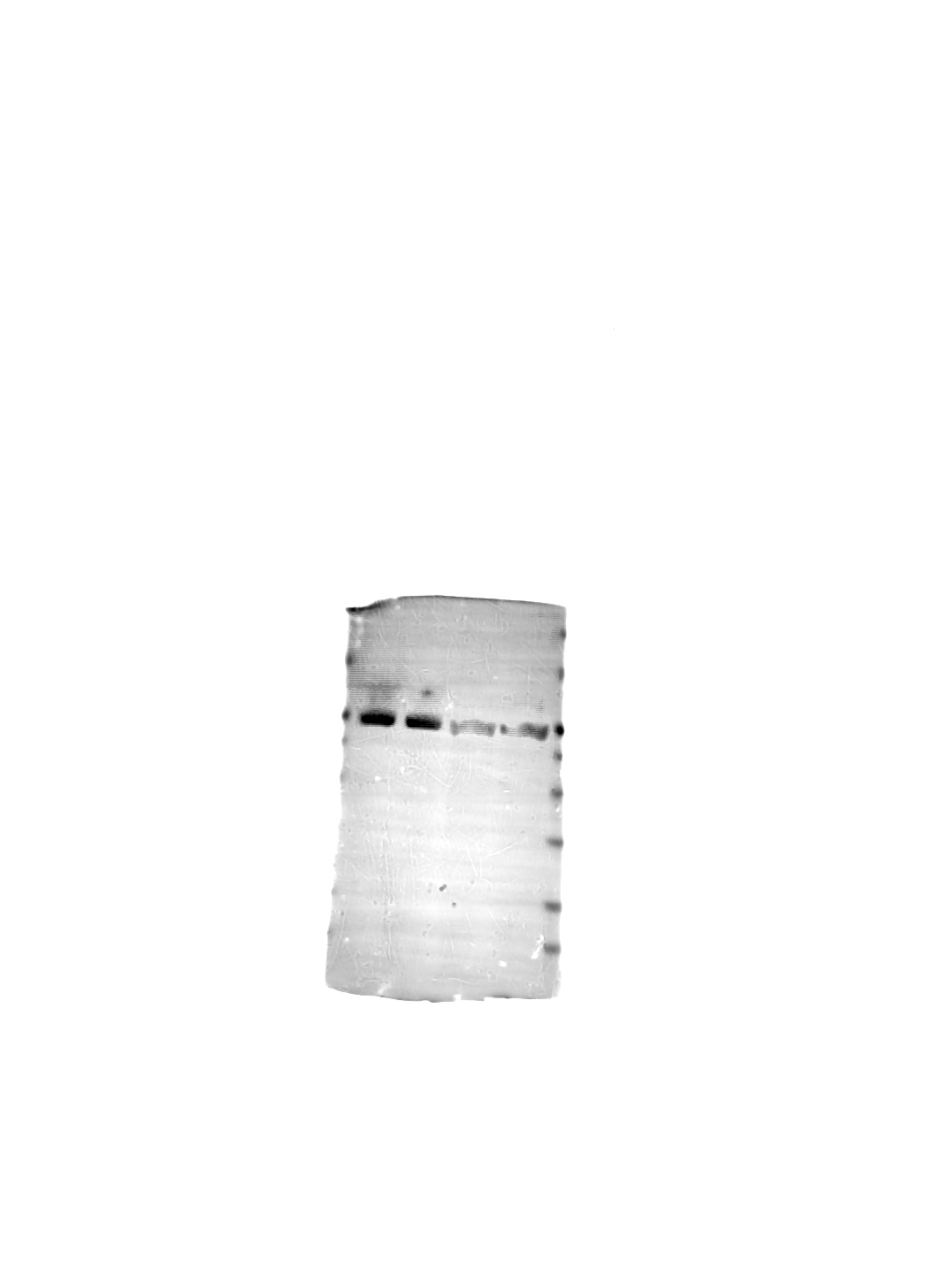

Supplement: Supplementary file 4 [file DataSheet1.zip › Figure1 wb/figure 1 D dlat.tif]

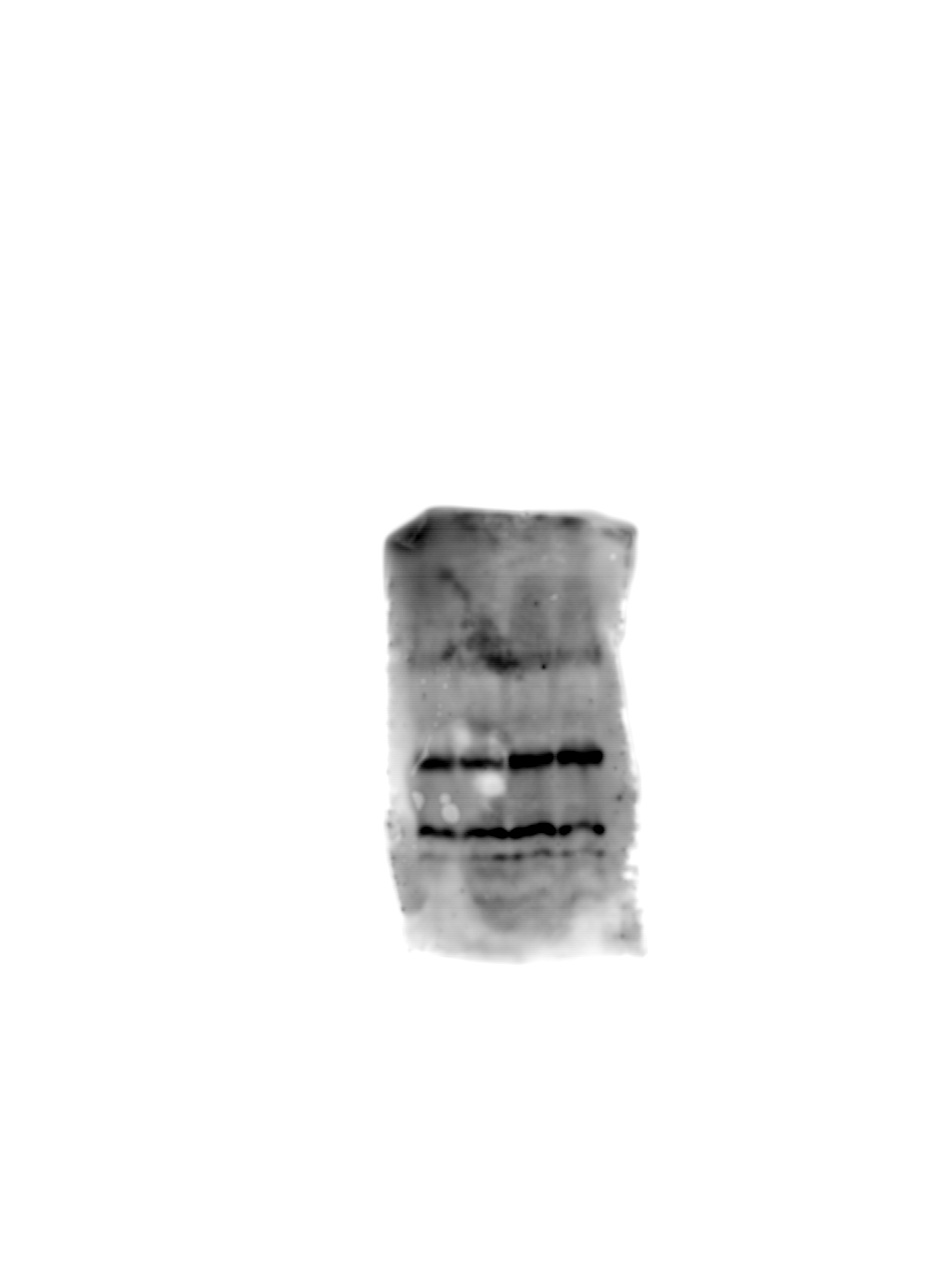

Supplement: Supplementary file 4 [file DataSheet1.zip › Figure1 wb/figure 1 D fdx1.tif]

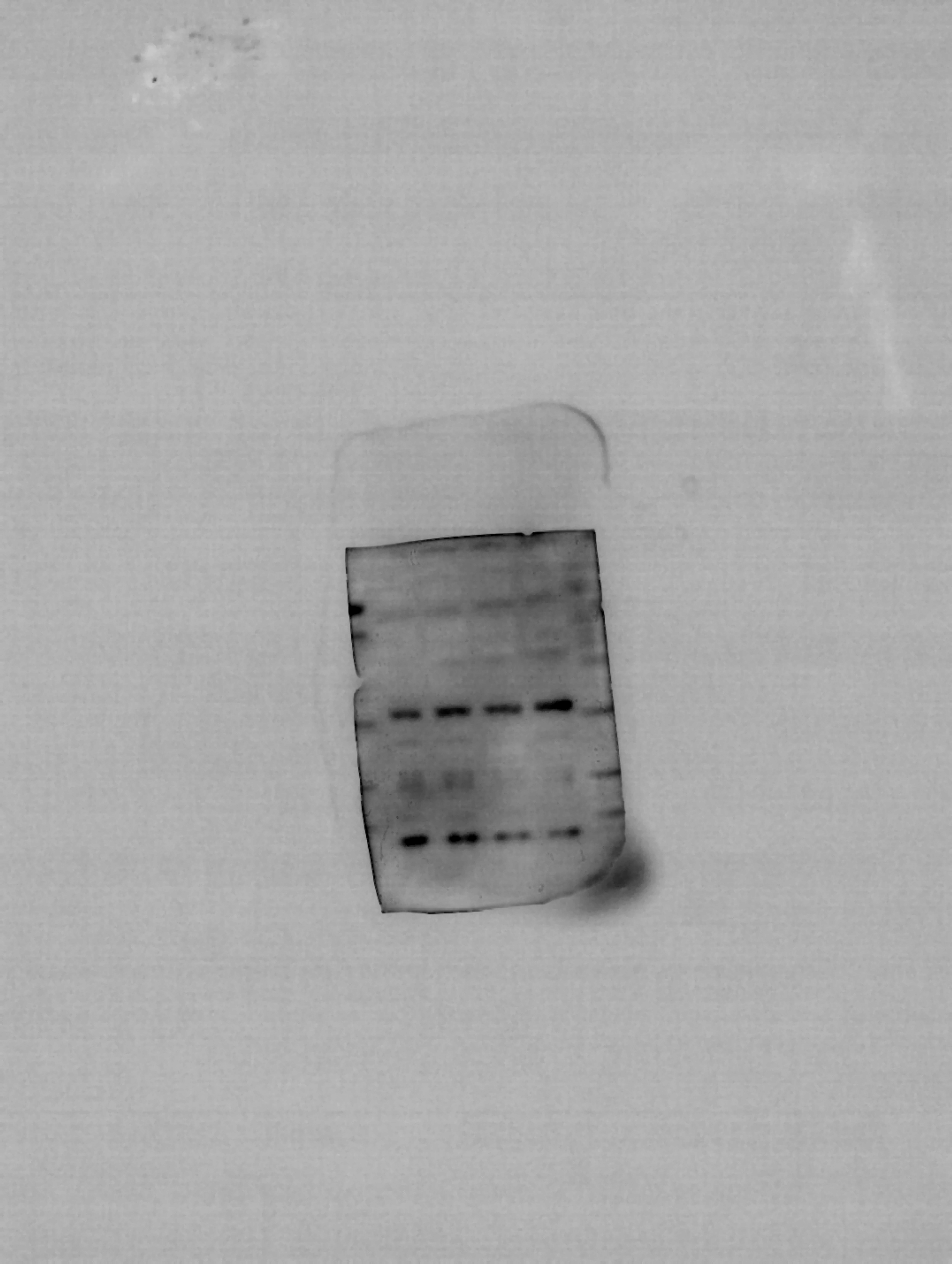

Supplement: Supplementary file 4 [file DataSheet1.zip › Figure1 wb/figure 1 D gapdh 2.tif]

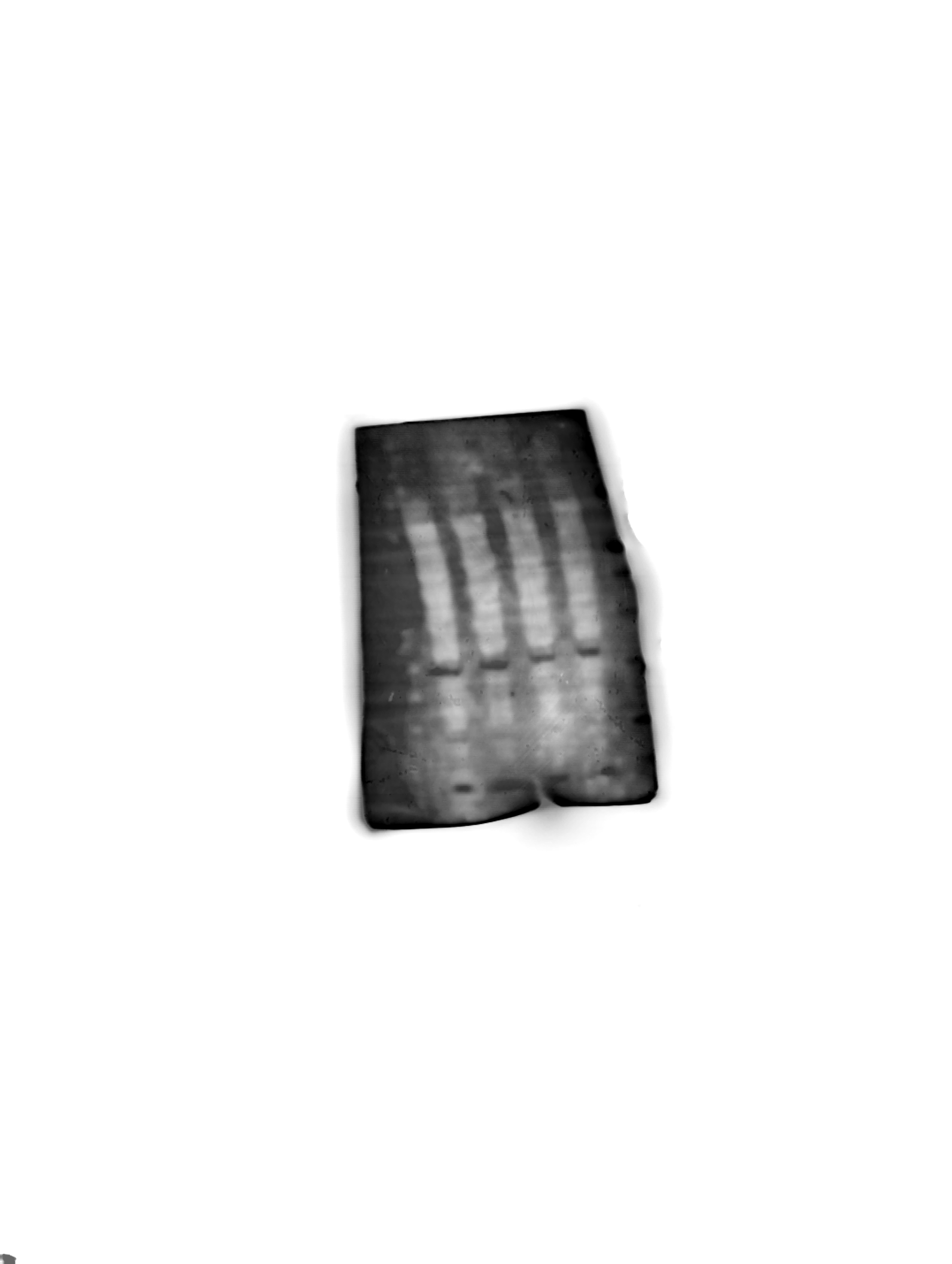

Supplement: Supplementary file 4 [file DataSheet1.zip › Figure1 wb/figure 1 D gapdh 3.tif]

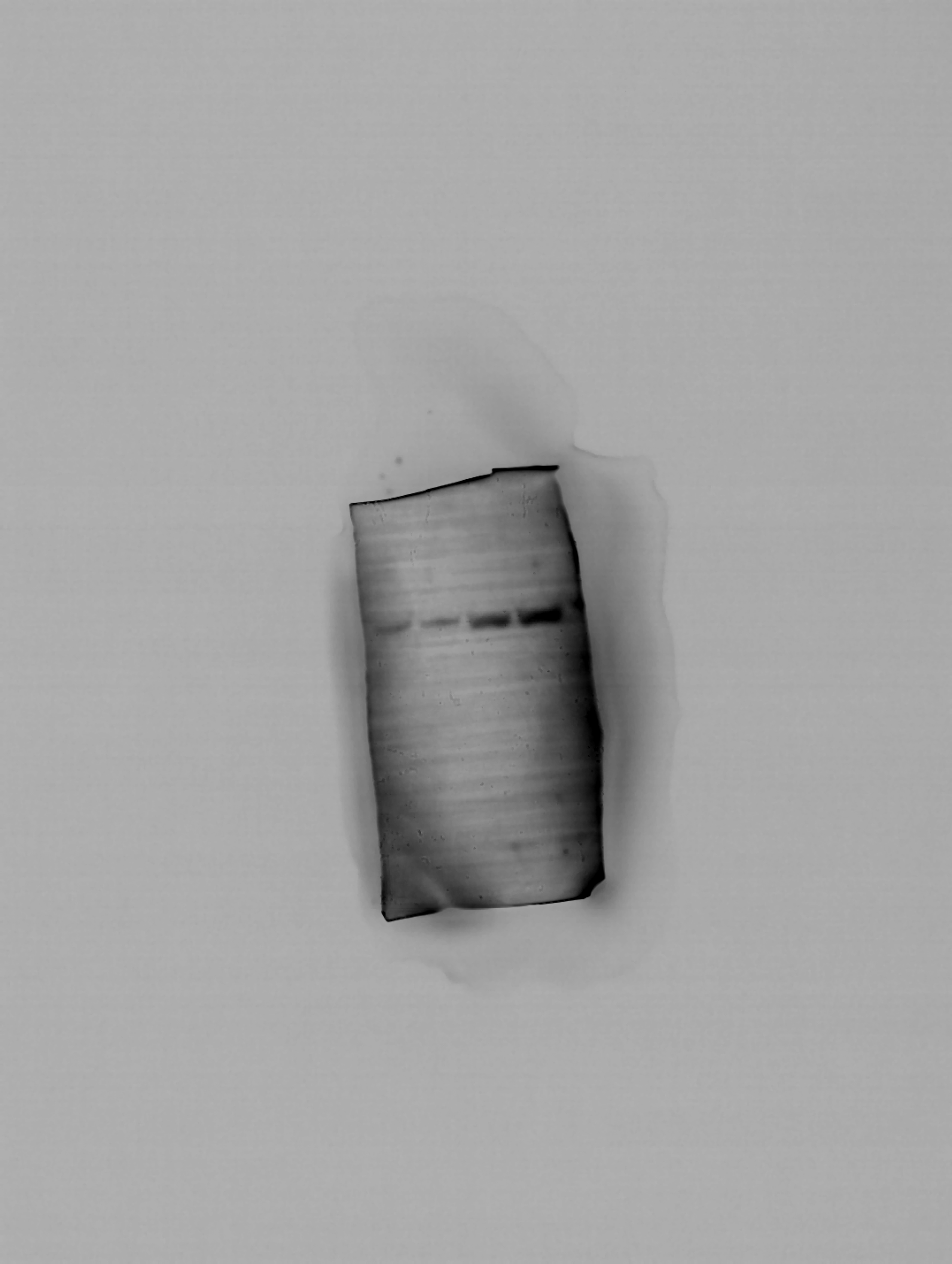

Supplement: Supplementary file 4 [file DataSheet1.zip › Figure1 wb/figure 1 b dlat 3.tif]

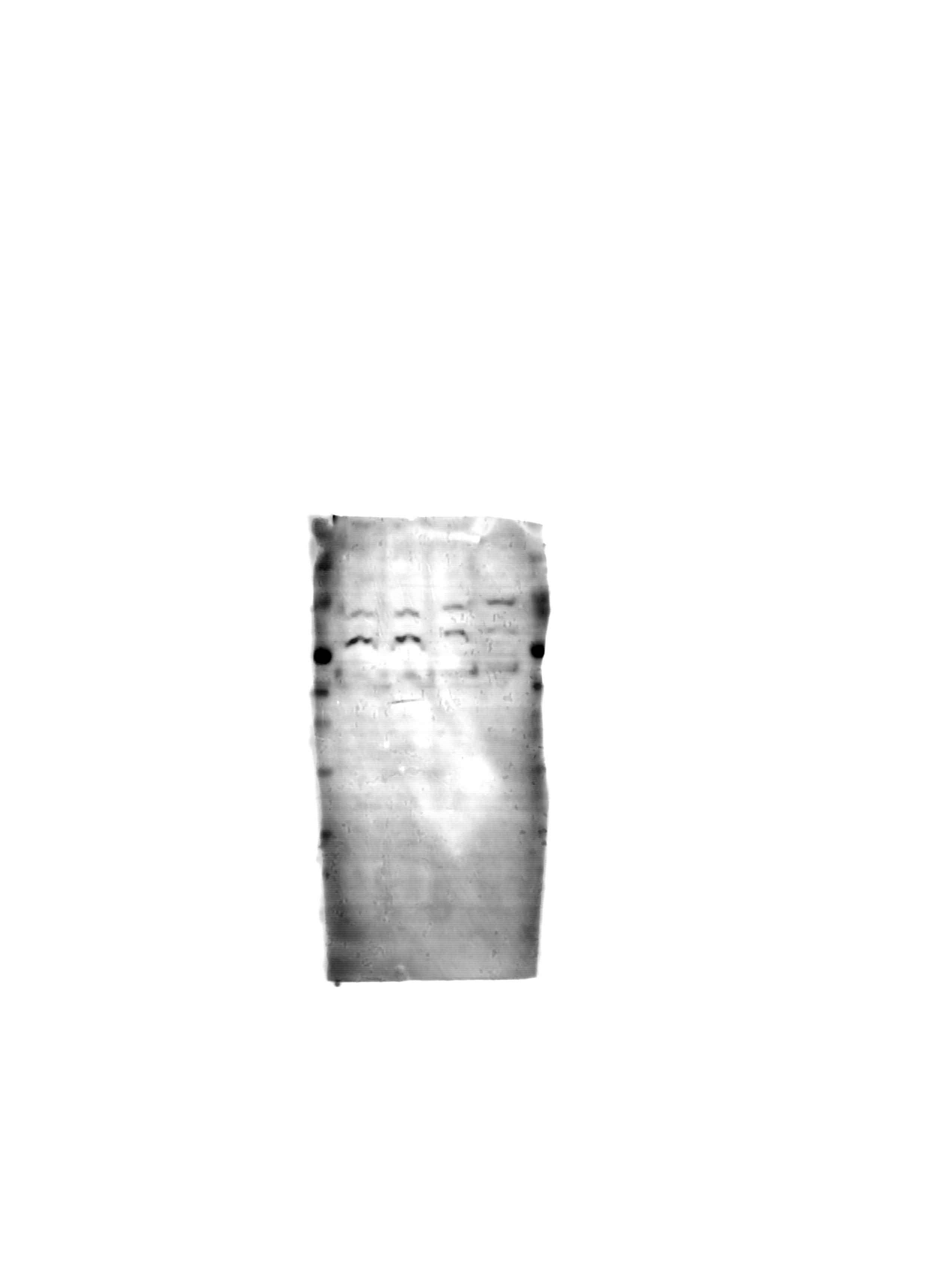

Supplement: Supplementary file 4 [file DataSheet1.zip › Figure1 wb/figure 1 fdx1.tif]

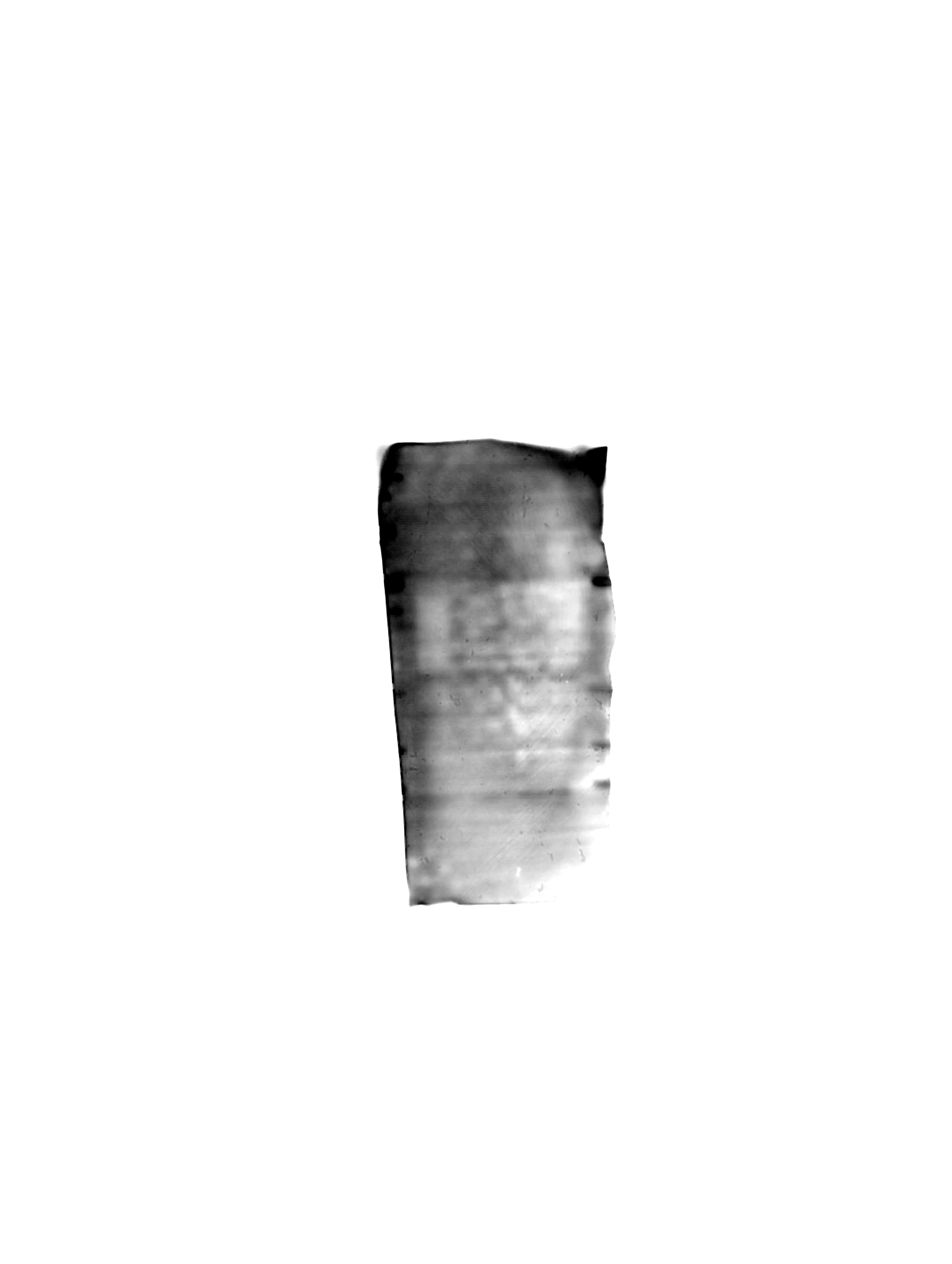

Supplement: Supplementary file 4 [file DataSheet1.zip › Figure1 wb/figure 1C fdx1 2 .tif]

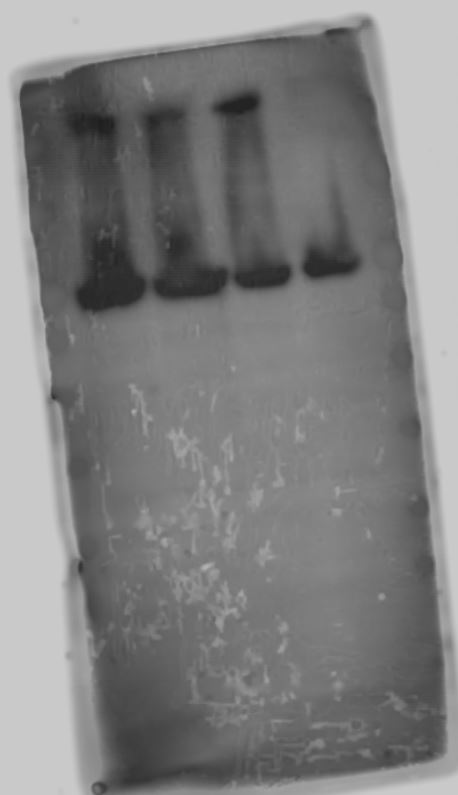

Supplement: Supplementary file 4 [file DataSheet1.zip › Figure1 wb/figure1 B dlat.JPG]

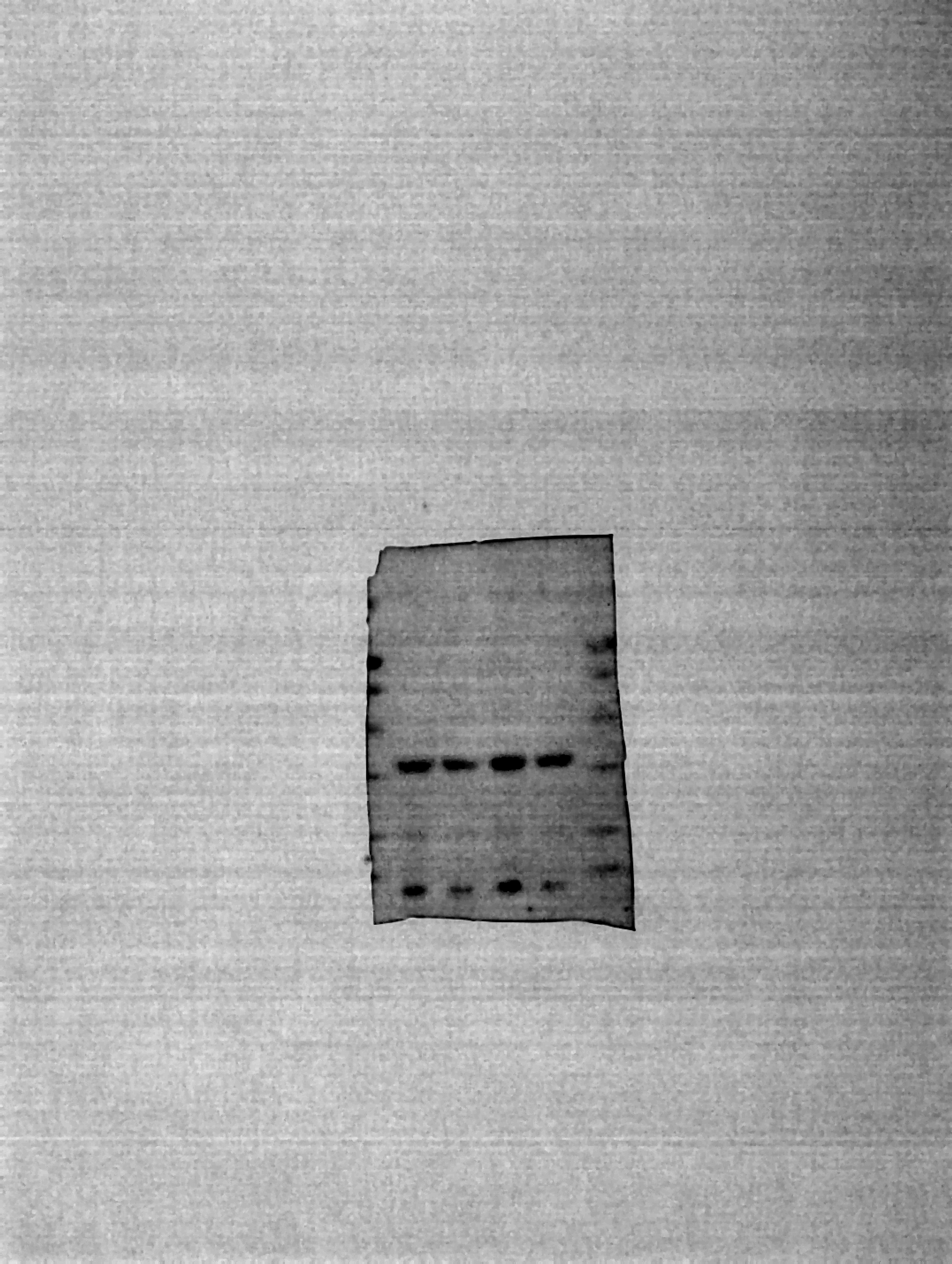

Supplement: Supplementary file 4 [file DataSheet1.zip › Figure1 wb/figure1 B gapdh 2.tif]

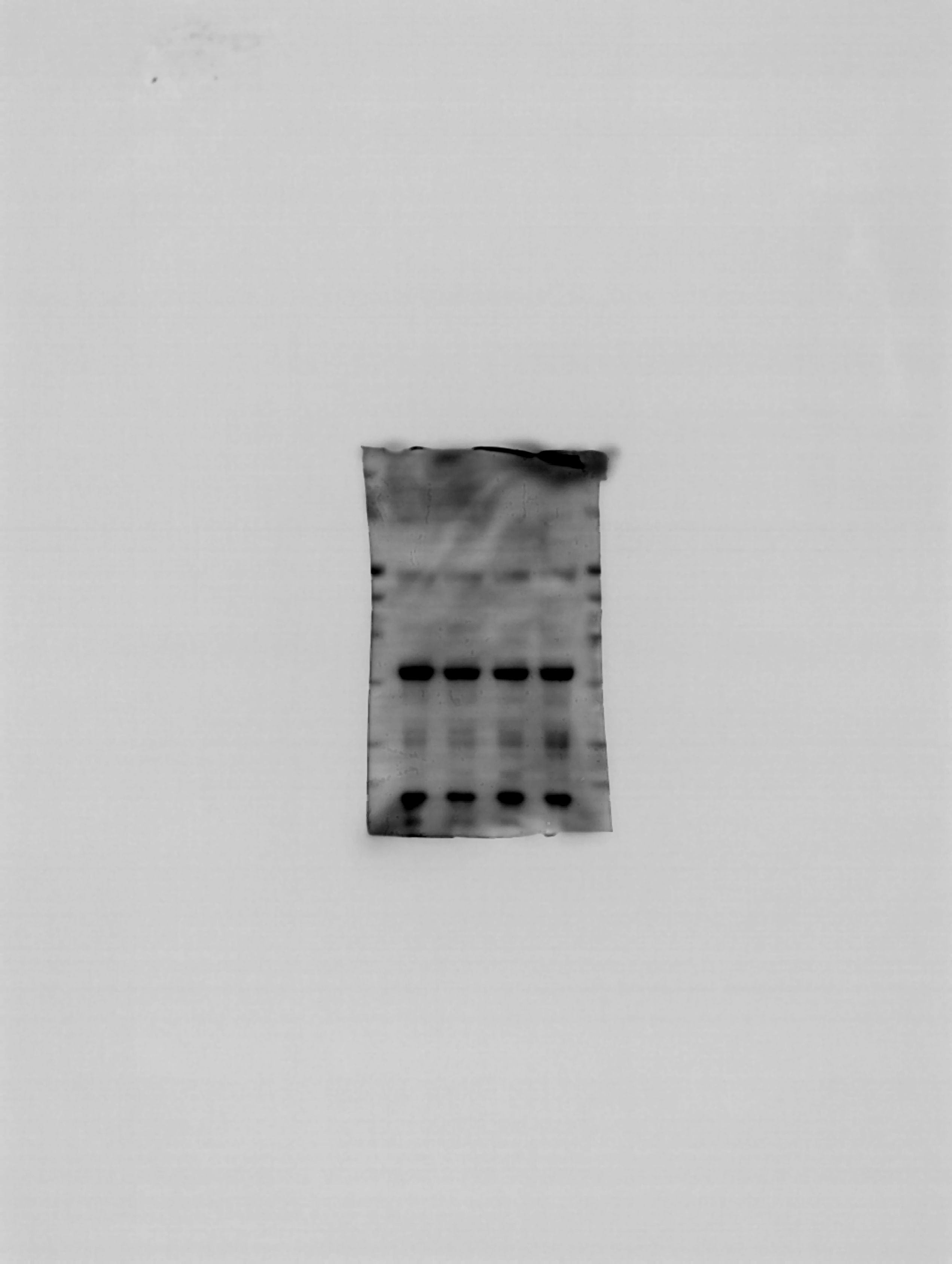

Supplement: Supplementary file 4 [file DataSheet1.zip › Figure1 wb/figure1 B gapdh 3.tif]

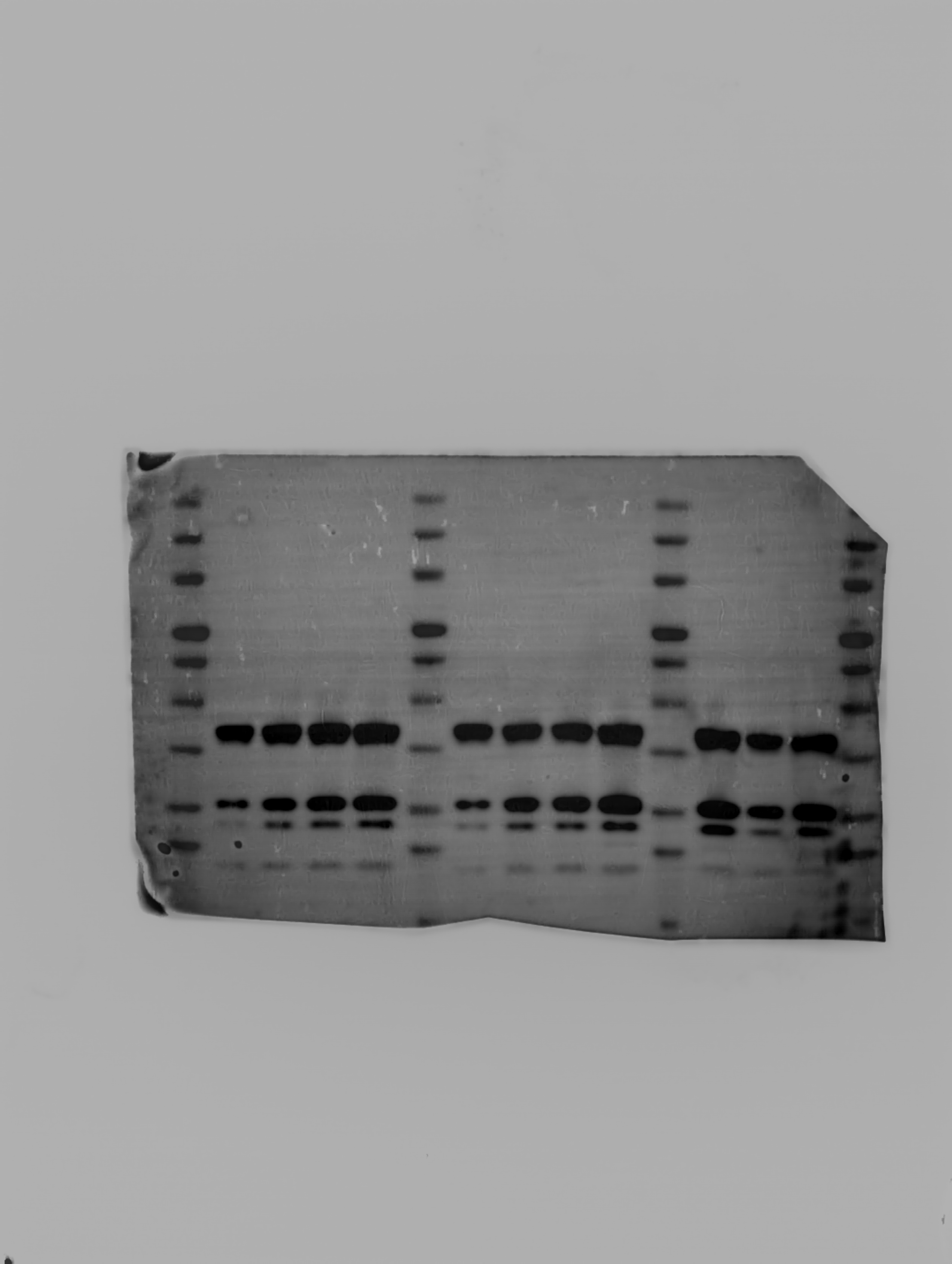

Supplement: Supplementary file 4 [file DataSheet1.zip › Figure1 wb/figure1 B gapdhand figure1 D gapdh .tif]

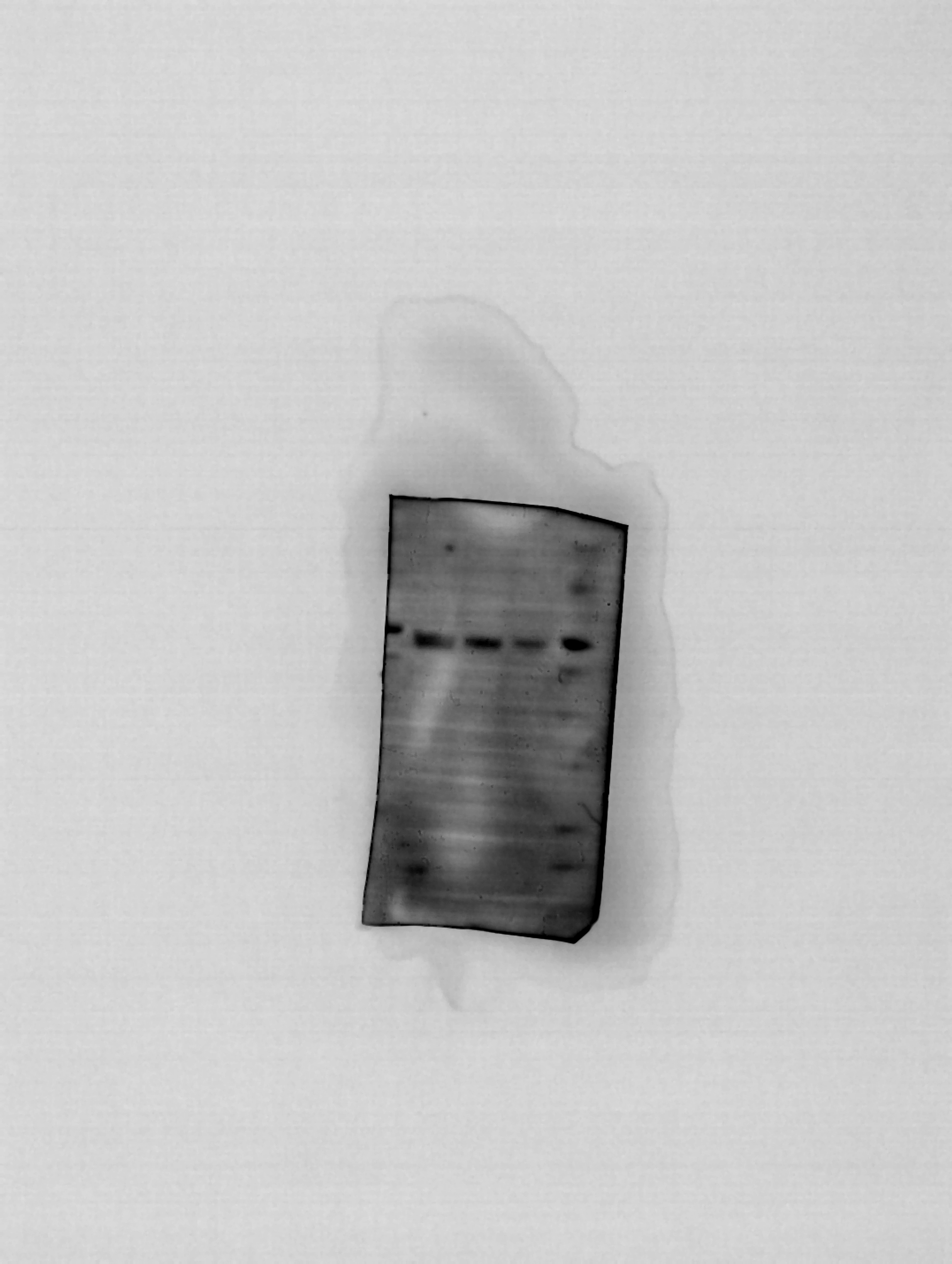

Supplement: Supplementary file 4 [file DataSheet1.zip › Figure1 wb/figure1 C dlat 2.tif]

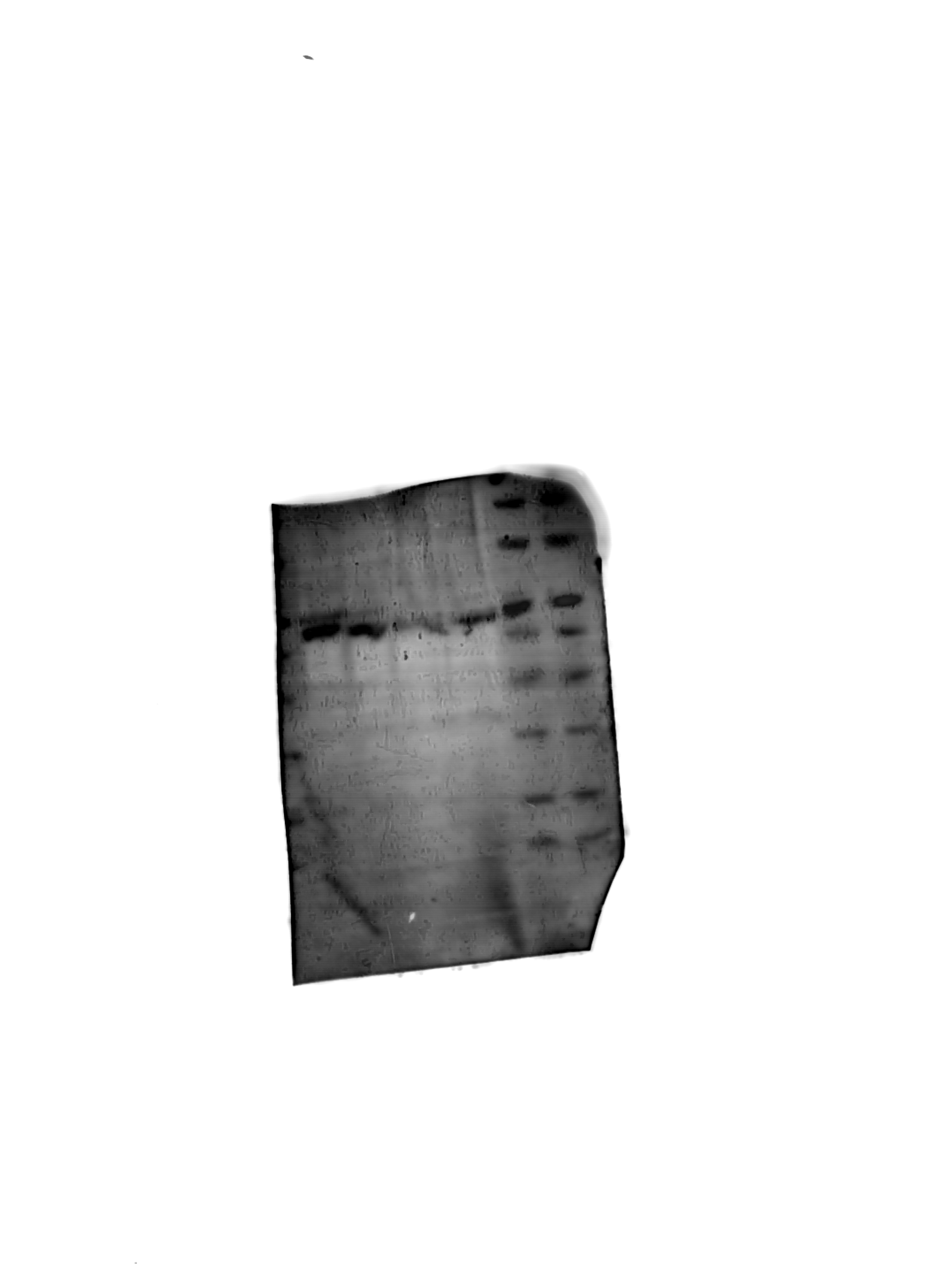

Supplement: Supplementary file 4 [file DataSheet1.zip › Figure1 wb/figure1 D dlat 3.tif]

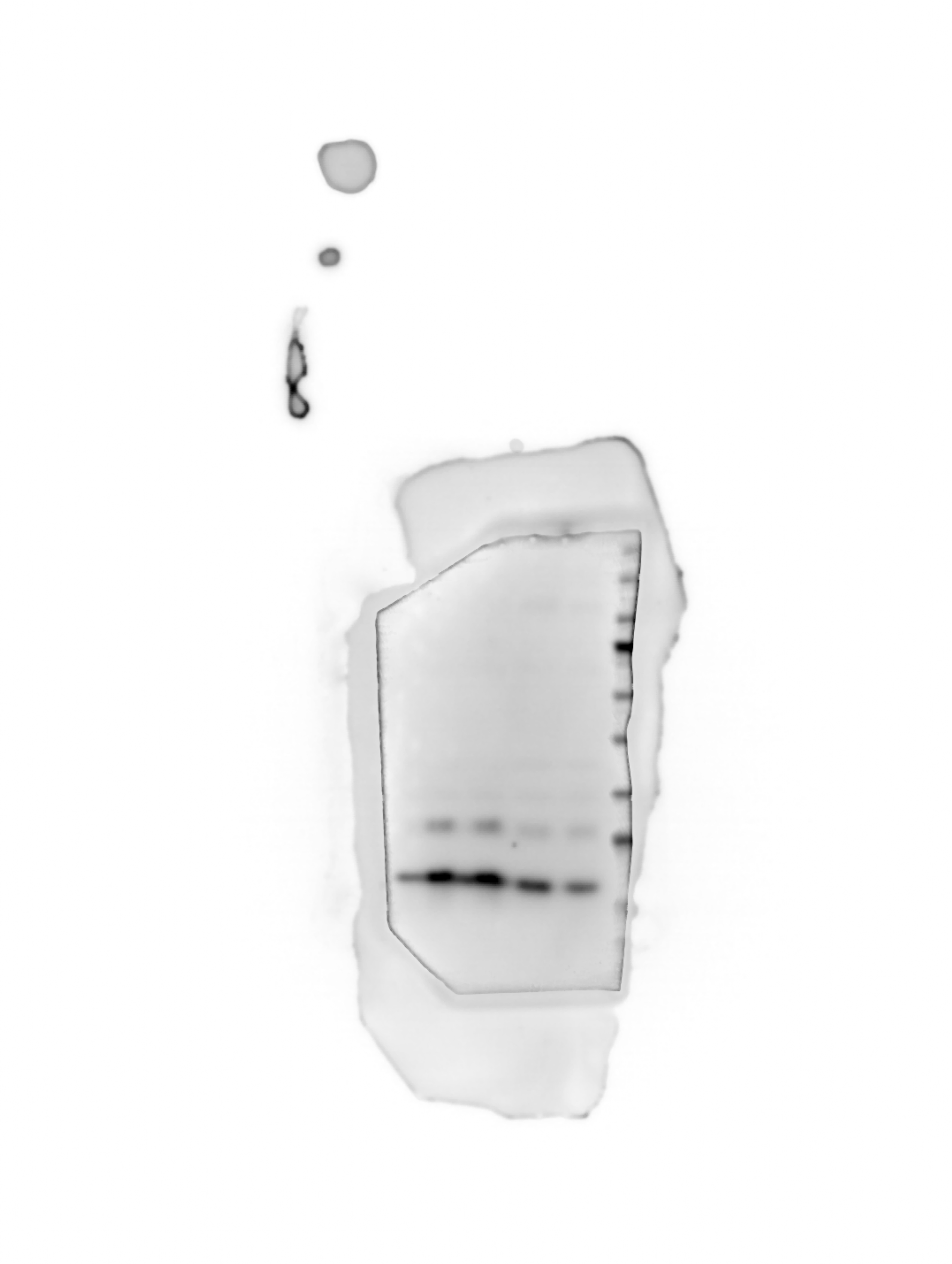

Supplement: Supplementary file 4 [file DataSheet1.zip › Figure1 wb/figure1 D fdx1 2.tif]

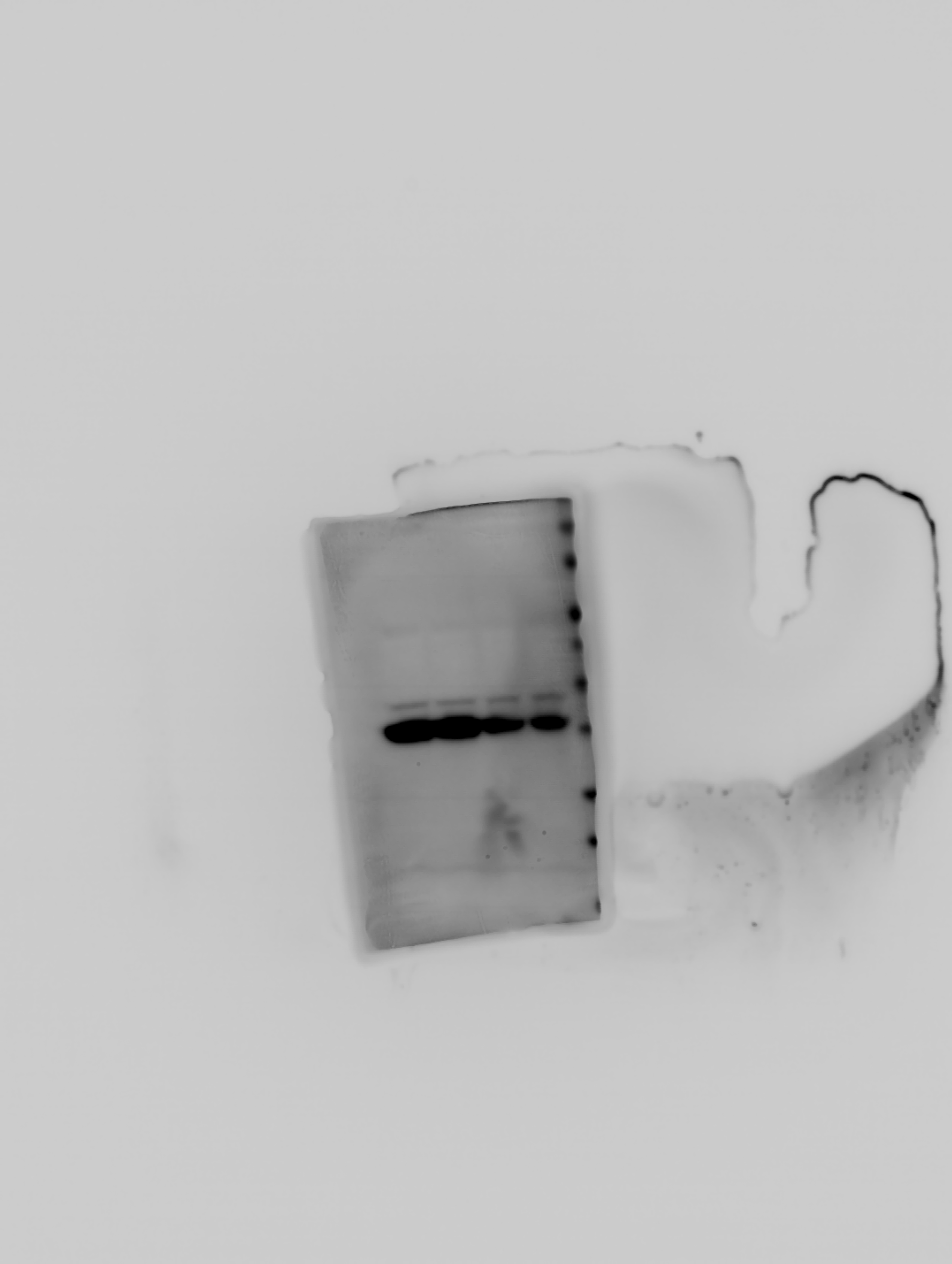

Supplement: Supplementary file 5 [file DataSheet6.zip › WB/GAPDH 2.tif]

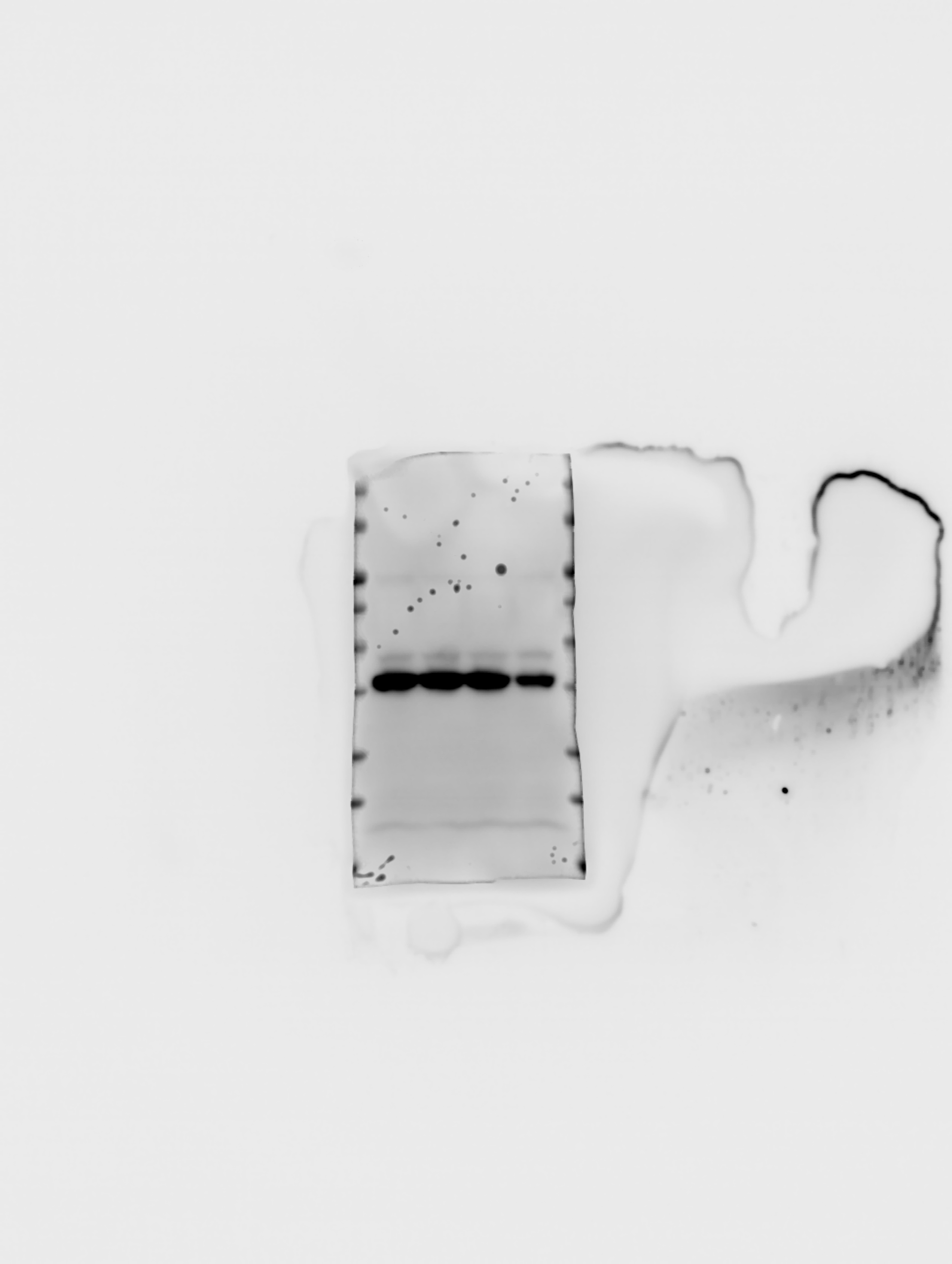

Supplement: Supplementary file 5 [file DataSheet6.zip › WB/GAPDH 3.tif]

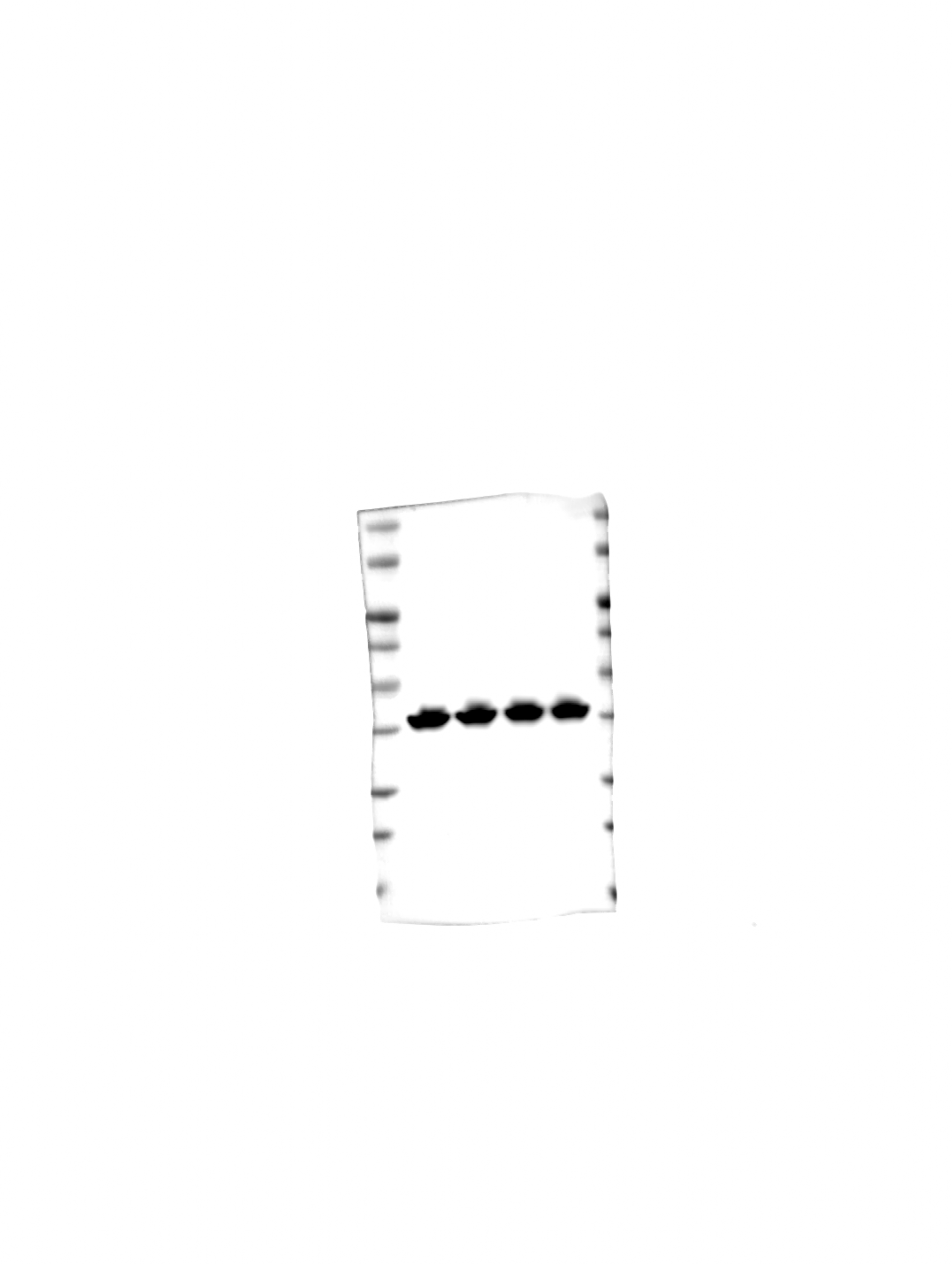

Supplement: Supplementary file 5 [file DataSheet6.zip › WB/GAPDH XH 1.tif]

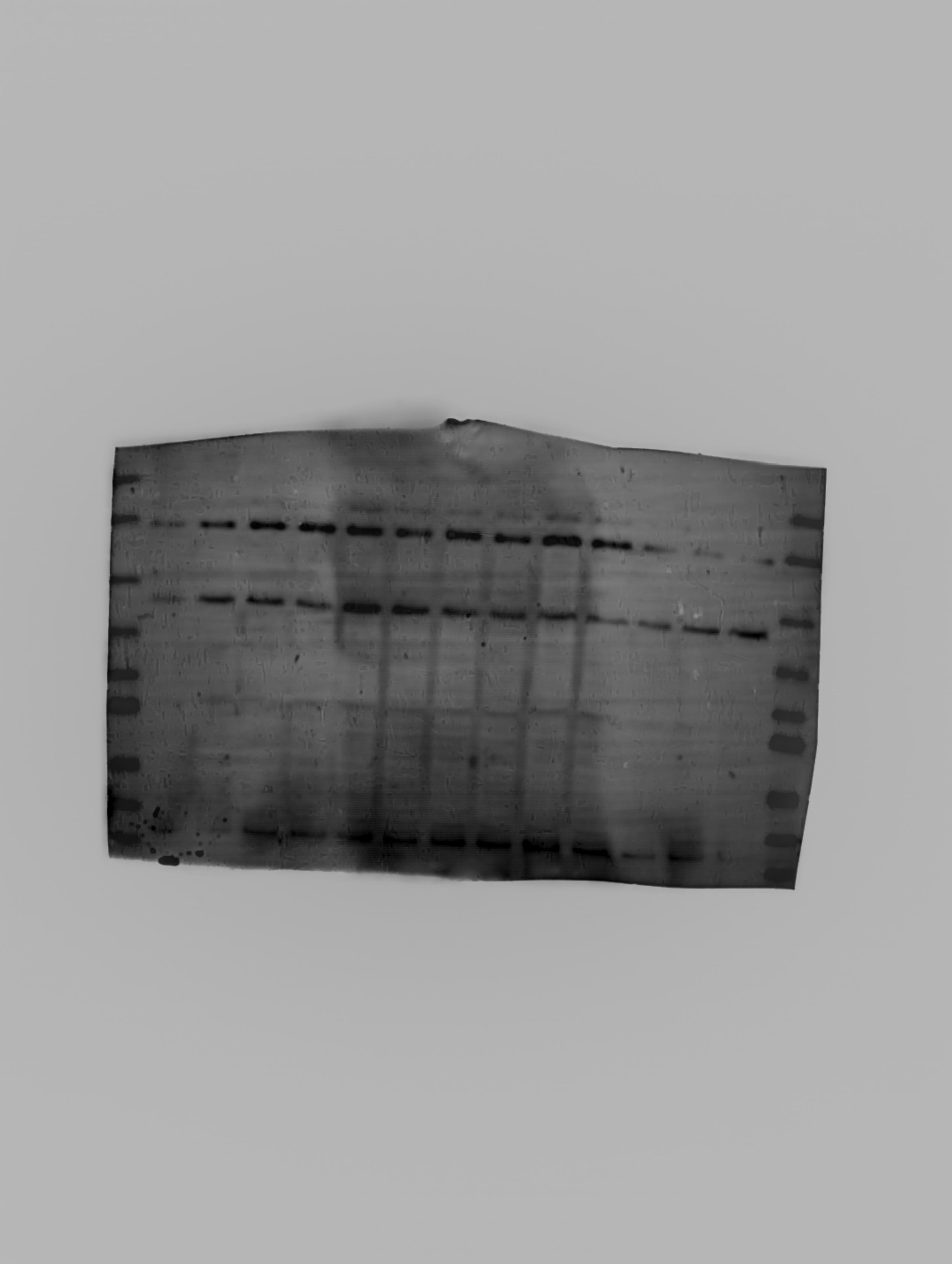

Supplement: Supplementary file 6 [file DataSheet2.zip › figure2 wb/figure 2 C 2.tif]

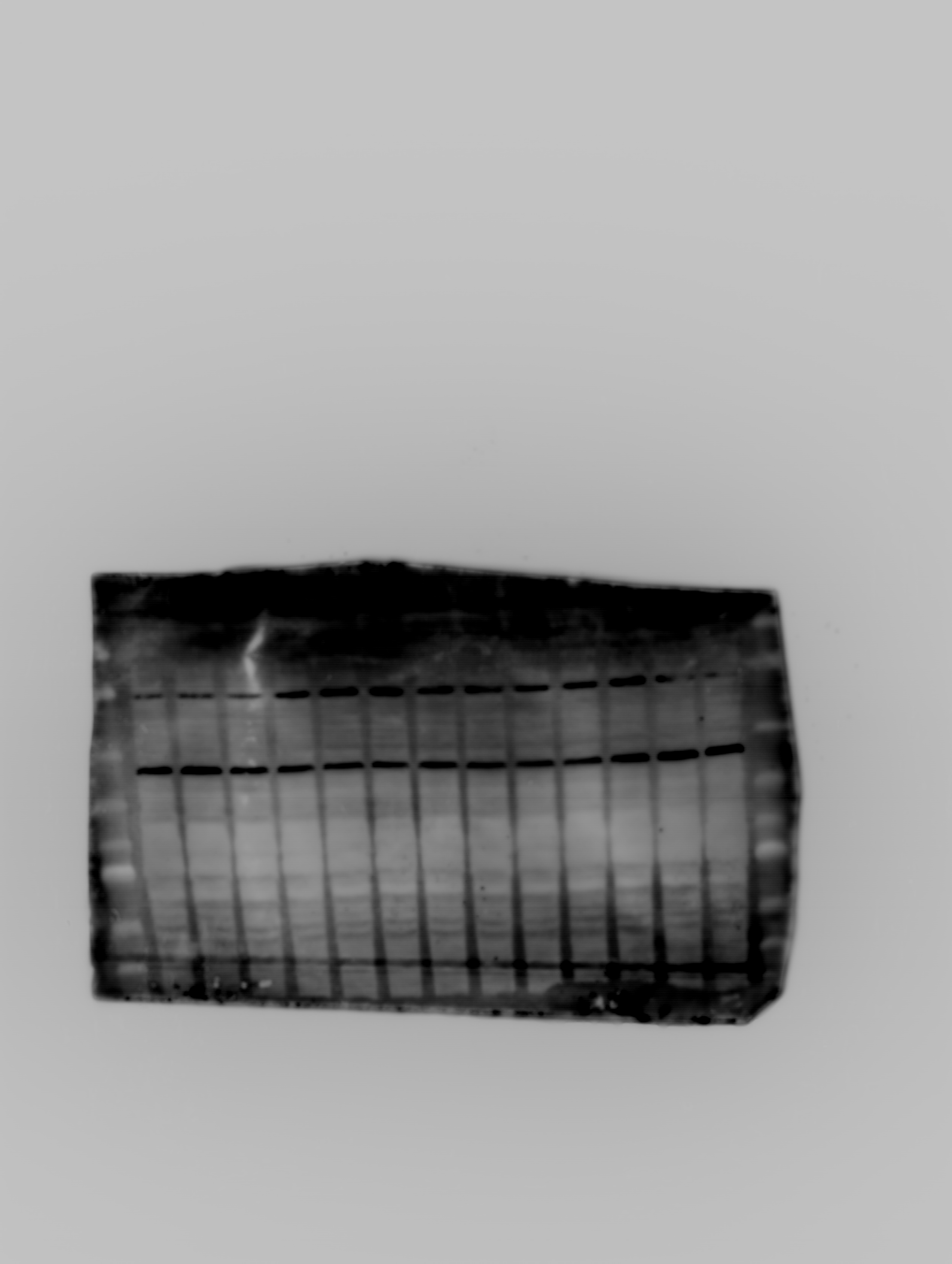

Supplement: Supplementary file 6 [file DataSheet2.zip › figure2 wb/figure 2 C 3.tif]

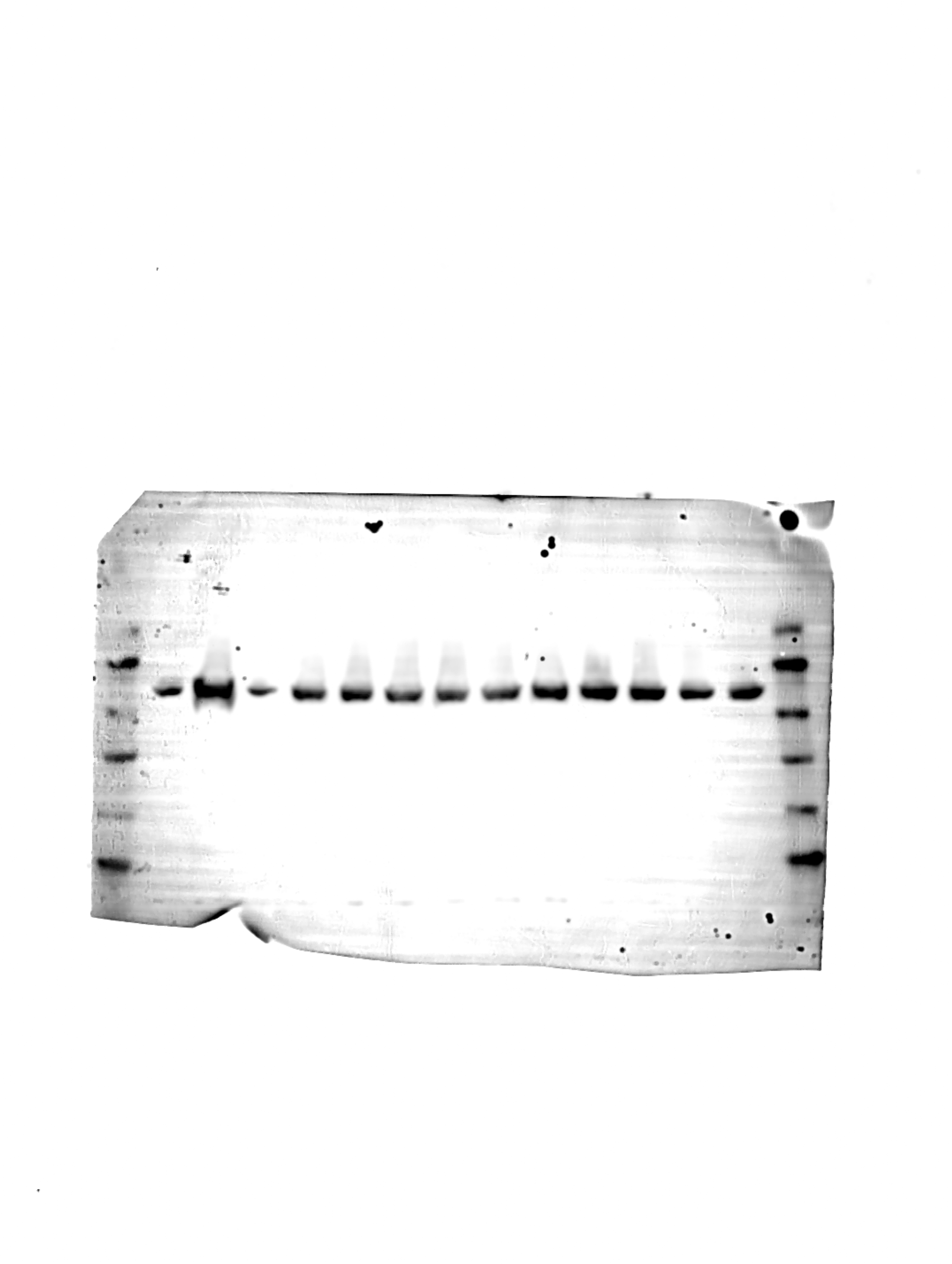

Supplement: Supplementary file 6 [file DataSheet2.zip › figure2 wb/figure 2 C dlat3 .tif]

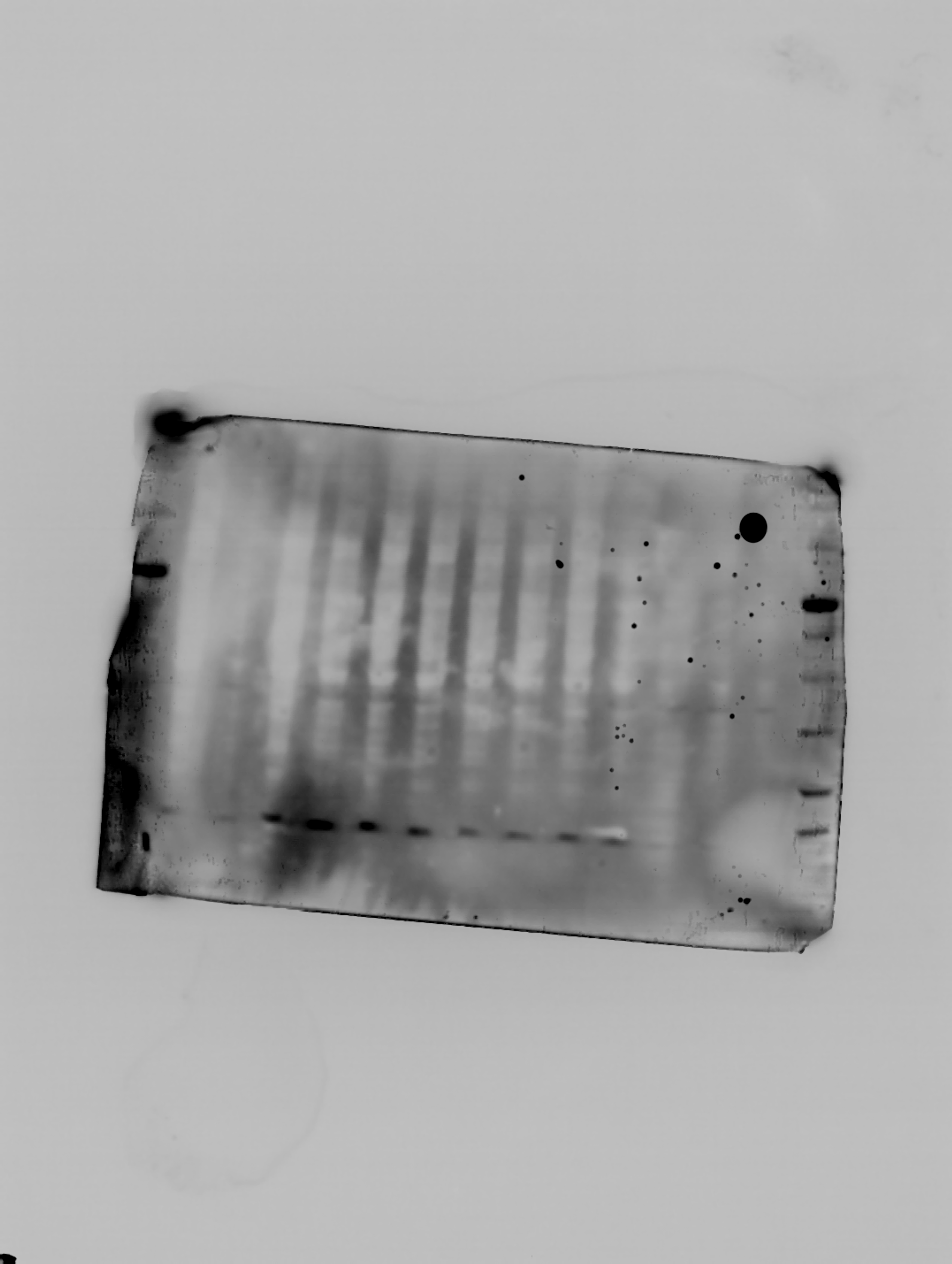

Supplement: Supplementary file 6 [file DataSheet2.zip › figure2 wb/figure 2 C fdx1 2.tif]

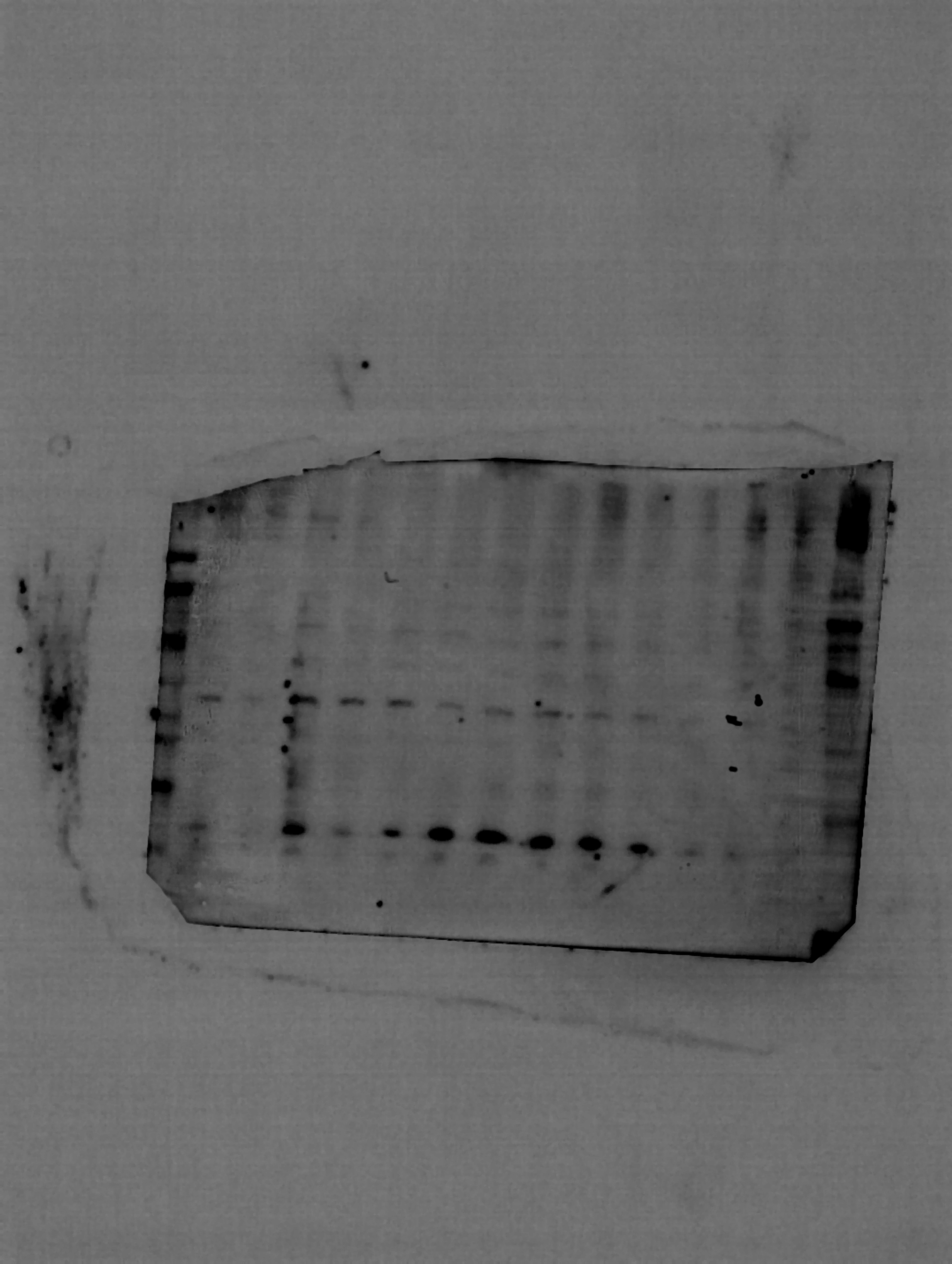

Supplement: Supplementary file 6 [file DataSheet2.zip › figure2 wb/figure 2 C fdx1 3.tif]

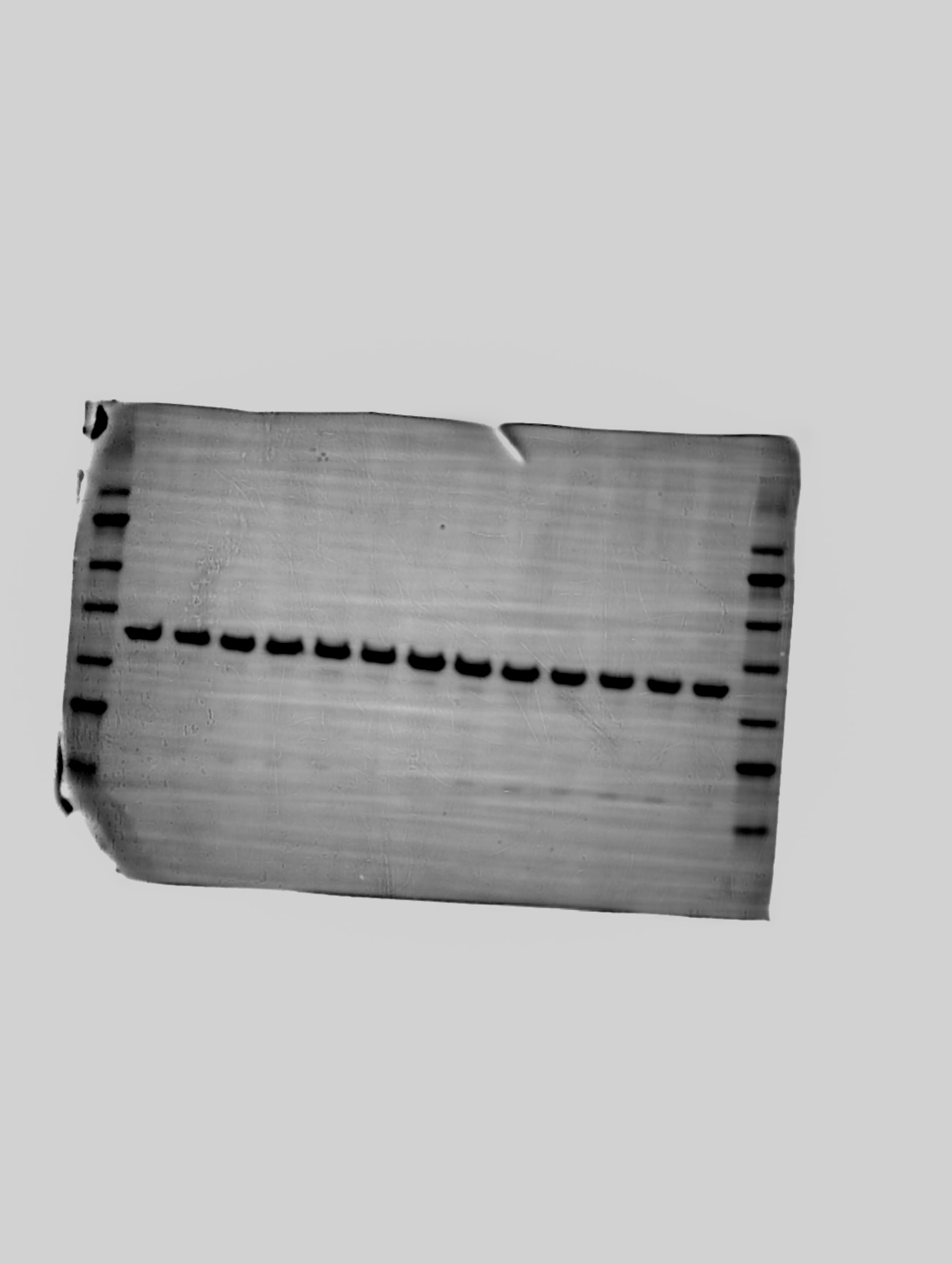

Supplement: Supplementary file 6 [file DataSheet2.zip › figure2 wb/figure 2 C gapdh 2.tif]

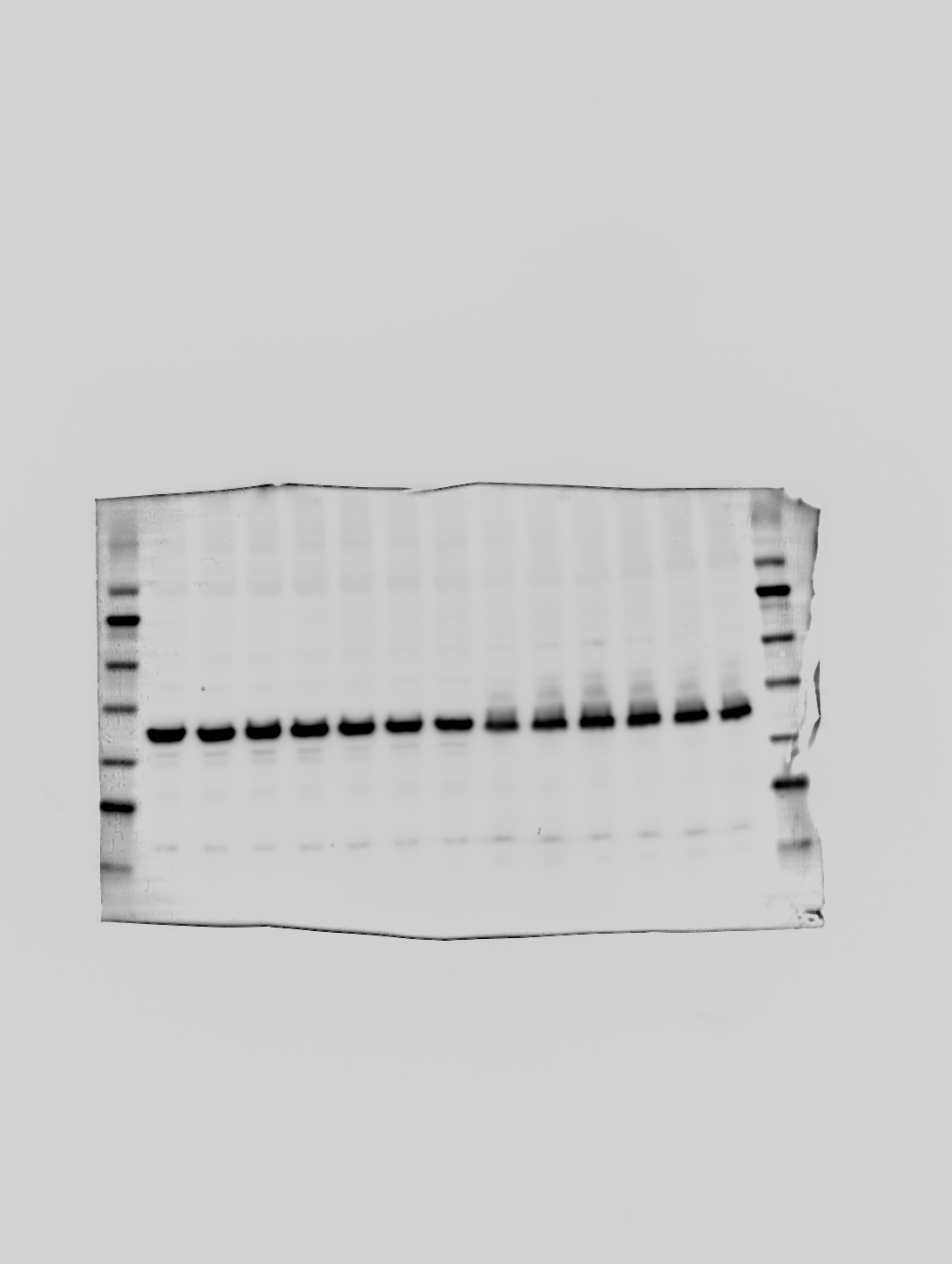

Supplement: Supplementary file 6 [file DataSheet2.zip › figure2 wb/figure 2 C gapdh 3.tif]

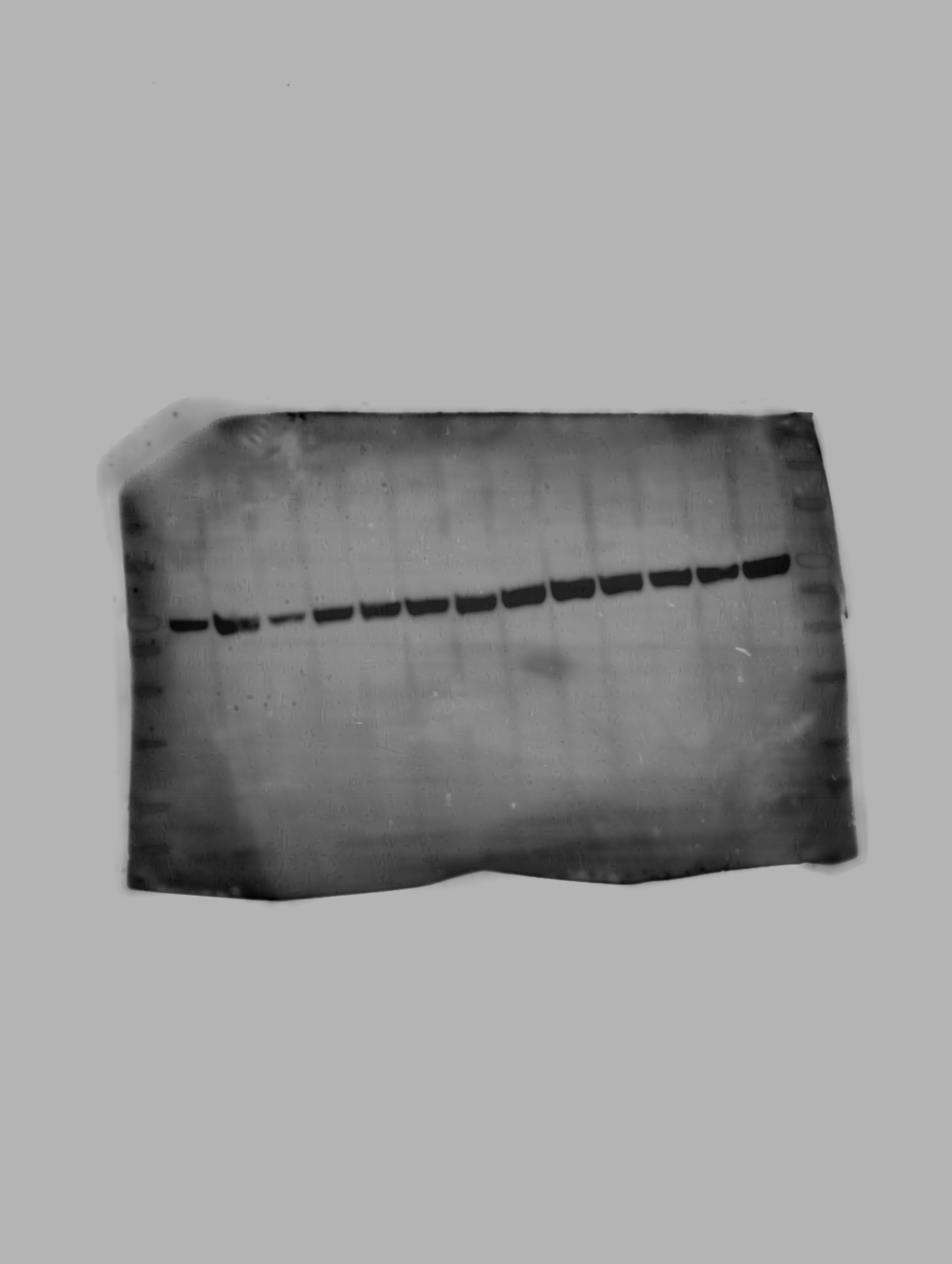

Supplement: Supplementary file 6 [file DataSheet2.zip › figure2 wb/figure 2 C1.tif]

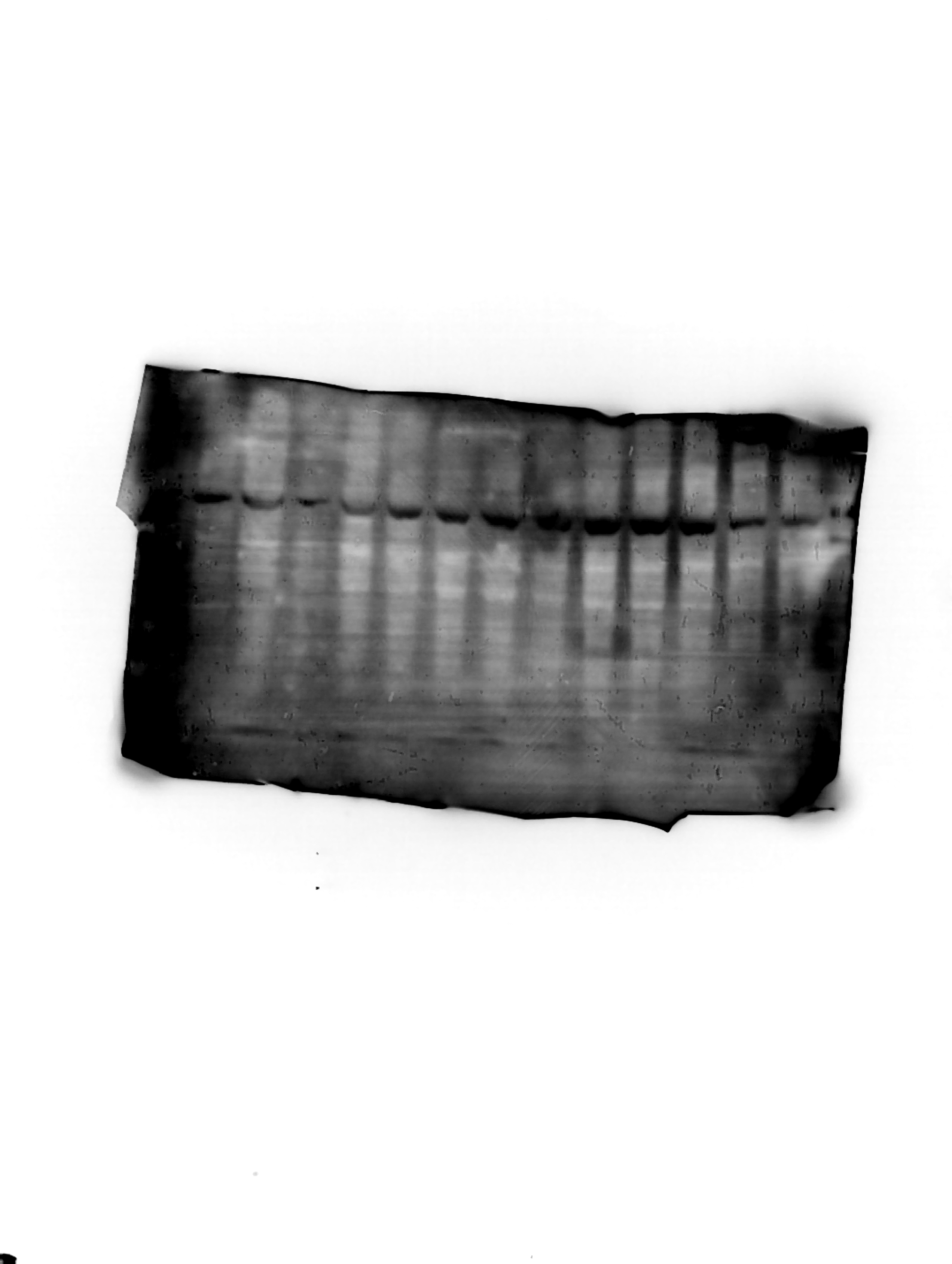

Supplement: Supplementary file 6 [file DataSheet2.zip › figure2 wb/figure2 C dlat 2.tif]

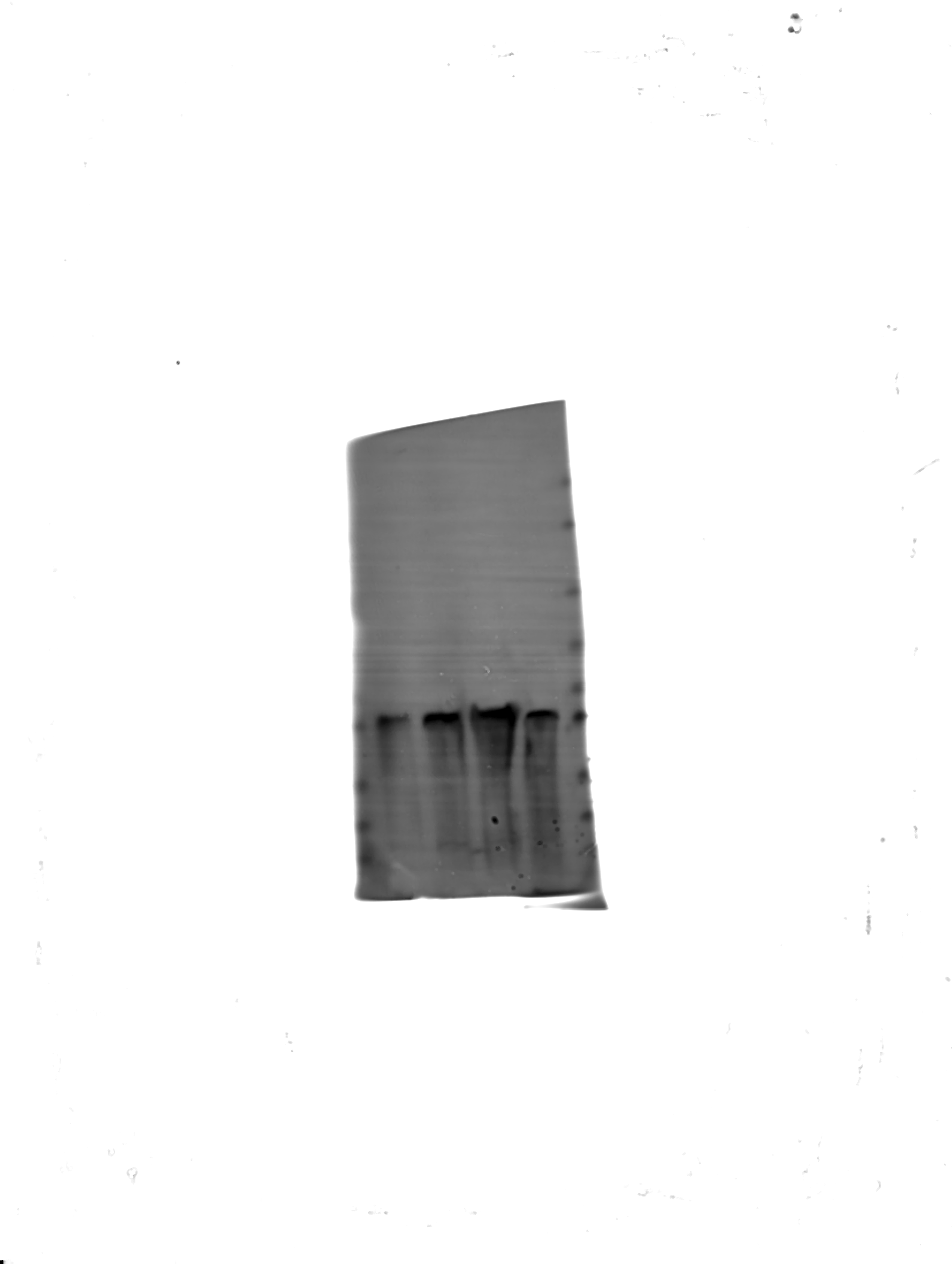

Supplement: Supplementary file 7 [file DataSheet5.zip › figure6 wb/figure 6 A 1.tif]

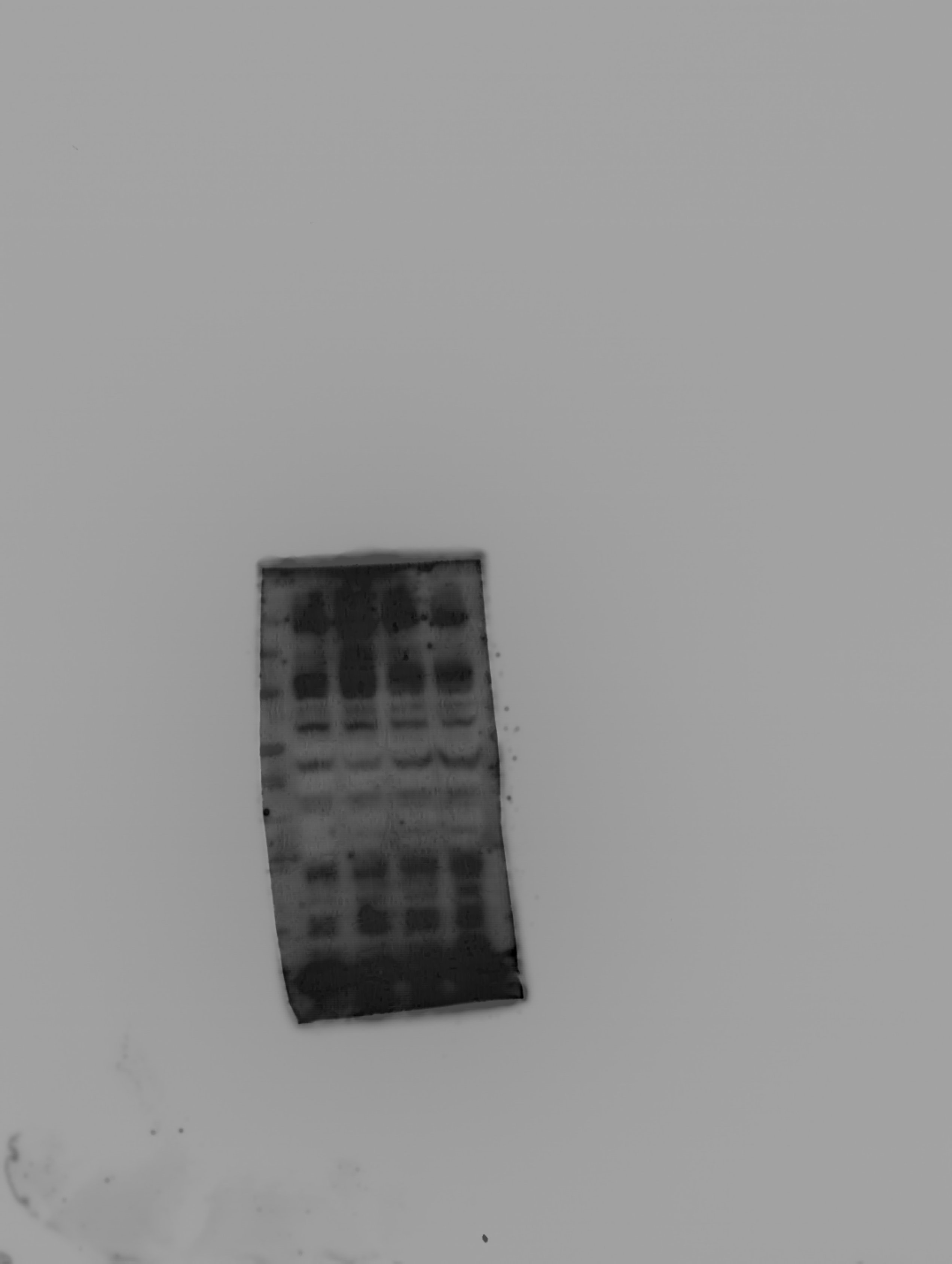

Supplement: Supplementary file 7 [file DataSheet5.zip › figure6 wb/figure 6 A 2.tif]

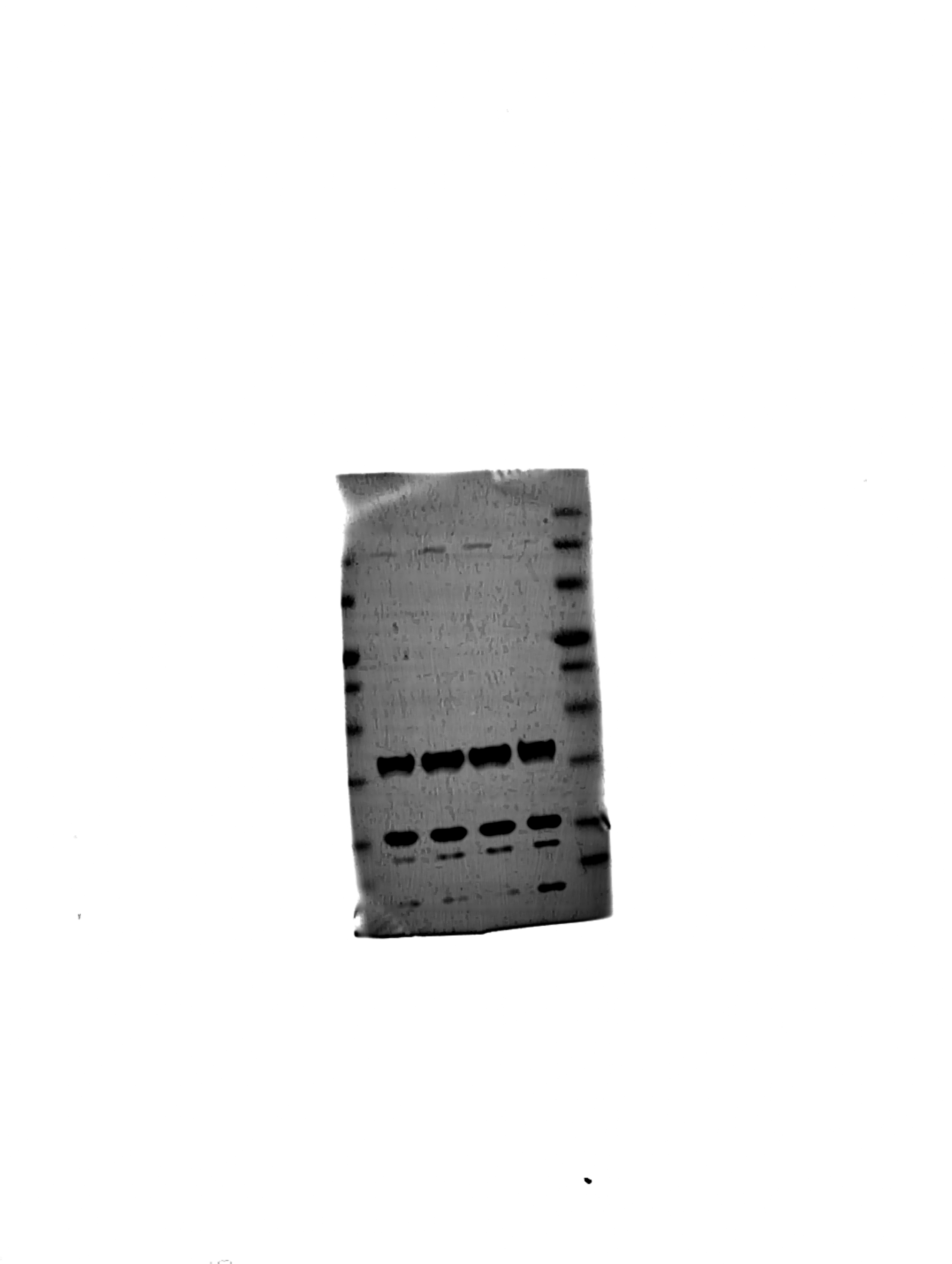

Supplement: Supplementary file 7 [file DataSheet5.zip › figure6 wb/figure 6 A 3.tif]

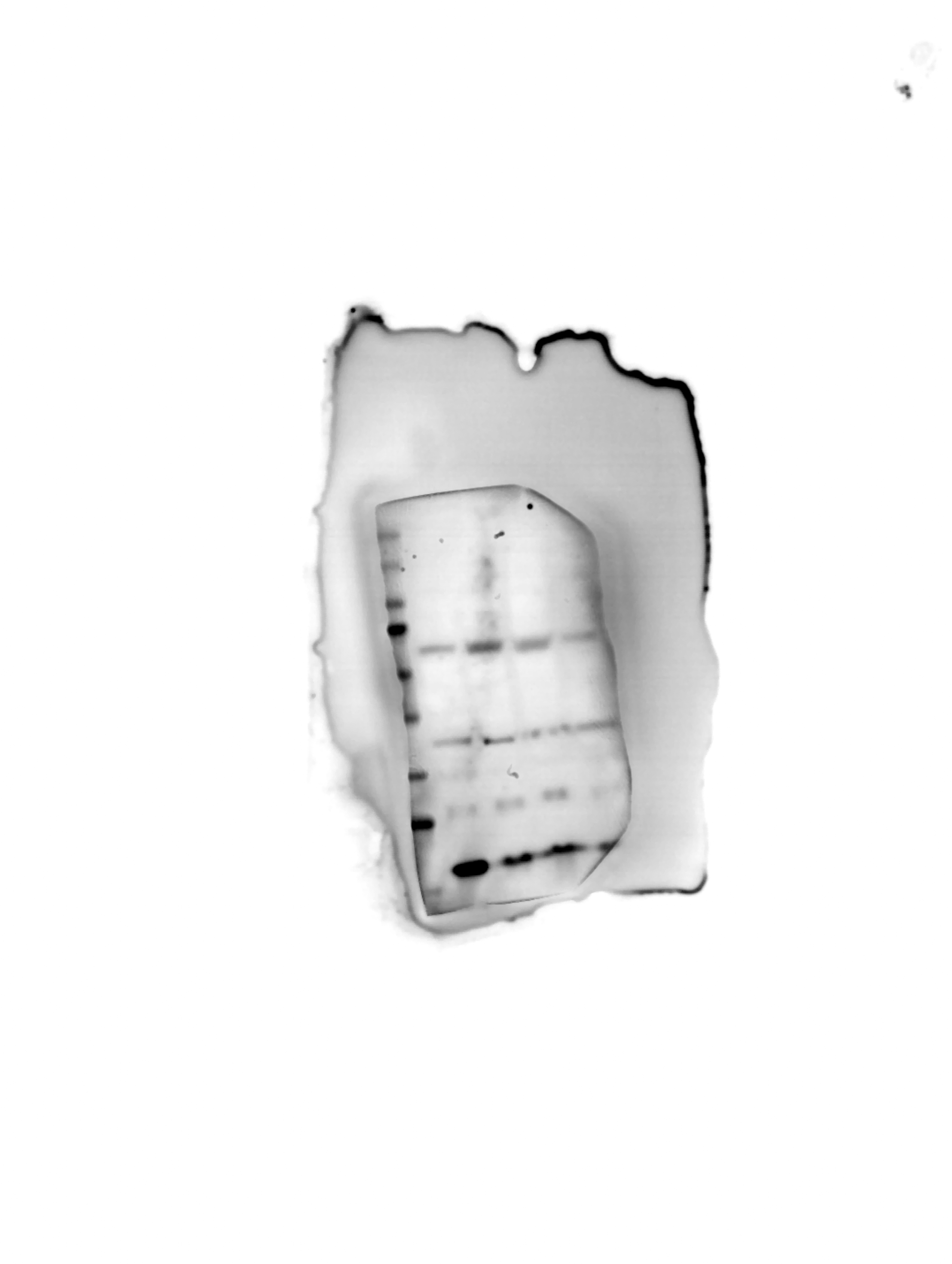

Supplement: Supplementary file 7 [file DataSheet5.zip › figure6 wb/figure 6 A dlat 3.tif]

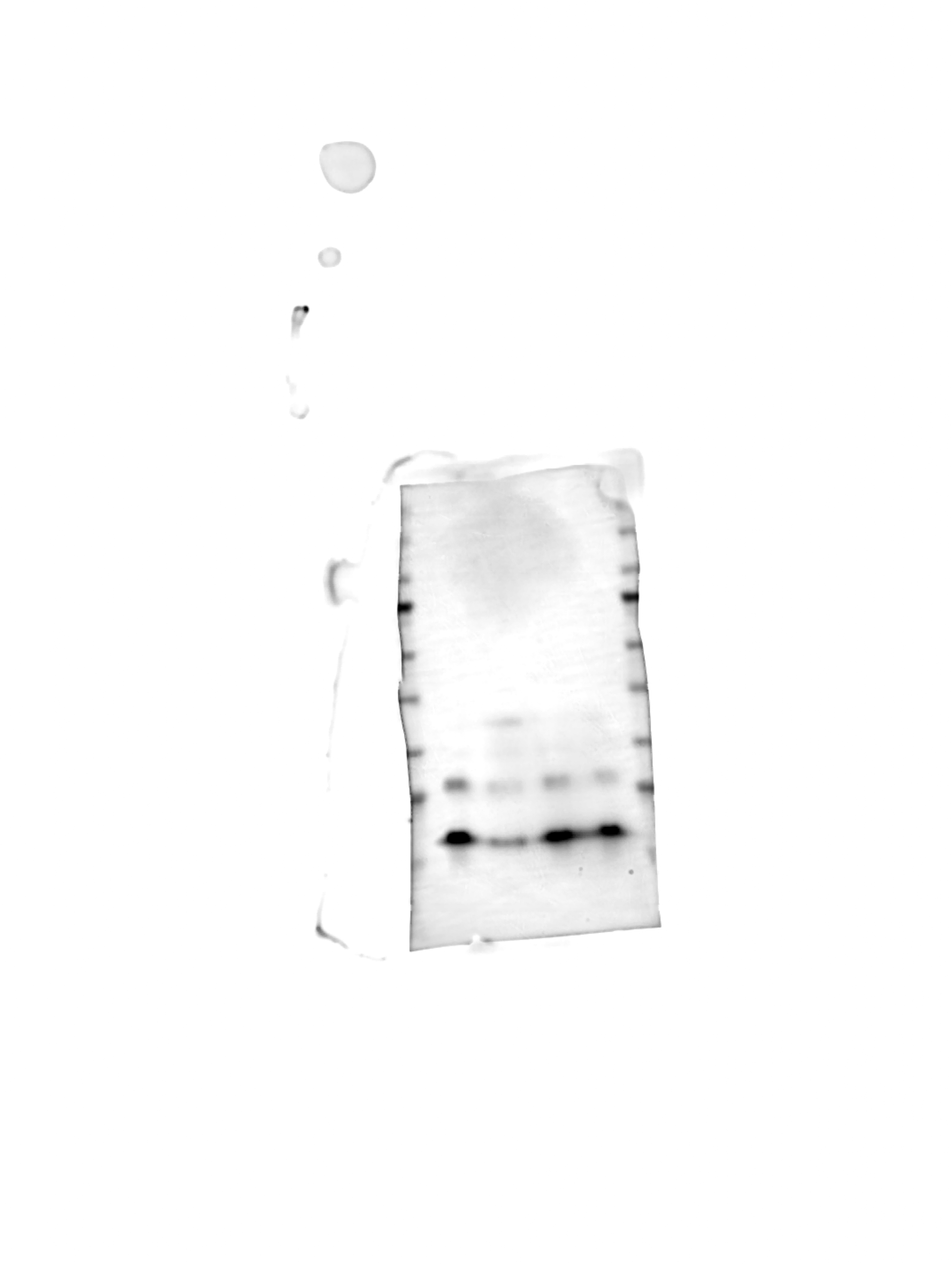

Supplement: Supplementary file 7 [file DataSheet5.zip › figure6 wb/figure 6 A fdx1 2.tif]

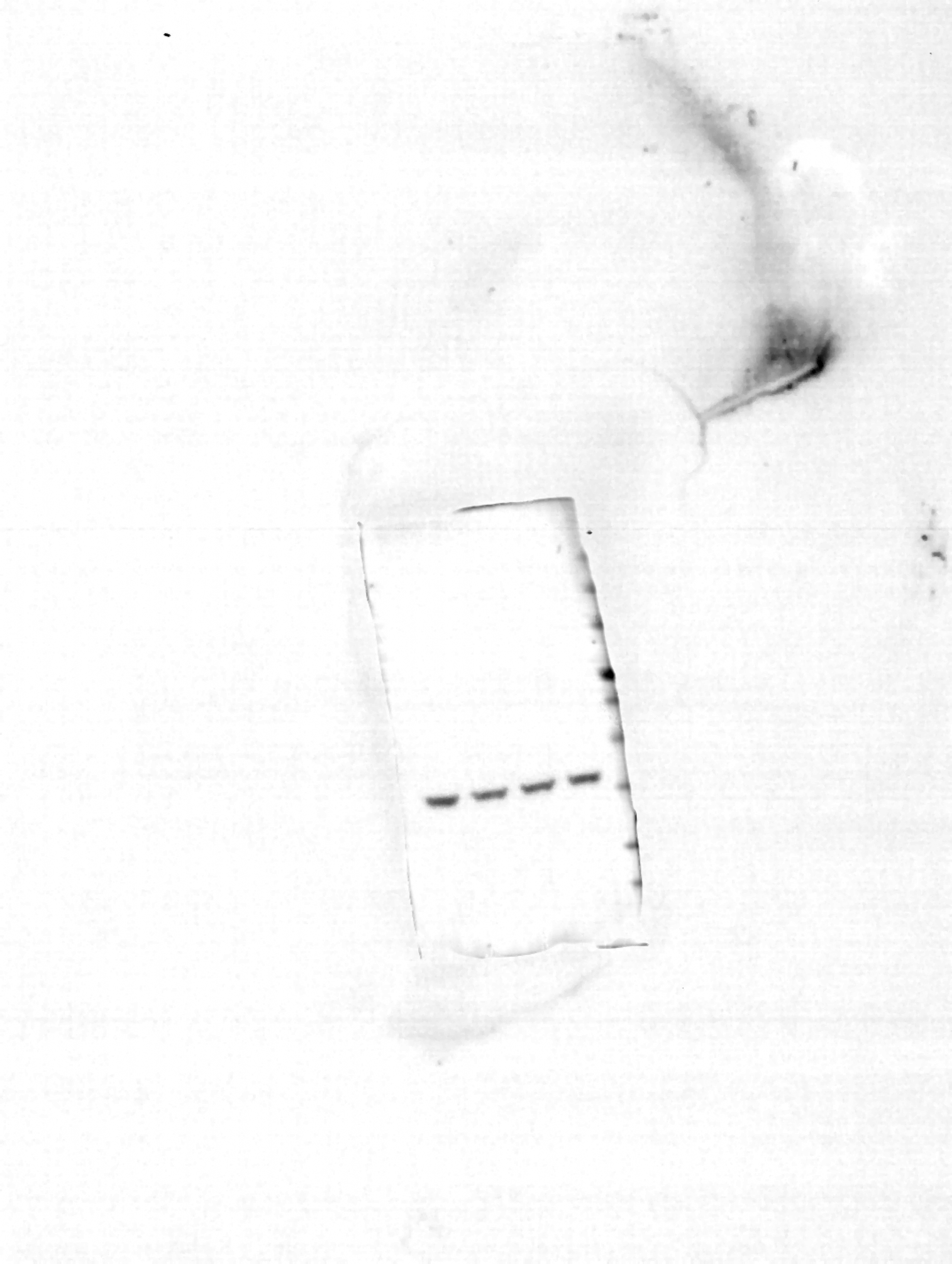

Supplement: Supplementary file 7 [file DataSheet5.zip › figure6 wb/figure 6 A gapdh 2.tif]

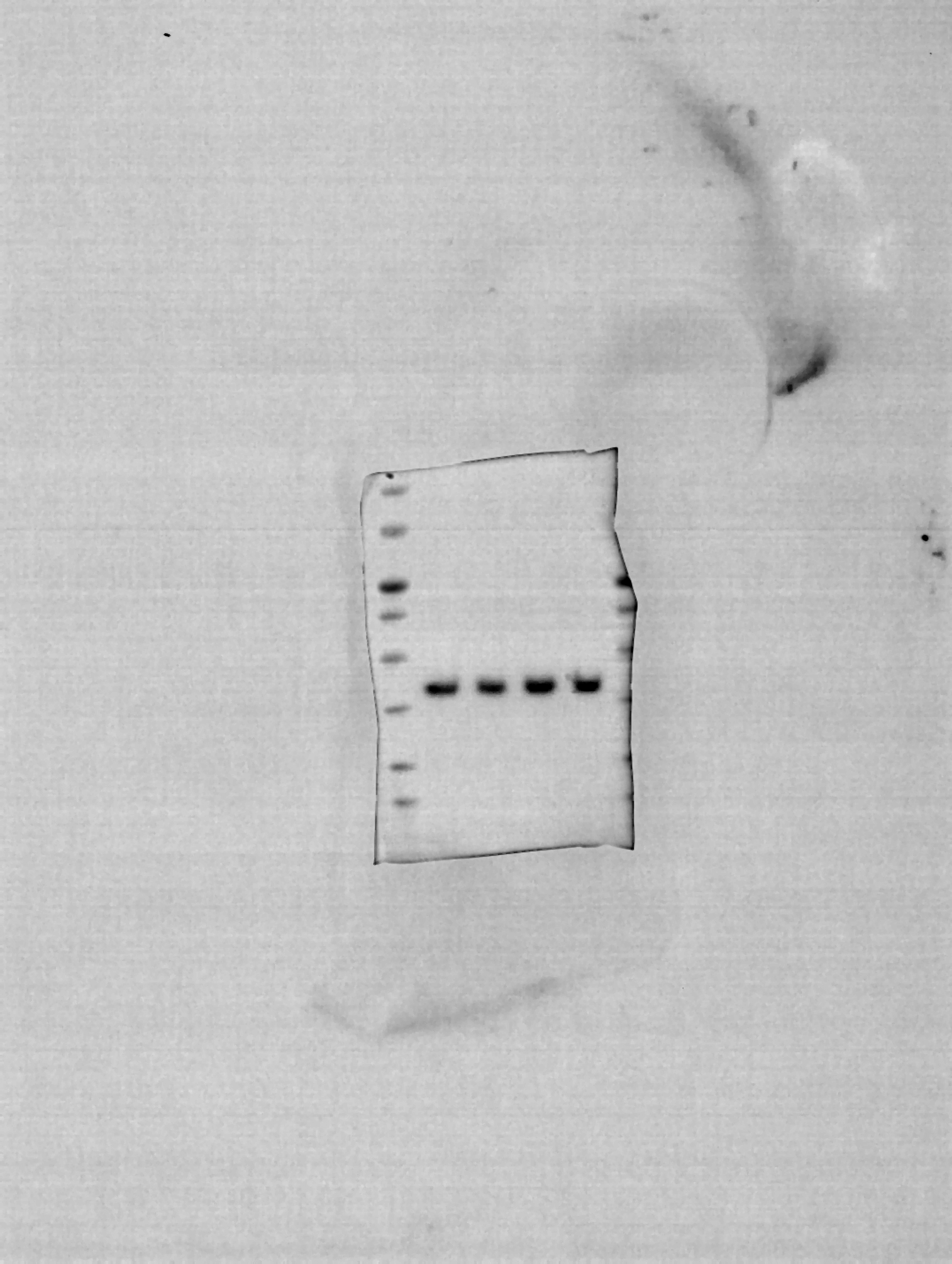

Supplement: Supplementary file 7 [file DataSheet5.zip › figure6 wb/figure 6 A gapdh 3.tif]

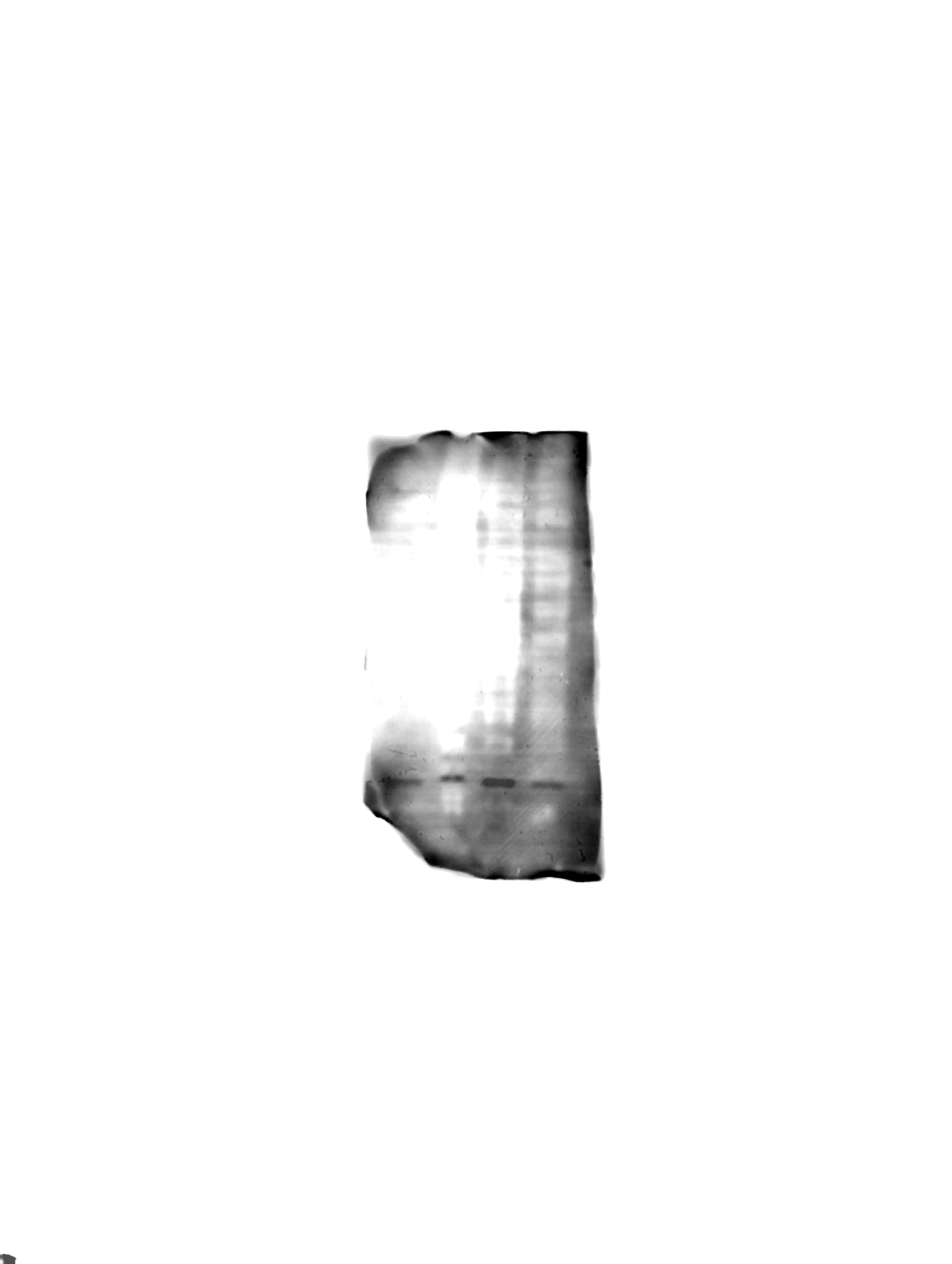

Supplement: Supplementary file 7 [file DataSheet5.zip › figure6 wb/figure 6 a dlat 2.tif]
