# Supplementary material for: Suppressing chondrocyte cuproptosis by syringaresinol-4-O-β-d-glucoside alleviates gouty arthritis
Source: Front Pharmacol. 2025 May 9;16:1565422. doi: 10.3389/fphar.2025.1565422 (PMC12099060; doi:10.3389/fphar.2025.1565422)
Supplement: Supplementary file 2 [file DataSheet7.docx]

Supplementary Material

# Supplementary Figures


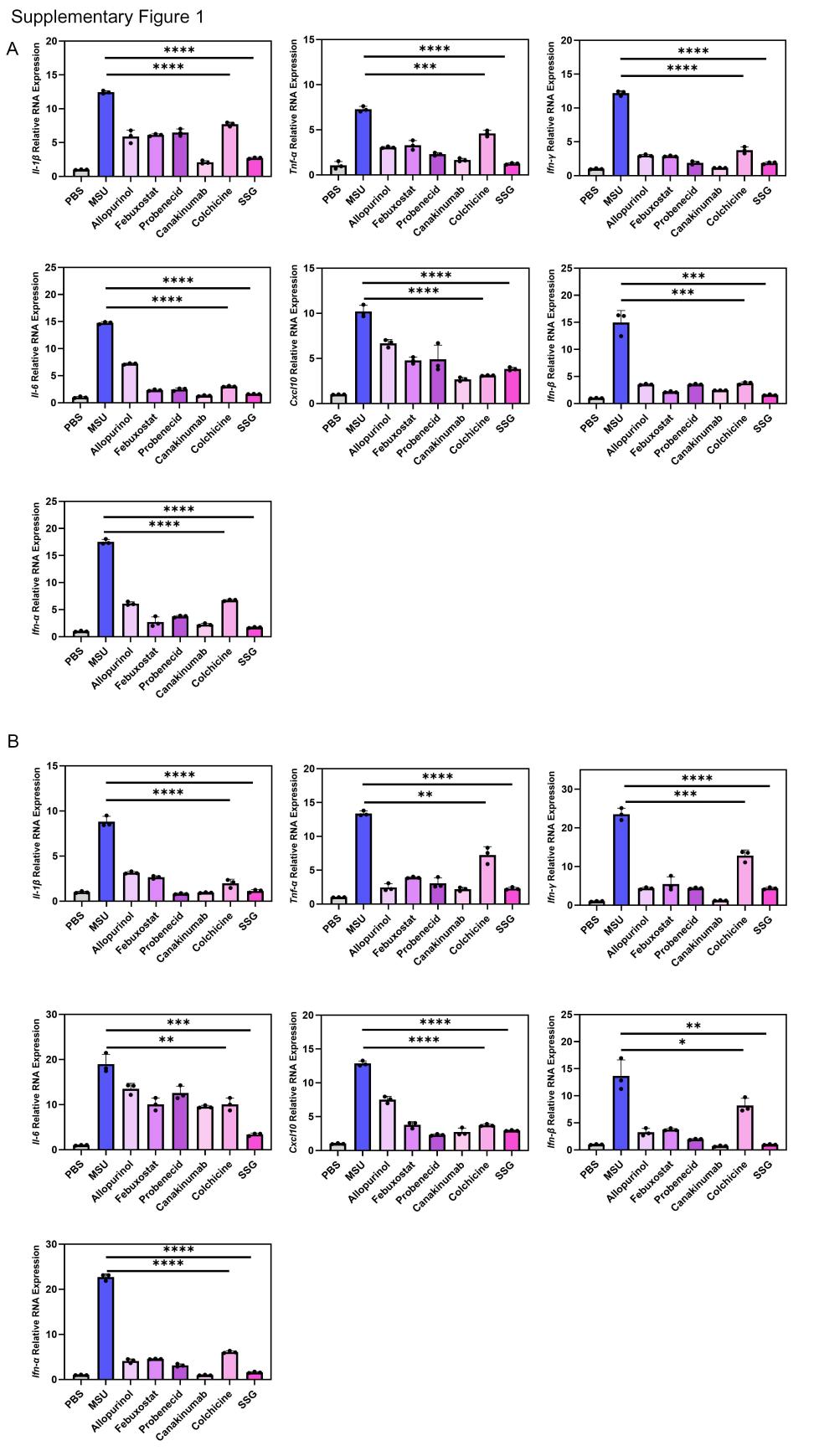


**Supplementary Figure 1**

The anti-inflammatory efficacy of SSG is superior to that of commonly used gout medications in clinical practice. (A) The gene expression levels of *Il-1β*, *Il-6*, *Tnf-α*, *Ifn-α*, *Ifn-β*, *Ifn-γ* and *Cxcl10* in RAW264.7 cells following different stimuli were evaluated using RT-qPCR (n=3). **(B)** The gene expression levels of *Il-1β*, *Il-6*, *Tnf-α*, *Ifn-α*, *Ifn-β*, *Ifn-γ* and *Cxcl10* in chondrocytes following different stimuli were evaluated using RT-qPCR (n=3). The bar graph represents the mean ± SD. *p < 0.05, **p < 0.01, ***p < 0.001 compared to the control group. (Drug concentrations: Allopurinol (0.2 mM), Febuxostat (100 nM), Probenecid (0.1 mM), Canakinumab (40 pM), Colchicine (0.1 μM), SSG (0.1 mM)).


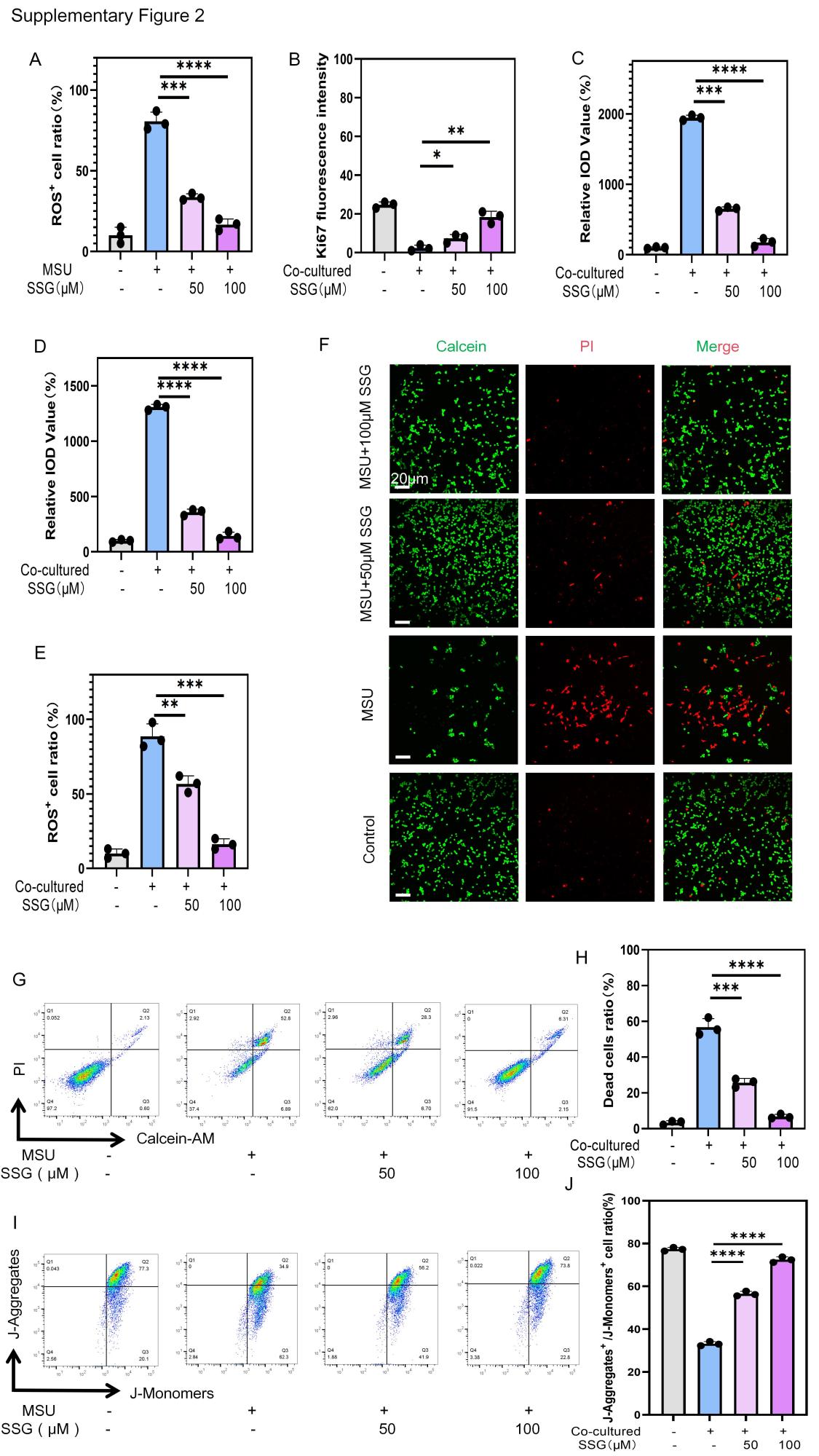


**Supplementary Figure 2**

SSG reduces cartilage necrosis and mitochondrial damage. **(A)** Quantifying ROS labeled positive cells shown in figure 4 E (n=3).  **(B)** Statistics of fluorescence intensity of Ki67 respectively (n=3). **(C)** Statistics of relative IOD value (%) of β-gal staining experiment respectively (n=3). **(D)** Statistics of relative IOD value (%) of ROS fluorescence in chondrocytes respectively (n=3). **(E)** Quantification of ROS labeled positive cells shown in figure 6 E (n=3). **(F)** Representative fluorescent images of chondrocyte death conditions. **(G)** Representative flow cytometry images of chondrocyte death. **(H)** Quantifying dead cells shown in panel E (n=3). **(I)** JC-1 assay is used to detect changes in mitochondrial membrane potential in macrophages. **(J)** The ratio of J-Aggregate^+^/J-Monomer^+^ cells in panel G (n=3). The bar graph represents the mean ± SD. *p < 0.05, **p < 0.01, ***p < 0.001 compared to the control group.


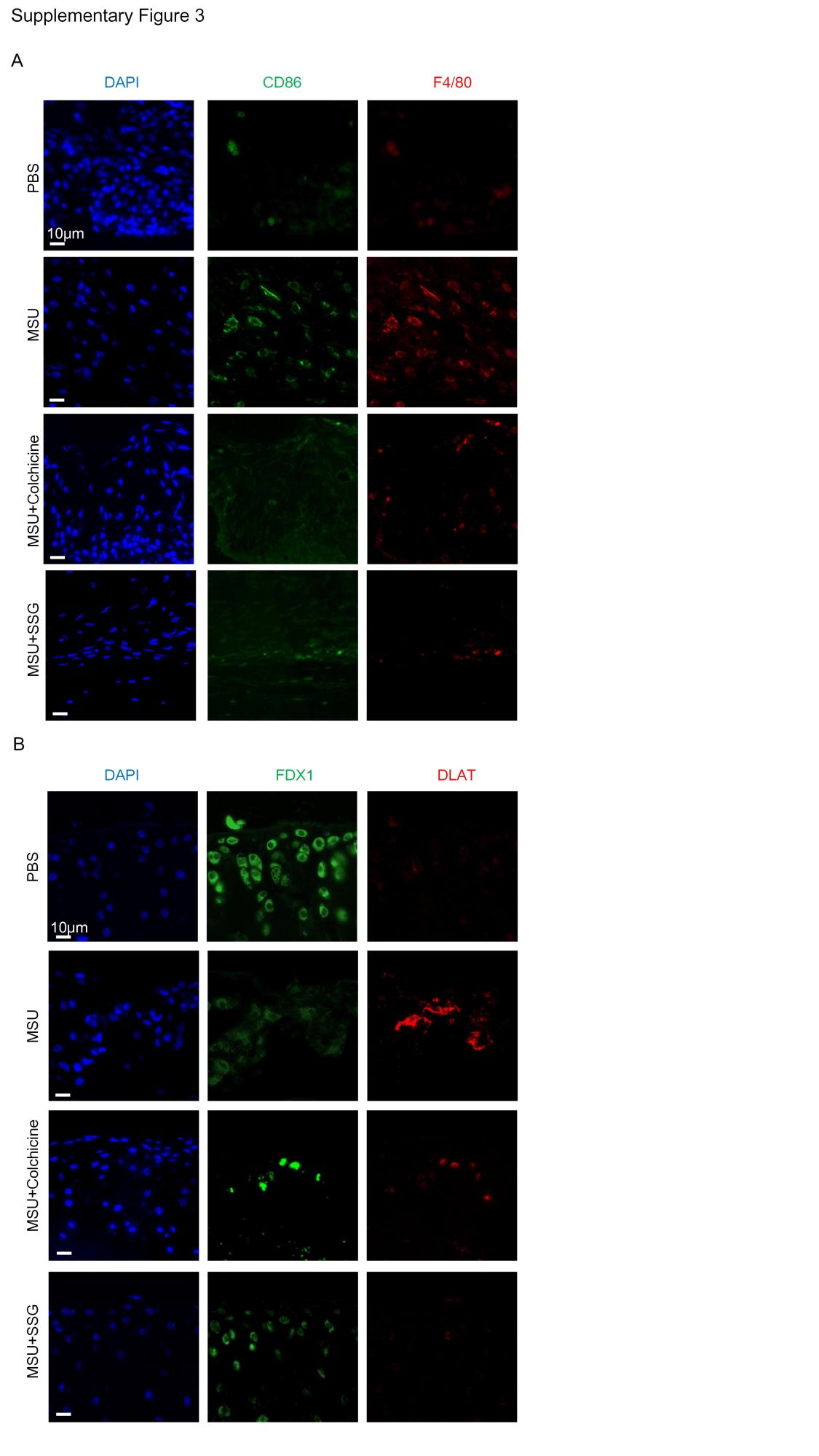


**Supplementary Figure 3**

**(A)** Representative images of double immunohistochemical staining for F4/80 and CD86 in mouse synovium. **(B)** Representative images of double immunohistochemical staining for DLAT and FDX1 in mouse articular cartilage.


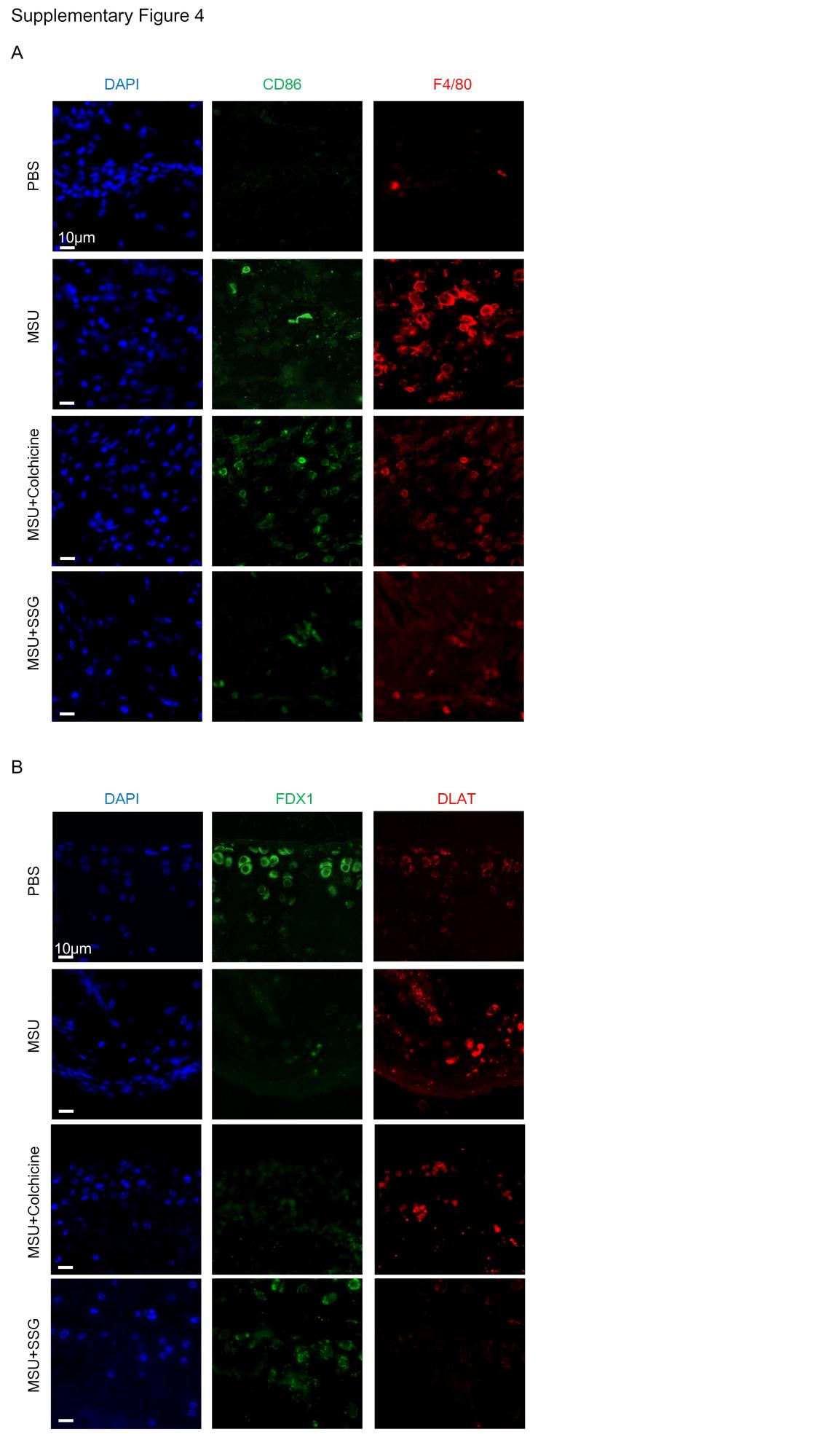


**Supplementary Figure 4**

**(A)** Representative images of double immunohistochemical staining for F4/80 and CD86 in mouse synovium. **(B)** Representative images of double immunohistochemical staining for DLAT and FDX1 in mouse articular cartilage.
